# Single-cell RNA-sequencing of circulating tumour cells: A practical guide to workflow and translational applications

**DOI:** 10.1007/s10555-025-10293-z

**Published:** 2025-10-06

**Authors:** Francis Yew Fu Tieng, Learn-Han Lee, Nurul-Syakima Ab Mutalib

**Affiliations:** 1 Department of Clinical Oncology, Faculty of Medicine, University of Malaya (UM), 50603 Kuala Lumpur, Malaysia; 2 Microbiome Research Group, Research Center for Life Science and Healthcare, Nottingham, Ningbo, China; 3 Beacons of Excellence Research and Innovation Institute (CBI), University of Nottingham Ningbo China, Ningbo, 315000 China; 4 UKM Medical Molecular Biology Institute (UMBI), Universiti Kebangsaan Malaysia (UKM), Cheras, 56000 Kuala Lumpur, Malaysia; 5 Novel Bacteria and Drug Discovery Research Group, Microbiome and Bioresource Research Strength, Jeffrey Cheah School of Medicine and Health Sciences, Monash University of Malaysia, 47500 Selangor, Malaysia

**Keywords:** Circulating tumour cells, Single-cell RNA sequencing, Cancer, Hybrid cells, Machine learning integration

## Abstract

The global burden of cancer is rising, with treatment failures often due to the metastatic nature of late-stage malignancies. Circulating tumour cells (CTCs) are metastatic precursors shed from primary tumours, which survive in circulation, extravasate and colonise distant organs. The advent of high-throughput single-cell RNA sequencing (scRNA-seq) has revolutionised the investigation of transcriptomic landscape at single-cell resolution, enabling deep transcriptomic profiling, re-stratifying CTC subtypes and improving the detection of rare new subpopulations. Applications extend to understanding tumour microenvironments, characterising cellular heterogeneity, uncovering metastasis molecular mechanisms and improving prognosis and diagnostic strategies. A timeline of key milestones in CTC scRNA-seq research is also provided. Nevertheless, a knowledge gap remains due to unstandardised protocols and fragmented resources in CTC scRNA-seq research. We address this gap by proposing a 12-step CTC-specific scRNA-seq workflow to overcome methodological inconsistencies. This workflow spans the entire process from enrichment, single-cell sorting and sequencing to data pre-processing and downstream analyses, with a detailed compilation of data analysis tools. An in-depth discussion of the pros and cons of commonly used scRNA-seq tools is also included, specifically evaluating their suitability for CTC research. Additionally, emerging research frontiers, including the discovery of hybrid cells—fusion products of tumour and normal cells—and the integration of machine learning (ML) into scRNA-seq workflows, are explored. Future research should prioritise CTC scRNA-seq workflow standardisation, integrate ML-driven analysis and investigate rare and hybrid populations to advance metastasis research. This review supports these goals by guiding methods, informing tool selection and promoting data sharing for reproducibility.

## Introduction

The landscape of cancer diagnosis has evolved significantly over the past decade, integrating liquid biopsies alongside traditional tissue biopsies [1]. Liquid biopsies have gained popularity due to their minimally invasive sampling procedures, which allow frequent collection and is particularly beneficial when tumour tissue is inaccessible or limited [2]. Moreover, they provide diagnostic and prognostic insights into solid tumours, offering the potential for early, accurate diagnostics and effective disease monitoring across diverse cancer types [3, 4]. Consequently, liquid biopsies are increasingly being integrated into clinical practice [5, 6].

Circulating tumour cells are rare cells released from primary tumour lesions into the bloodstream, serving as the precursors for metastasis [7]. They exhibit phenotypic plasticity, including the ability to undergo epithelial-mesenchymal transition (EMT), interacting dynamically with their microenvironment to enhance survival and metastatic potential [8, 9]. This results in a heterogeneous population that mirrors the complexity of the primary tumour, influencing cancer progression and treatment response [10, 11]. Of particular interest are hybrid cells, resulting from the fusion of neoplastic and immune cell characteristics. They further complicate the understanding of tumour heterogeneity and immune responses within the tumour microenvironment (TME) [12]. These hybrid cells represent a novel frontier in cancer research, and have significant implications for disease progression and therapeutic strategies [13, 14].

Single-cell RNA sequencing (scRNA-seq) is a powerful tool for dissecting metastatic processes driven by CTCs [15]. The transition from bulk to scRNA-seq represents a significant advancement in deciphering intratumoral heterogeneity (ITH) and phenotypic plasticity [16]. Unlike bulk sequencing, scRNA-seq provides insights into individual cell gene expression profiles. It can reveal intricate molecular networks that influence tumour heterogeneity and response to therapy [17–19]. Additionally, the integration of artificial intelligence and machine learning (ML) improves raw data processing for single CTCs, thereby enhancing CTC clustering, cell identification and the analysis of cellular heterogeneity [20, 21]. This knowledge advances precision oncology by enabling earlier detection and more personalized treatment strategies, which will ultimately improve outcomes for cancer patients.

## Technological advancements in CTCs scRNA-seq

The use of scRNA-seq to CTCs has evolved rapidly since the introduction of the Smart-seq protocol in 2012 [22]. In the following year, Smart-seq2 improved sensitivity and transcript coverage, sparking interest in CTC transcriptomics [23, 24]. By 2014, Ting and the researchers had identified distinct transcriptomic profiles of CTCs compared to primary tumours and tumour-derived cell lines. CTC clusters were found to exhibit low proliferation, enriched expression of *ALDH1A2* (stemness gene), *SPARC* (stromal ECM gene), *LGFBP5* (epithelial-stromal interface marker) and co-expression of epithelial and mesenchymal markers [25]. In 2016, the 10X Genomics Chromium system was introduced [26]. Innovations further included flow cytometry-guided CTC capture and scRNA-seq [27], scalable hydrodynamic Hydro-Seq barcoding system [28]; the adoption of locked nucleic acids in PCR whole transcriptome amplification to increase sensitivity [29]; and the development of SCR-chip—a microfluidic scRNA-seq platform using EpCAM^+^ immunomagnetic beads [30]. Most recently, the NICHE nanoplatform has enabled real-time, *in situ* gene expression and immune profiling of live CTCs [31]. Alongside this, the size-based MetaCell® technology provided a label-free approach to enrich viable CTCs from colorectal cancer (CRC) patient blood [32], while Stucky et al. established a robust clinical workflow for isolating CTCs from head and neck squamous cell carcinoma (HNSCC) patients before and during therapy, contributing to standardisation in CTC research [33].

**Fig. 1:**
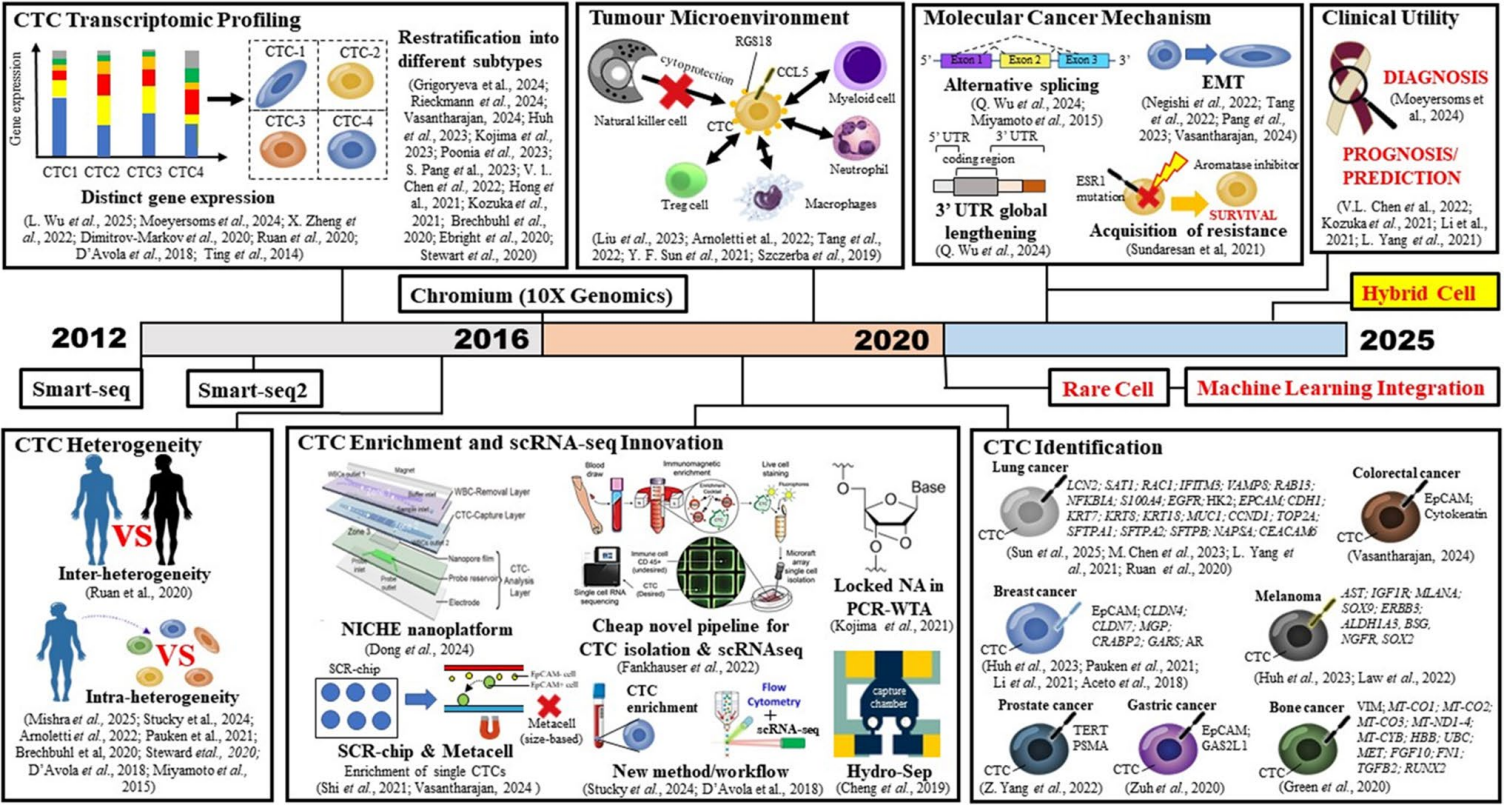
Timeline of technological milestones in CTC scRNA-seq from 2012 to 2024. It highlights foundational breakthroughs such as the introduction of Smart-seq (2012) and Smart-seq2 (2013), which significantly improved transcriptome coverage and sensitivity. Landmark study in 2014 revealed key transcriptomic differences between CTCs and primary tumours, highlighting stemness, EMT and ECM-related gene signatures. The 10X Genomics Chromium system (2016) enabled high-throughput profiling, followed by innovations such as flow cytometry-guided scRNA-seq, Hydro-Seq and whole transcriptomics analysis using locked nucleic acid. Biological insights progressed with studies on TME (2019), CTC identification, CTC subtype stratification (2020) and molecular cancer mechanisms (2021). Recent tools include the NICHE for *in situ* gene profiling, SCR-chip and label-free enrichment via MetaCell®. The timeline concludes with clinical standardisation efforts, including a validated CTC isolation workflow for HNSCC in 2024. This technological trajectory underpins the field’s readiness to tackle emerging frontiers—such as rare and hybrid cell discovery and machine learning–integrated CTC analysis

Figure 1 illustrates a chronological timeline of technological advancements and key applications of CTC scRNA-seq, laying the groundwork for the exploration of emerging functional insights—such as the discovery of rare and hybrid CTC populations, as well as the integration of ML in computational analyses.

## Clinical relevance of CTCs

### Molecular characterisation and phenotypic analysis

The identification of distinct gene expression signatures within CTCs via scRNA-seq represents the most fundamental level of molecular characterisation, a strategy that has been applied since as early as 2014 [25]. Beyond this, molecular characterisation permits the identification of genetic alterations and broader expression patterns [34, 35], while phenotyping strategies incorporate functional aspects, examining cellular behaviours and responsiveness to treatments [36, 37]. Both of these strategies are integral to defining transcriptomic profiles and enabling accurate clustering of CTC subpopulations. For instance, Grigoryeva et al. identified nine distinct integrin expression profiles from 42,225 CTCs from 81 non-metastatic breast cancer (BC) patients [38]. Similarly, three BC CTC clusters have been identified—estrogen receptor-positive (ER^+^), human epidermal growth factor receptor 2-positive and triple-negative—each exhibiting distinct expression profiles, including integrins, platelet degranulation markers and key oncogenes [39].

In non-small cell lung cancer (NSCLC), a large-scale study analysed 3363 single-cell CTC transcriptomes, revealing extensive phenotypic heterogeneity. They identified distinct clusters, including epithelial-like and proliferative (Cluster 1), cancer stem cell–like (Cluster 4), mesenchymal with oxidative phosphorylation and immune evasion (Cluster 5) and mesenchymal with invasive and glycolytic features (Cluster 6) [40]. In neuroblastoma, scRNA-seq has identified two CTC subgroups; Subgroup 1 was associated with increased proliferation and cell cycle-related features, whereas Subgroup 2 exhibited overexpression of neuronal injury-related genes (*FOS*, *RHOA* and *MIF*). Notably, higher CTC numbers were observed in patients with advanced-stage neuroblastoma [41]. In CRC, Kozuka et al*.* performed molecular characterisation on 59 single CTCs, revealing distinct gene expressions related to epithelial, EMT and stem cells within CTCs. The phenotypic classification of CTCs further contributes to a more refined and improved prognosis [42].

### Refinement of CTC heterogeneity

Over the past 5 years, CTC scRNA-seq research has surged remarkably, advancing the understanding of tumour heterogeneity. Studies have revealed that distinct CTC clusters often emerge based on patient-specific patterns, mirroring the robust intertumoral heterogeneity (eITH) observed across various cancer types [43, 44]. However, this diversity can mask subtle, yet significant patterns shared among CTCs obtained from a single tumour within a single patient (ITH) [45]. Conversely, there are intricate variations in both molecular and phenotypic differences within CTC clusters. Understanding them might shed light on both inter- and intra-tumoral heterogeneity, providing a comprehensive view of CTC diversity. Herein, Ruan et al*.* proved that scRNA-seq can quantify eITH and ITH within lung cancer metastases, both between patients and within a given patient. They attributed this variability to spatial differences among metastatic sites, variations in the expression of cell-cycle genes and cancer-testis antigens, as well as mesenchymal and circulating stem cell properties in CTCs. They also demonstrated that scRNA-seq could uncover potential temporal heterogeneity during tumour progression, offering further insight into ITH dynamics [46].

In the same year, Brechbuhl et al*.* revealed two distinct populations of CTCs from BC patients, differing in proliferative and EMT characteristics. Their study also highlighted immune evasion mechanisms, such as T cell exhaustion and PD-1/PD-L1 pathway activation, driven by CTC interactions with peripheral blood mononuclear cells (PBMCs) within the TME [47]. Pauken et al*.* identified unique circulating neoplastic cell populations in metastatic BC (mBC), revealing the significant role of genetic and epigenetic changes in ITH [48]. In 2022, clonal RNA expression variations within portal blood samples from each patient were first reported in pancreatic ductal adenocarcinoma (PDAC) [49]. Additionally, ITH characterisation in HNSCC found mutations in key signalling pathways, including CREB, β-Adrenergic receptor signalling and G-protein receptor signalling [33].

Similarly, two distinct CTC hierarchical clusters from two castration-resistant prostate cancer (CRPC) patients were discovered with the upregulation of disease progression pathways (AR, MYC, oxidative phosphorylation) and inflammatory response pathways (chemokine signalling, IL/JAK/STAT signalling) [50]. Collectively, understanding heterogeneity within CTCs unravels the complex landscape of CTC diversity, highlighting how dynamic interactions with the TME shape CTC behaviour, evolution and immune evasion.

### Dissecting CTC tumour microenvironment

The TME encompasses a dynamic ecosystem of various cell types, blood vessels, extracellular matrix and signalling molecules [51]. Leveraging scRNA-seq allows the dissection of this ecosystem at single-cell resolution, unravelling the intricate interactions between CTCs and their milieu [52]. Early evidence in BC showed that CTC-neutrophil clusters enriched in cytokine–receptor and cell–cell junction interactions promoted cell cycle progression and metastatic potential, pinpointing potential therapeutic vulnerabilities within the TME [53]. In hepatocellular carcinoma (HCC), spatially resolved CTC analysis across different vascular compartments uncovered transcriptional heterogeneity linked to stress response, immune evasion, and cell cycle signalling. These findings have highlighted CCL5 as a key player in immune escape and that spatial context is essential to dissect TME dynamics [54].

Similarly, Arnoletti et al*.* demonstrated that CTCs from PDAC contribute to immunosuppression and metastasis by promoting myeloid cell differentiation through CSF1R (colony-stimulating factor 1 receptor) and CXCR2 (CXC motif chemokine receptor 2) signalling pathways, mediated by M-CSF/IL-34 (macrophage colony-stimulating factor/interleukin 34) and IL-8, respectively. These interactions, as validated through *ex vivo* co-culture with myeloid fibroblasts, enhanced CTC proliferation and clustering, while blockading of these pathways impaired their metastatic potential [49]. Another study uncovered immune evasion mechanisms wherein PDAC CTCs interact with natural killer cells via the HLA-E: CD94-NKG2A checkpoint, aided by platelet-derived RGS18RGS18 (regulators of G-protein signalling 18) [55]. Complementing this, a multi-omics approach (scRNA-seq, WGCNA, CIBERSORT) revealed hallmark TIME features in metastatic PDAC CTCs, including EMT and cytoskeletal activation, elevated Treg and macrophage infiltration, and reduced dendritic cell proportions [56].

### Molecular mechanisms decoding EMT, metastasis and resistance

Metastatic progression signifies the final and most complex stage of tumour evolution. Noteworthy, it hinges on the delicate balance of the transition between epithelial and mesenchymal phenotypes, known as EMT [57]. Inherent CTC heterogeneity fuels increased proliferation, motility, and the acquisition of resistance. Exploring these traits at the single-cell level provides a granular understanding of metastatic behaviour and tumour adaptability. For example, a significant CTC subtype with EMT characteristics has been reported. It displayed platelet adhesion, contributing to EMT progression and the development of chemoresistance in metastatic human gastric cancer [58]. In early BC, CTCs with pronounced tumour cell characteristics (activated oxidative stress, proliferation and metastasis) have been subdivided into two subtypes. Subtype 1 exhibits strong metastatic ability due to its partial EMT phenotypes, whereas subtype 2 is linked to anti-apoptosis, EMT and migration properties [59]. Similarly, EMT-related pathways have been enriched in two CTC clusters identified in CRC (Vasantharajan, 2024).

Another scRNA-seq research has revealed heterogeneity in androgen receptor gene mutations and splicing variants among individual prostate CTCs, alongside divergent pathway activations that may underlie treatment failure [60]. In line with this, a recent multi-dataset analysis uncovered nearly 1000 alternative splicing events in single CTCs and CTC clusters, alongside a global 3′ UTR lengthening in BC CTC clusters driven by core polyadenylation factors such as PPP1CA—suggesting an additional layer of transcriptomic regulation shaping CTC phenotypes [61]. Moreover, single-cell profiling of a prospective cohort comprising 55 ER^+^ mBC women has revealed the presence of oestrogen receptor 1 mutations in isolated CTCs. This detection has further been correlated with the time to metastatic relapse and the duration of aromatase inhibitor therapy following such recurrence—highlighting their role in endocrine therapy resistance [62].

### Clinical translation of CTCs

After identifying the CTC subtypes and mechanisms, the subsequent phase is to translate these findings into clinical applications. Correlating specific CTC subtypes and molecular mechanisms with clinical outcomes help predict disease progression, treatment response and overall prognosis [63]. For example, Li and co-researchers identified 18 genes that were closely related to the specific CTC epithelial phenotype in BC, including a novel potential *glycyl-tRNA synthetase* (*GARS*) oncogene. They developed a risk score that correlated with high metastasis rates, poor survival, defective immune infiltration and low immunotherapy response. High-risk patients also showed greater sensitivity to AKT-mTOR and cyclin-dependent kinase (CDK) inhibitors [64]. In the same year, Yang et al*.* demonstrated that hexokinase 2 (HK2)-identified CTCs in NSCLC with EGFR mutations were linked to reduced EGFR inhibitor efficacy, enabling early prognosis and informing personalised therapy [65].

In a prospective melanoma cohort, CTC scRNA-seq revealed the coordinated upregulation of lipogenesis and iron homeostasis pathways, highlighting Sterol Regulatory Element-Binding Protein (SREBP)-driven mechanisms linked to cancer progression, drug resistance and poor prognosis, irrespective of treatment regimen in metastatic melanoma [36]. Moreover, two distinct CTC subgroups were identified, with one subgroup exhibiting elevated gene expressions related to epithelial phenotypes (*CHD1*, *EpCAM*, *ASGR2*, *KRT8*), EMT (*VIM*) and stemness (*CD133*, *POU5F1*, *NOTCH1*, *STAT3*). The presence of these CTC subgroups indicated a poorer prognosis in advanced HCC [66].

Table 1 presents a comprehensive overview of CTC scRNA-seq landscape across various cancer types, highlighting key research trends in enrichment methods, sequencing technologies and key findings. Breast, lung and pancreatic cancers are among the most frequently studied in CTC scRNA-seq research, with growing interest in prostate cancer and mesothelioma. All computational tools used or cited by the authors, as well as the specific scRNA-seq technologies employed in each study, have been included. This provides the foundation for the proposed scRNA-seq workflow detailed in Sect. 4.

**Table 1:** Overview of published scRNA-seq-related studies on CTCs across cancer types

Type of cancer	Year	CTC enrichment and single-cell sorting	scRNA-seq technology	Data analysis	Number of CTCs analysed	Finding	Cit
Breast cancer	2024	SRP131647: Parsortix® PC1 System (ANGLE plc), followed by micromanipulation [53]	Smart-seq2 (Takara Bio)	FastQC, STAR, salmon, outrigger	SRP131647: 92 single CTCs and 82 CTC clusters from ten BC patients and three xenografts; SRP133387: 66 single CTCs and 64 CTC clusters from matched donors, SRP066632: 41 single CTCs; SRP186111: 31 CTC clusters	▪ Four scRNA-seq datasets were used: SRP131647 (main), SRP133387, SRP066632, and SRP186111 (three validation sets) ▪ Identification of 994 and 836 alternative splicing events in single CTCs and CTC clusters, respectively ▪ Discovery of a global lengthening of 3′ UTRs in CTC clusters compared to single CTCs, governed by 14 core alternative polyadenylation factors, particularly PPP1CA	[61]
	2024	Negative enrichment via RosetteSep human CD45 depletion kit, followed by FACS (CD45^−^, EpCAM^+^, integrins β3, β4, and αVβ5)	Chromium system (10 × Genomics)	*Not stated*	42,225 CTCs from 81 non-metastatic BC patients	▪ Nine groups: o Group 1: 143 CTCs with non-complementary integrin α- and β-subunits o Group 2: 81 CTCs expressing only integrin α-subunits o Group 3: 8 CTCs with β1-containing heterodimers (no other β-subunits) o Group 4: 2 CTCs with β2-containing heterodimers (no other β-subunits) o Group 5: 11 CTCs with β3-containing heterodimers (no other β-subunits) o Group 6: 38 CTCs with multiple β-subunits and at least one heterodimer o Group 7: 3 3 CTCs with β4-subunit and other subunits/heterodimers o Group 8: 5 CTCs expressing full-fledged integrin α6β4 genes o Group 9: 154 CTCs without integrins gene expression	[38]
	2023	Immunomagnetic separation (CellSearch) and identification (CellCelector, Sartorius, Germany), followed by micromanipulation	QIAseq UPX 3′ transcriptome kit (QIAGEN)	CLC Genomics Workbench	24 pooled CTCs from two patient samples	▪ Workflow established for CTC enrichment, identification, and isolation without cell fixation/permeabilization, preserving viability and morphology ▪ Discrimination of apoptotic vs. non-apoptotic CTCs for downstream analysis	[67]
	2023	Integrated ClearCell FX system (size selection) and Polaris workflow (marker-free negative enrichment); control GSE144494: CTC-iChip microfluidic device (EpCAM^+^, Cadherin^+^, HER^+^), followed by micromanipulation	Fluidigm Polaris system (Fluidigm)	unCTC, FastQC, RSEM, bowtie, limma, DDLK custering, inferCNV	72 CTCs from 6 patients of three major subtypes: ER^−^/PR^−^/HER2^–^, ER^+^/PR^+^/HER2^−^, and ER^−^/PR^−^/HER2^+^; control GSE144494: 135 single CTCs or CTC clusters from 45 patients with HR^+^ mBC [68]	▪ Identification of three CTC clusters: ER^+^, HER2^+^, and triple-negative ▪ Molecular characterisation revealed: o Cluster 1: Upregulation of integrins (*ITGA2B*, *ITGB5*), platelet degranulation markers (*CLU*, *SPARC*), and oncogenes (*CDKN1A*, *TIMP1*, *PGRMC1*) o Cluster 2: Elevation in BC–associated transcripts (*IL10*, *BRIP1*, *IDO1*, *POU5F1*) o Cluster 3: Upregulation of *EPCAM*, *KRT18*, *KRT19*, *SOD1* ▪ Identification of several similar CNV patterns as in [68], including chromosome 1q associated with breast carcinogenesis and housing tumour suppressor genes and oncogenes	[39]
	2023	Immunomagnetic nanospheres (EpCAM^+^, CD45^–^)	Bead-dd-seq method [69]	*Not stated*	39 out of 90 CTCs from eight early BC patients with different lymph node status	▪ Identification of two CTC clusters: o Subtype 1: strong metastatic ability with partial EMT phenotypes and enriched transcripts in regulating the cell cycle and stemness (*TP53, E2F1, BRCA1, ESR1, SP1, MYC, KLF*4) o Subtype 2: anti-apoptosis, EMT and migration (*MUC4 and MUC16* expression)	[59]
	2023	CTC isolation kit (Cytogen, CIKW10) and SMART BIOPSY™ Cell Isolator (Cytogen, CIS030), followed by sorting via size exclusion based HDM (high density microporous) chip	Chromium system (10 × Genomics)	*Not stated*	Clinical sample: 55,580 and 15,142 single cells from blood and tissues from three BC patients; mouse model: 5,511 cells from primary tumours and 3,006 CTCs	▪ Identification of 21 EpCAM^+^ CTCs in blood and 1,488 EpCAM^+^ cancer cells in tissues from three BC patients ▪ Identification of three CTC clusters (cluster 4, 5, 7) consisting of epithelial and mesenchymal stem cells	[70, 71]
	2021	Hydrodynamic CTC sorting chip (EpCAM^+^, CD45^−^, CD15^–^); GSE144494: CTC-iChip microfluidic device (EpCAM^+^, Cadherin^+^, HER^+^), followed by micromanipulation	Smart-seq HT (Takara Bio)	GSEA	35 single CTCs from patients after endocrine therapy (8 CTCs from 3 patients with ESR1 mutations and 27 CTCs from 8 patients without ESR1 mutations); GSE144494: 135 single CTCs or CTC clusters from 45 patients with HR^+^ mBC [68]	▪ Detection of significant enrichment of CARM1-mediated ER signalling in wild type *ESR1* CTCs than mutant *ESR1* CTCs ▪ Evaluation of endocrine resistance via correlation of *ESR1* mutations with time to metastatic relapse and duration of AI therapy following such recurrence	[62]
	2021	FACS to obtain single cell suspensions, followed by validation via RareCyte Cytefinder II™ (CD45^−^, EpCAM^+^, CK^+^)	Chromium system (10 × Genomics)	Bcl2fastq, CellRanger, Seurat, DoubletFinder, SCtransform, FindNeighbors, FindCluster, singleR, MAST, ClusterProfiler	Lin^−^ and Lin^+^ cell populations from three mBC patients with different hormone receptor status *(number not stated)*	▪ Identification of 16 cell populations based on gene expression, where cluster 10 is CTC ▪ Upregulation of *CLDN4*, *CLDN7*, *MGP* and *CRABP2* in CTCs	[48]
	2021	GSE109761: Parsortix® PC1 System (ANGLE plc) (EpCAM^+^); validation GSE144494: CTC-iChip microfluidic device (EpCAM^+^, Cadherin^+^, HER^+^); followed by micromanipulation	GSE109761: Smart-seq2; GSE144494: smart-seq HT	Seurat, SingleR, limma, glemnet	116 single-cell CTCs datasets from GEO set GSE109761 [53]; validation GSE144494: 135 single CTCs or CTC clusters from 45 patients with HR^+^ mBC [68]	▪ Identification of eighteen genes closely related to the specific CTC epithelial phenotype BC patient prognosis ▪ Identification *GARS* gene, previously not studied in BC, as a potential oncogene ▪ Establishment of a risk score using the 18 genes as a prognostic and predictive marker	[64]
	2020	Filtration of whole blood via CellSieve filter (CREATV MicroTech, Inc), followed by filter backwash method	Chromium system (10 × Genomics)	CellRanger, Seurat	Patient 2 (11 CTC-1, 13 CTC-2), Patient 5 (13 CTC-1, 12 CTC-2), Patient 6 (0 CTC-1, 3 CTC-2), Patient 7 (1 CTC-1, 0 CTC-2), the combined patient samples 9, 10 and 11 (20 CTC-1, 10 CTC-2) and from the unfiltered local BC Patient 1 (7 CTC-1, 3 CTC-2)	▪ Identification of two populations of CTCs: o CTC-1 cluster: transcripts indicative of oestrogen responsiveness and increased proliferation o CTC-2 cluster: transcripts characteristic of reduced proliferation and EMT ▪ Prediction of increased immune evasion in the CTC population with EMT characteristics	[47]
	2020	CTC-iChip microfluidic device (EpCAM^+^, Cadherin^+^, HER^+^), followed by micromanipulation	Smart-seq HT (Takara Bio)	DESeq2	135 single CTCs or CTC clusters from 45 patients with HR + mBC; validation: 109 CTCs from 33 mBC patients	▪ Identification of a CTC subset with strong ribosome and protein synthesis signatures; proliferation and epithelial markers; and correlated with poor clinical outcome ▪ Exploration of CTCs as potential suppressors of metastatic progression	[68]
	2020	Microfluidic chip of scRNA-seq (SCR-chip) with immunomagnetic beads (EpCAM^+^)	Smart-seq v4 (Takara Bio)	PCA, t-SNE	14 single MCF-7 cells and 12 single white blood cells	▪ Design of SCR-chip for filtering blood clots, magnetic enrichment of CTCs, screening single CTCs, and obtaining single CTC RNA lysates ▪ scRNA-seq analysis revealed distinct genetic separation of CTCs and WBCs via PCA, tSNE, and marker genes, showing stable tumour-specific expression in CTCs and immune subtype diversity in WBCs, with no cross-contamination	[30]
	2019	Label-free microfluidic device (Labyrinth and Celsee PREP100 System)	Drop-seq	STAR, Drop-seq tools, Seurat, SNN, PCA, Enrichr	666 CTCs from 21 BC patient	▪ Development of Hydro-Seq, a scalable hydrodynamic scRNA-seq barcoding method, for high-throughput CTC analysis ▪ Identification of BC drug targets for hormone and targeted therapies and tracked individual cells that express markers of cancer stem cells as well as of epithelial/mesenchymal cell state transitions	[28]
	2019	Parsortix® PC1 System (ANGLE plc), followed by micromanipulation	Smart-seq2 (Takara Bio)	FastQC, FastQ Screen, MultiQC, Trim Galore, RSeQC, RefSeq, scran. t-SNE, scater, RCA	262 CTCs, 14 CTC-PBMC clusters and 82 PBMCs from 34 metastatic BC patients	▪ Identification of cell–cell junctions and cytokine–receptor pairs in CTC–neutrophil clusters as key vulnerabilities in the metastatic process ▪ Association between neutrophils and CTCs promoted cell cycle progression and enhanced the metastatic potential of CTCs, suggesting a therapeutic target for BC treatment	[53]
	2019	Parsortix GEN3D6.5 Cell Separation Cassette (Angle Europe), followed by micromanipulation	Smart-seq2 (Takara Bio)	FastQC, Trim Galore, bowtie2, RSeQC, MethylKit, SLIM, LOLA	Matched 48 CTCs and 24 CTC clusters within individual liquid biopsies from six breast cancer patients with progressive metastatic disease; and 49 CTCs and 54 CTC clusters isolated from three xenograft models	▪ Identification of cluster-specific gene modules linked to proliferation and cell–cell adhesion via transcriptome-wide weighted gene co-expression network analysis ▪ No significant modules were associated with single CTCs ▪ Ki67 immunostaining confirmed elevated proliferative activity in CTC clusters	[72]
	2018	CTC-iChip microfluidic device (EpCAM^+^, Cadherin^+^, HER^+^), followed by micromanipulation	ABI SOLiD protocol	DESeq2	13 patients with Bone (+) and nine patients with Visceral (+) disease, with a median of 3.5 CTCs per patient	▪ Identification of increased AR expression in BC with bone metastases	[73]
	2014	^neg^CTC-iChip, followed by staining (EpCAM^+^, HER2^+^, CDH11^+^, CD45^–^, CD14^–^, CD16^–^) and micromanipulation	ABI SOLiD protocol	*Not stated*	29 samples (15 pools of single CTCs and 14 CTC-clusters) isolated from 10 breast cancer patients	▪ Identification of the cell junction component plakoglobin as a highly expressed component in scRNA-seq profiling of CTC clusters and single CTCs	[74]
Pancreatic cancer	2023	Laparoscopy, followed by microfluidic chip to isolate CTC; validation via immunofluorescence staining and FACS (EpCAM^+^, CD45^–^)	Chromium system (10 × Genomics)	CellRanger, Seurat, Louvain, t-SNE, sciBet, inferCNV, CopyKAT, SingleCellSignatureExplore, limma, ClueGO, CellPhoneDB	300,000 to 500,000 FACS-sorted cells from primary, and liver metastatic lesions of six treatment-naive PDAC patients [75]	▪ Profiling of the cellular ecosystem in primary, CTC, and metastatic lesions ▪ Validation of interaction between CTCs and NK cells via the immune checkpoint molecule pair HLA-E:CD94-NKG2A ▪ Cytoprotection of CTCs from NK-mediated immune surveillance via platelet-derived RGS18	[55]
	2022	MoFlo FACS instrument with CyClone robotic adaptor (CD44^+^ CD147^+^ EPCAM^+^ CK^+^ CD45^−^)	Chromium system (10 × Genomics)	GeneWiz, limma, randomForest, ClusterProfile, factoextra, ReactomePA	35,338.46 ± 59,742.90SD/median 11,932 CTCs per million PBMC collected; representative CTC samples from 2 patients	▪ Identification of clonal RNA expression variation within each patient’s portal blood sample ▪ Identification of high level of *CXCL8*, indicating involvement of PDAC CTCs in myeloid cell differentiation to support survival and immuno-resistance in portal vein circulation	[49]
	2022	GSE114704: AutoMACS Pro Separator using MS Columns (Miltenyi Biotec) (HLA^–^ABC^+^), followed by C1™ Single-Cell Auto Prep System (Fluidigm)	GSE114704: Smart-seq	Seurat, PCA, t-SNE, Metascape tool	GSE114704: 10 CTCs, 23 liver metastasis cells, and 37 primary tumour cells derived from PDX mouse model [76]	▪ Identification of 87 marker genes highly associated with PDAC metastasis via scRNA-seq (GSE114704) ▪ Combination of scRNAseq with bulk RNA-seq (GSE144561) allows pinpointing cell-type-specific expression patterns in CTCs [77] ▪ Multi-omics approach (scRNA-seq + WGCNA + CIBERSORT) revealed high Treg and macrophage infiltration, lower dendritic cell proportions, and enrichment of EMT and cytoskeleton-related pathways in metastatic PDAC CTCs	[56]
	2020	CTC-iChip and anti-CD45 magnetic beads, followed by micromanipulation [25]	Modified single-cell amplification and library protocol by Tang et al., 2010 [78]	Seurat, PCA, t-SNE, g:Profiler	GSE51372: 75 CTC from the genetically engineered mouse model, 12 WBCs from a control mouse, 16 single tumour cells from NB508 cell line, 12 white blood cells, 18 GFP-traced CTCs, 20 EGFP-positive primary bulk tumour cells and 34 RNA dilutions from primary pancreatic tumours	▪ Identification of functionally enriched focal adhesion pathway in pancreatic CTCs ▪ GAS2L1 as a potential surface marker of pancreatic CTCs in combination with EpCAM ▪ Identification of three murine pancreatic CTC clusters (Cluster 0, 1, 2) with different biological functions	[79]
	2020	AutoMACS Pro Separator using MS Columns (Miltenyi Biotec) (HLA^−^ABC^+^), followed by C1™ Single-Cell Auto Prep System (Fluidigm)	Smart-seq	FastQ Screen, TopHat2, HTSeq-count, SCDE, GSEA	10 CTCs, 23 liver metastasis cells, and 37 primary tumour cells derived from PDX mouse model	▪ Identification of distinct expression profiles between CTCs, their matched primary and metastatic tumours ▪ Characterisation of CTCs with low expression of cell-cycle and extracellular matrix-associated genes ▪ Identification of new target: survivin (BIRC5) as a key regulator of mitosis and apoptosis in cancer metastasis	[76]
	2014	CTC-iChip and anti-CD45 magnetic beads, followed by micromanipulation	Modified single-cell amplification and library protocol by Tang et al., 2010 [78]	*Not stated*	75 CTC from the genetically engineered mouse model, 12 WBCs from a control mouse, 16 single tumour cells from NB508 cell line, 12 white blood cells, 18 GFP-traced CTCs, 20 EGFP-positive primary bulk tumour cells, and 34 RNA dilutions from primary pancreatic tumours	▪ Identification of distinct transcriptomic profile of CTCs from primary tumours and tumour-derived cell lines ▪ CTC cluster exhibit low proliferation, enrichment of *ALDH1A2* (stemness gene), *SPARC* (stromal ECM gene), *LGFBP5* (epithelial-stromal interface marker) and co-express epithelial and mesenchymal markers	[25]
Lung cancer	2024	Negative enrichment via magnetic MACS MicroBeads targeting CD3^+^, CD16^+^, CD31^+^, CD45^−^, and CD235a^+^ (Miltenyi Biotec), followed by FACSAria (BD Bioscience)	Chromium system (10 × Genomics)	FastQC, CellRanger, SingleR, scclusteval, Monocle2, Seurat, GSEA, inferCNV	9659 CTCs from six NSCLC patients	▪ Identification of 3,363 single cell CTC whole transcriptomes ▪ Identification of high degree of phenotypic heterogeneity and a variety of CTC phenotypes: o CTC cluster 1: epithelial-like, immune responsive and highly proliferative o CTC cluster 4: cancer-stem cell like o CTC cluster 5: mesenchymal, oxidative phosphorylation, and immune evasive o CTC cluster 6: mesenchymal, invasive, and glycolytic	[40]
	2024	Microfluidic cell trap arrays for WBC removal, CTC capture, and CTC analysis	On-chip single-cell gene expression analysis using fluorescence probes	NA	CTCs from 80 NSCLC patients	▪ Development of a nanoplatform for interrogating living cell host-gene and (micro-)environment (NICHE) for real-time, *in situ* CTC analysis, including gene expression and immune response profiling	[31]
	2023	Leukocyte negative enrichment using magnetic beads, followed by EpCAM^+^ positive staining and manual picking via mouth pipette	Smart-seq2 (Takara Bio)	Trimmomatic, HISAT2, FeatureCounts, Seurat, scater, KEGGprofile, Clusterprofiler, CytoScape, Monocle v2	124 single cells from primary tumour tissue (PTT), primary para-tumour tissue (PTP), metastatic tumour tissue (MTT), para-metastatic tumour normal brain tissue (MTP), and CTCs from a LUAD patient	▪ Identification of 16 CTCs, 30 MTPs, 43 MTTs. 13 PTPs and 22 PTTs from LUAD-PT1 patient ▪ Elevated levels of *LCN2, SAT1, RAC1, IFITM3, VAMP8, RAB13, NFKBIA* and *S100A4* (related to regulated cell death and apoptosis, promoted macromolecule organisation) in CTCs	[80]
	2021	RosetteSep enrichment, followed by staining (CK7/8, HK2^+^), on-chip imaging and single-cell manipulation	Chromium system (10 × Genomics)	CellRanger, UMAP, inferCNV, fgsea, limma, GSVA	16 randomly selected CTCs from 70 putative CTCs from patient 1 and 120 putative CTCs from patient 2	▪ Confirmation of the malignancy of HK2^+^ putative CTCs in pleural effusions and cerebrospinal fluids ▪ Prediction of NSCLC patients with poor prognosis before therapy via selective association of CK subtypes in CTCs with EGFR mutation	[65]
	2020	Isolation from blood samples on a Ficoll gradient, followed by FACS (HLA^–^ABC^+^, Calcein-AM^+^)	Chromium system (10 × Genomics)	BWA-MEM, VARSCAN2, TOPHAT2, HTSEQ, EdgeR, GSEA, ANNOVAR, Seurat, Cell Ranger, PCA, t-SNE, inferCNV	At least 3,500 cells from four CTC-derived xenografts models (MDA-SC39, MDA-SC68, HCI-008, MDA-SC4) and SCLC patient treatment-naïve (84 CTCs), relapsed samples (627 CTCs), and maximal response (1 CTC)	▪ SCLC CDX models are predominantly neuroendocrine, exhibiting strong *ASCL1* expression in both platinum-sensitive and -resistant cases, with one resistant model showing high *NEUROD1* and minimal *POU2F3/YAP1* ▪ Identification of five unique patient CTC clusters with increased heterogeneity in cisplatin-treated CDXs—with an EMT-enriched cluster (low *ASCL1* and *DLL3*) and treatment-specific clusters in PARPi and CHKi relapse—indicating varied resistance mechanisms	[37]
Colorectal cancer	2024	Size-based Metacell® technology [81]	NA	NA	CTCs from CRC patients *(number not stated)*	▪ Detection of CTCs and CTC subpopulations in 47.6% of CRC patients via MetaCell ▪ Characterisation of CTCs using canonical markers (EpCAM and cytokeratins) ▪ Identification of EMT-related pathways in two CTC clusters out of 14 clusters	[32]
	2022	FACS to obtain single cell suspensions	Chromium system (10 × Genomics)	CellRanger, scimpute, Seurat, PCA, harmony, SingleCellSignatureExplore, Monocle, GSVA	54,788 cells from patient-matched tissue samples (17 CTCs from a patient)	▪ Identification of CTCs with some shared tumour-specific marker genes (*TGFB1* and *SOX9*) with tumour cells in solid lesions ▪ Identification of CTC-specific signatures related to platelet activation, regulation of cell death, and cell adhesion	[82]
	2021	CD45-based immunomagnetic negative selection, fluorescence staining (CD45/50^–^), followed by manual pipetting	Smart-seq v4 (Takara Bio)	*Not stated (Quickbiology NGS analysis service)*	59 single CTCs from 27 mCRC patients	▪ Gene discovery for CTC phenotyping ▪ Classification of CTCs into four main groups by epithelial, EMT and stem cell-related gene expressions for CRC prognosis	[42]
Skin cancer	2023	CTC isolation kit (Cytogen, CIKW10) and SMART BIOPSY™ Cell Isolator (Cytogen, CIS030), followed by sorting via size exclusion based HDM (high density microporous) chip	Chromium system (10 × Genomics)	*Not stated*	1129 primary tumour cells, 1139 CTCs and 1630 metastatic tumour cells	▪ Identification of upregulated *AST* factors in CTCs, which were absent in the primary tumour cells	[70, 71]
	2022	Ficoll-paque density gradient separation, followed by CD45-based negative enrichment (EaspSep direct human CTC enrichment kit)	Smart-seq2 (Takara Bio)	FASTQC, TrimGalore, bowtie2, HISAT2, FetaureCounts, mixOmics	182 CTCs from seven patients (20 mL of blood per collection)	▪ Development of a novel inexpensive pipeline to isolate melanoma CTCs and perform scRNA-seq, demonstrating its feasibility	[83]
	2021	Microfluidic CTC-iChip isolation, followed by confirmation via melanoma lineage markers and micromanipulation	Modified Smart-seq2 protocol [84]	Linnorm, GSVA, ZINB-WaVE, DEseq2, GSEA, fgSEA	76 individual CTCs collected from 22 metastatic melanoma patients	▪ Discovery of two CTC clusters via hierarchical clustering analysis ▪ Identification of upregulated lipogenic programs, iron homeostasis signatures, proliferation, and increased energy production in CTC cluster 2 compared to cluster 1 ▪ Correlation of CTCs with high lipogenic and iron metabolic RNA signatures to adverse clinical outcome, irrespective of treatment regimen	[36]
Liver cancer	2022	Dual filtration system, followed by micromanipulation with micropipette	Smart-seq2 (Takara Bio)	STAR, Subread, Seurat, UMAP,	38 CTCs and 33 CTC clusters from 6 HCC patients	▪ Identification of two distinct CTC groups ▪ Upregulation of epithelial phenotypes (*CDH1*, *EPCAM*, *ASGR2*, *KRT8*), epithelial-mesenchymal transition (*VIM*), and stemness (*CD133*, *POU5F1*, *NOTCH1*, *STAT3*) in Group 1 as compared to Group 2	[66]
	2021	RosetteSep Human CD45 Depletion kit, followed by staining via EpCAM^+^, pan-CK^+^, CK19^+^, CCL^+^ and CD45^–^	Smart-seq2 (Takara Bio)	SOAPnuke, RSEM, Rt-SNE, edgeR, Monocle, HALLMARK	113 single CTCs from 4 different vascular sites, including hepatic vein, peripheral artery, peripheral vein and portal vein of HCC patients	▪ Transcriptional dynamics of CTCs were associated with stress response, cell cycle and immune-evasion signalling during hematogeneous transportation ▪ Identification of chemokine CCL5 as an important mediator for CTC immune evasion ▪ Discovery of a previously unappreciated spatial heterogeneity and an immune-escape mechanism of CTC	[54]
	2018	Immunodensity CD45 depletion, followed by imaging flow cytometry	Chromium system (10 × Genomics)	Seurat, nUMIs, PCA, t-SNE, CCA, GSEA	25 CTCs (5 CTCs from patient 1; 5 CTCs from patient 2; 11 CTCs from patient 3; 4 CTCs from patient 4)	▪ Description of a method that sequentially combines image flow cytometry and high density scRNA-seq to identify CTCs in HCC patients ▪ Identification of HCC driver genes revealed the molecular heterogeneity in CTCs	[27]
Prostate cancer	2025	Microfluidic-based negative depletion strategy (CTC-iChip: inertial separation array and MAGLENS), followed by two-step sorting using SONY SH800 sorter (bulk and single-cell); validation via EpCAM, PSMA, GPC3, ASGPR1, CD45, CD16, and CD66b	Smart-Seq2 (Takara Bio)	TrimGalore, bamToBed, Ginkgo website, samtools, TopHat, HT-Seq, GSEA	30 CTCs from patient GU-1 and 74 CTCs from patient GU-2	▪ Two distinct hierarchical clusters from patient GU-1: o Cluster-1: Upregulation of FGFR and AR signalling o Cluster-2: Upregulation of pathways associated with inflammatory responses, chemokine signalling and IL/JAK/STAT signalling ▪ Two distinct hierarchical clusters from patient GU-2: o Cluster-1: Upregulation of CRPC disease progression pathways (AR, MYC and oxidative phosphorylation) o Cluster-2: Upregulation of ion channels and neuroendocrine differentiation genes ▪ High CTC yields (mean 10,057 CTCs per patient; range 100 to 58,125) revealed considerable intra-patient heterogeneity	[50]
	2015	Dynabeads MyOne Streptavidin T1 (Invitrogen), followed by microfluidic CTC-iChip (EpCAM^+^, CDH11^+^, CD45^–^)	Modified ABI SOLiD protocol	TopHat	77 intact CTCs isolated from 13 patients	▪ Identification of heterogeneity among single CTCs, including variations in AR gene mutations and splicing variants ▪ Identification of heterogeneity in signalling pathways that could contribute to treatment failure	[60]
Head and neck cancer	2024	Positive enrichment with human CD326/EpCAM MicroBeads (Miltenyi Biotec)	Chromium system (10 × Genomics)	CellRanger, Seurat, UMAP, Ingenuity Pathway Analysis (QIAGEN)	1,000 EpCAM^+^ CTCs from four patients with biopsy-proven HNSCC	▪ Identification of CTC heterogeneity within a single individual ▪ Identification of CTC mutations in the CREB signalling pathway, β-Adrenergic receptor signalling and G-protein receptor signalling pathway ▪ Establishment of a workflow to isolate CTCs from HNSCC patients’ blood samples before and during cancer treatment	[33]
	2024	Isolation of PBMC fraction via centrifugation, followed by RBC lysis	Chromium system (10 × Genomics)	CellRanger	4,852 PBMCs, including CTCs	▪ Identification of a CTC cluster expressing MYB proto-oncogene ▪ Development of a sensitive, cost-effective, and minimally invasive diagnostic test that leverages tumour-specific signatures to screen for metastatic ACC disease	[34]
Neuroblastoma	2023	FACS Aria II cell sorter (BD Bioscience) (GD2^+^, CD90^+^, CD45^–^, CD235a^–^, DAPI^–^)	Smart-seq HT (Takara Bio)	CLC Genomics Workbench, Strand NGS	42 single CTCs from five neuroblastoma patients	▪ Higher CTCs in patients from advanced stages ▪ Identification of upregulated genes related to angiogenesis and cell cycle ▪ Identification of 2 subgroups from 20 CTCs: o Subgroup 1: proliferative and cell cycle-related o Subgroup 2: overexpression of neuronal injury-related genes (*FOS*, *RHOA*, *MIF*)	[41]
	2021	FACS Aria II cell sorter (BD Bioscience) (GD2^+^, CD90^+^, CD45^–^, CD235a^–^, DAPI^–^)	Smart-seq HT (Takara Bio)	CLC Genomics Workbench, Strand NGS	1 × 10^6^ TGW human neuroblastoma cells	▪ Three different whole-transcriptome amplification methods were conducted prior to scRNA-seq ▪ Validation of the use of locked nucleic acid technology in PCR-based WTA as an effective tool to amplify mRNA from a single cell accurately ▪ Introduction of a more dependable and flexible method for profiling CTCs at the single-cell level	[29]
Mesothelioma	2025	Enrichment of MSLN^+^CD45^−^ CTC from blood via MACS column and a microfluidic chip [85]	Chromium system (10 × Genomics v2)	Loupe Cell Browser v5.0.0, GSEA, WebGestalt V1.0, InteractiVenn, GSVA, GSCA	489 CTCs, 7662 *in vitro* mesothelioma cultured cells (CC) and 2,170 peritoneal lavage tumour cells (Lav)	▪ Identification of unique characteristics based on origin: o CTC: Upregulation of genes cancer cell stemness genes (*Ppbp, Gp9, Clec11b*) o CC: Upregulation of cell cycle control, proliferation, and apoptosis genes o Lav: Upregulation of microenvironment modulation genes (e.g., EMT and IFN-α/IFN-γ immune responses) ▪ Shared pathways indicated the potential for transitioning between functional states under specific conditions	[35]
Gastric cancer	2022	Mounting of PBMCs in a spacer seal affixed at a slide, followed by micro-manipulation of CD45^−^ cells	Quartz-Seq (Illumina)	Trimmomatic, RSeQC, Seurat, DAVID	49 CTCs from 26 patients and 12 single cells from cell lines (3 AGS, 3 NCI-N87, 6 SNU-1)	▪ Identification of majority gastric CTCs with EMT in metastatic cancer ▪ Contribution of platelet adhesion toward EMT progression and acquisition of chemoresistance	[58]
Bone cancer	2020	Manual pipetting of VIM^+^ CD45^–^	Smart-seq v4 (Takara Bio)	Trim Galore, HISAT2, Kallisto, DESeq2, rMATS, STRING, WGCNA	< 500,000 mapped reads in single osteosarcoma CTCs and > 30 million mapped reads in whole tumours	▪ Implication of a newly discovered, multidimensional MAPK7/MMP9 signalling hub in primary bone cancer metastasis ▪ Identification of single osteosarcoma CTCs positive for cell surface vimentin and negative for CD45 ▪ scRNA-seq revealed mitochondrial enrichment (*MT-CO1, MT-CO2, MT-CO3, MT-ND1-4, MT-CYB*), stress adaptation (*HBB, UBC*), stemness (*MET, FGF10, FN1, TGFB2, RUNX2*), extracellular matrix remodeling, and downregulation of metastasis suppressor (*BAP1*)	[86]

*ACC* adenoid cystic carcinoma, *AI* aromatase inhibitor, *AML* acute myeloid leukemia, *AR* androgen receptor, *BC* breast cancer, *BIRC5* baculoviral inhibitor of apoptosis repeat-containing 5/survivin, *CDX* CTC-derived xenografts, *CLDN* Claudin, *CRABP2* cellular retinoic acid binding protein 2, *CRC* colorectal cancer, *CRPC* castration-resistant prostate cancer, *CTC* circulating tumour cells, *DTCs* disseminated tumour cells, *EMT* epithelial-mesenchymal transition, *EpCAM* epithelial cell adhesion molecule, *ESC* embryonic stem cell, *ESR1* oestrogen receptor 1, *FGFR* fibroblast growth factor receptor, *GARS* glycyl-tRNA synthetase, *HK2* hexokinase-2, *HNSCC* head and neck squamous cell carcinoma, *IFN* interferon, *LMD* leptomeningeal disease, *LUAD* lung adenocarcinoma, *LUAD*-*LM* lung adenocarcinoma leptomeningeal metastases, *LUSC* lung squamous-cell carcinoma, *MAGLENS* magnetic lens-based high-throughput cell sorter, *mBC* metastatic breast cancer, *mCRC* metastatic CRC, *MGP* matrix Gla protein, *NK* natural killer, *NSCLC* non-small cell lung cancer, *PBMC* peripheral blood mononuclear cell, *PDX* patient-derived xenograft, *PPP1CA* serine/threonine-protein phosphatase PP1-alpha catalytic subunit, *RBC* red blood cell, *SCLC* small cell lung cancer, *SKCM* skin cutaneous melanoma, *Smart*-*seq* switch mechanism at the 5′ end of RNA template sequencing, *SOLiD* sequencing by oligonucleotide ligation and detection, *SOX9* SRY-box transcription factor 9, *TGFB1* transforming growth factor beta 1, *WTA* whole transcriptome amplification

## General workflow of CTC scRNAseq

Despite advancements in scRNA-seq technologies, most CTC-focused studies have remained confined to basic transcriptomic profiling and subtype identification with limited clinical or functional translation. This is due to the intrinsic rarity of CTCs (approximately 1 CTC per 10^7^–10^9^ haematological cells per mL), low separation efficiency, poor recovery rates and the lack of a universal CTC-specific surface marker/panel [87–92]. Additionally, many studies fail to disclose complete methodological details, making them difficult to reproduce. Even when raw datasets are made publicly available, they frequently lack standardisation—differing in gene lists, file formats, data orientation and annotation—further hindering reuse and cross-study comparisons. These combined technical and biological hurdles have created a bottleneck, restricting most investigations to molecular characterisation and subtyping instead of functional validation or therapeutic explorations. Notably, the number of published scRNA-seq studies on CTCs peaked around 2021 and has since declined, likely due to these unresolved limitations.

**Fig. 2:**
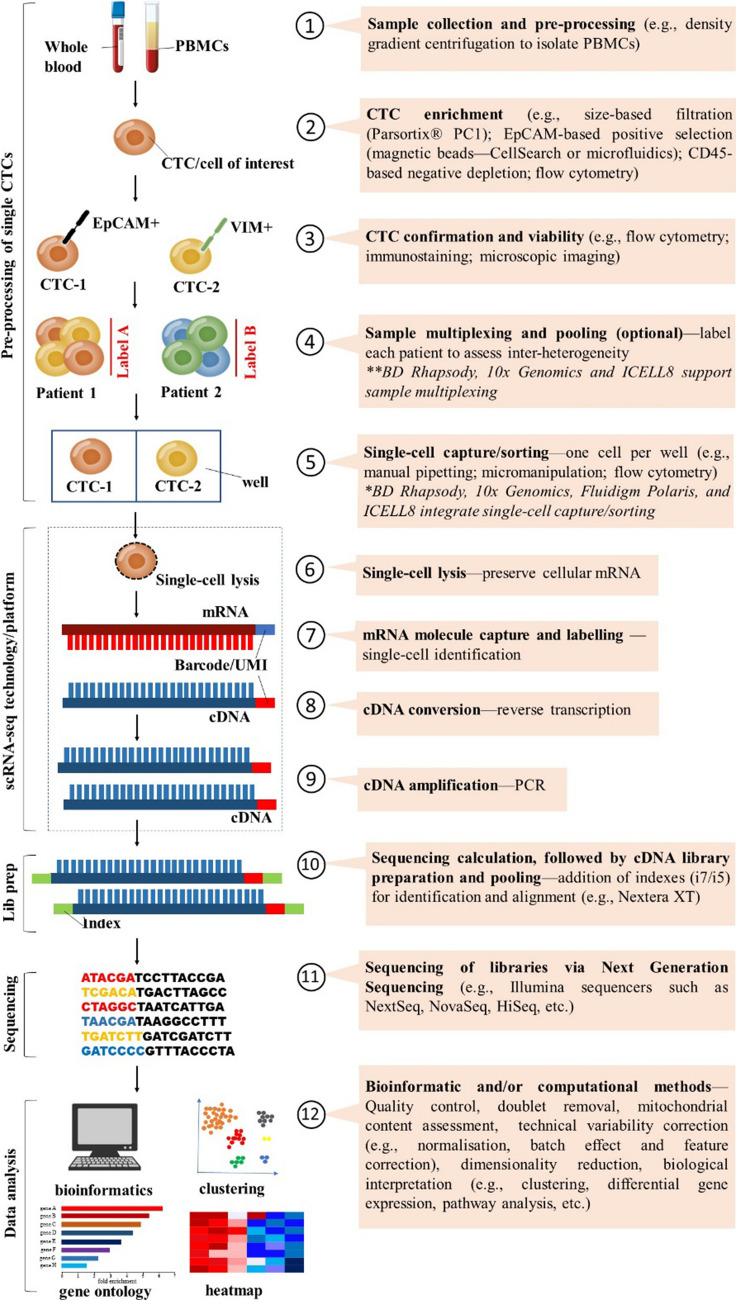
A practical twelve-step workflow for CTC specific scRNA-seq. Steps 1–5: pre-processing of single CTCs includes sample acquisition, CTC enrichment (marker-dependent or -independent), confirmation using tumour-specific markers, sample multiplexing and single-cell sorting. Steps 6–9: scRNA-seq platform-dependent processing covers single-cell lysis, mRNA capture and barcoding, reverse transcription into cDNA and cDNA amplification. Step 10: library preparation prepares amplified cDNA into sequencing-ready libraries. Step 11: next generation sequencing using high-throughput sequencers such as Illumina NextSeq, NovaSeq and HiSeq. Step 12: bioinformatic analysis involves quality control, alignment, count matrix generation, doublet removal, technical variability corrections and dimensionality reduction as well as biological interpretation (e.g. clustering, differential gene expression, pathway analysis, etc.)

To overcome these persistent limitations, a standardised twelve-step workflow for CTC-specific scRNA-seq studies (Fig. 2) is proposed. It spans from sample acquisition to biological interpretation and is grouped into five major phases. Steps 1–5 cover pre-processing of single CTCs, including sample processing, CTC enrichment (marker-dependent or -independent), phenotypic confirmation, multiplexing and single-cell sorting. Steps 6–9 constitute platform-dependent molecular processing, such as single-cell lysis, mRNA molecule capture and barcoding, cDNA synthesis and amplification. Step 10 involves cDNA library preparation, followed by step 11, which uses high-throughput sequencers (e.g. Illumina NextSeq, NovaSeq or HiSeq). Finally, step 12 encompasses bioinformatic analysis—covering quality control, alignment, count matrix generation, doublet detection, correction of technical artifacts (e.g. batch effects, cell cycle) and downstream interpretation including clustering, differential expression and pathway analysis.

In short, each step—from enrichment to data interpretation—must be supported by reliable methods and platforms to fully unlock the workflow’s potential. The following subsections explore each component in detail.

### CTC Enrichment

The first and most important step in CTC scRNA-seq is the effective isolation of viable single CTCs from a patient’s blood. Traditionally, this involves processing whole blood to isolate PBMCs, from which CTCs are subsequently enriched [93]. However, recent approaches increasingly favour direct processing of whole blood, such as microfluidic-based technologies like the CTC-iChip, to reduce cell loss and preserve CTC integrity [94]. Currently, only two FDA-approved methodologies are available for CTC detection and enrichment, namely CellSearch® (Veridex) [89] and Parsortix® PC1 (ANGLE plc) [95]. The CellSearch® system relies on positive enrichment strategy that targets CTCs of epithelial origin (CD45^–^, EpCAM^+^ and cytokeratins 8^+^, 18^+^ and/or 19^+^), whereas the latter employs a label-independent, size and deformability-based microfluidic separation strategy. Despite being the most widely adopted platforms in CTC research, CellSearch® may overlook mesenchymal or stem-like CTC subtypes due to its reliance on epithelial markers [89], while Parsortix® can be constrained by CTC size variability, potentially affecting capture consistency [95].

As a result, there has been a shift toward negative enrichment strategies, which aim to isolate CTCs by depleting CD45^+^ haematopoietic cells, thereby enabling the broader capture of phenotypically diverse CTC populations [96].

CTC enrichment techniques can be categorised into three main approaches: biophysical isolation, positive enrichment and negative enrichment. Biophysical isolation is label-free and is applicable to various cancer types, but may miss smaller or deformable CTCs. Positive enrichment offers high specificity by targeting specific CTC surface markers but may overlook other subtypes. Negative enrichment depletes immune cells, enabling broader CTC capture, though it often suffers from higher contamination and reduced purity. Therefore, researchers must carefully select an enrichment strategy based on both their specific research objectives and the phenotypic characteristics of the CTCs they wish to isolate.

Table 2 provides an overview of the three primary CTC enrichment strategies prior to scRNA-seq, their associated surface markers, applicable cancer types and current methodologies. Notably, there is often overlap among these strategies, as certain markers and techniques may be relevant across multiple cancer types or enrichment protocols. Following enrichment, choosing the appropriate scRNA-seq platform becomes critical to ensure transcriptomic fidelity and sensitivity, particularly for rare CTC populations.

**Table 2:** Blood-based CTC enrichment techniques for scRNA-seq studies

CTC enrichment	Type of cancer	Surface marker	Method
Biophysical isolation	Breast	–	Size (Parsortix® PC1; microfluidic; filtration-CellSieve; ClearCell FX system), density (high density microporous chip)
	Lung	–	Density gradient centrifugation
	Colorectal	–	Size (Metacell®)
	Skin	–	Density gradient centrifugation
	Liver	–	Density gradient centrifugation, size/dual filtration
	Prostate	–	Microfluidic CTC-ichip (inertial separation array and MAGLENS)
Positive enrichment (CTC cell surface marker)	Breast	EpCAM, cadherin, HER	Immunomagnetic beads/nanospheres (CellSearch; SCR-chip), microfluidic CTC-ichip, hydrodynamic CTC sorting chip
	Pancreas	CD44, CD147, CK, EpCAM, ABC	FACS, immunomagnetic beads (MACS)
	Lung	EpCAM, CD3, CD16, CD31, CD325a, CK7, CK8, HK2, ABC	Immunomagnetic beads (RosetteSep), Immunomagnetic beads (MACS)
	Prostate	EpCAM, CDH11	Microfluidic CTC-ichip
	Head and neck	EpCAM	Immunomagnetic beads (MicroBeads)
	Neuroblastoma	GD2, CD90	FACS
	Mesothelioma	MLSN	Immunostaining and manual pipetting
	Bone	VIM	Immunostaining and manual pipetting
Negative enrichment (immune cells depletion)	Breast	CD45, CD15	Immunomagnetic beads/nanospheres (RosetteSep), microfluidic CTC-ichip, hydrodynamic CTC sorting chip immunostaining and manual pipetting
	Pancreas	HLA, CD45	Immunomagnetic beads (MACS)
	Lung	HLA	Immunomagnetic beads, microfluidic CTC-ichip
	Colorectal	CD45	Immunomagnetic beads
	Skin	CD45	Microfluidic CTC-ichip
	Liver	CD45	Immunomagnetic beads (RosetteSep)
	Head and neck	CD45	Density gradient centrifugation
	Gastric	CD45	Immunostaining and manual pipetting

*ABC* ATP-binding cassette, *CD* cluster of differentiation, *CK* cytokeratin, *CTC* circulating tumour cells, *EpCAM* epithelial cell adhesion molecule, *FACS* fluorescence-activated cell sorting, *GD2* disialoganglioside 2, *HER* human epidermal growth factor receptor, *HK2* hexokinase 2, *HLA* human leukocyte antigen, *MACS* magnetic-activated cell sorting, *MLSN* mesothelin, *VIM* vimentin

### scRNA-seq platform comparison

Following CTC enrichment and validation using tumour-specific markers, single-cell sorting is a critical prelude to scRNA-seq. While most commercial platforms incorporate this within their workflows, methods such as Quartz-Seq [97, 98] and Smart-seq [22–24, 99] typically require pre-isolated single cells, which are typically prepared via manual pipetting or fluorescence-activated cell sorting (FACS). Depending on the experimental needs, researchers may also opt for alternatives such as automated micromanipulation, flow cytometry-based cell sorting, droplet microfluidics, and microfluidic or nanowell-based systems. Selecting the appropriate scRNA-seq platform hinges on key factors including throughput, capture rate, sensitivity, methods for Unique Molecular Identifier (UMI), cell barcoding, as well as compatibility with rare cell types like CTCs.

#### Full-length transcript method

Quartz-Seq and its updated version Quartz-Seq2 combine template-switching PCR with high UMI conversion efficiency (in the latter). They can also achieve full-length coverage and high sensitivity. Quartz-Seq2 is suitable for large-scale transcriptomics, with high reproducibility and detection of rare populations. However, these methods do not integrate single-cell sorting and are prone to PCR bias, preferentially amplifying shorter transcripts. Additionally, they require separate library prep kits such as Nextera XT [97, 98].

Like Quartz-Seq, Smart-seq protocols provide full-length transcript coverage, high sensitivity and low dropout rates—ideal for profiling rare cells and detecting splice variants or mutations. Smart-seq2 offers high mappability and transcript coverage from as little as 50 ng of starting RNA, with good reproducibility. Smart-seq3 adds 5′-end counting with UMI for improved quantification. However, these protocols are low-throughput, labour-intensive, and more expensive per cell. They also suffer from amplification bias and lack strand specificity, limiting scalability for large cohorts [22–24, 99].

Manual pipetting or micromanipulation may be incorporated to offer nearly perfect (~ 100%) capture accuracy, and is ideal for extremely rare cell types, but it is labour-intensive and time-consuming, and thus lacks scalability. These techniques are often paired with Smart-seq protocols for targeted applications requiring maximum resolution [22–24, 99].

#### Droplet-based platform

10 × Genomics Chromium is a droplet-based system which supports high throughput (up to > 20,000 cells with GEM-X technology), robust UMI-based quantification, and automated barcoding workflows. It offers a moderate capture efficiency (~ 30–80%) and is ideal for profiling larger populations. However, its reliance on 3′-end counting can lead to dropouts and limited detection of low-RNA content cells, making it less than optimal for rare or low-input cell types like CTCs without prior enrichment [26].

Drop-seq, developed by McCarroll lab, is another droplet-based system offering an economical, scalable option with the ability to process ~ 10,000 cells/day at a very low cost per cell (~ $0.07). Like 10× Chromium, Drop-seq incorporates barcoded beads and UMIs to individually label transcripts during cell lysis within nanoliter droplets. However, it suffers from a low capture efficiency (< 10%), which limits its standalone use for rare-cell studies. In addition, it offers lower per-cell gene detection sensitivity, captures only the 3′ end of transcripts, and requires custom-built microfluidics, posing technical barrier for clinical labs or multi-site studies. Drop-seq is unlikely to recover sufficient rare cells for meaningful analysis without upstream CTC enrichment. The use of external RNA controls (ERCC spike-ins), while useful for normalisation, increases sequencing costs and may introduce further noise in low-input samples [100–102].

Bead-dd-seq is a droplet-based platform with 3′ end counting and poly(A)-primed PCR amplification. It supports thousands of cells and provides unbiased gene expression profiles with relatively simple workflows. Although scalable and effective, it often requires protocol modifications to ensure robust amplicon yields for sequencing, which may limit reproducibility across labs [69].

#### Nanowell-based platform

Nanowell-based platforms, such as ICELL8 (Takara Bio) [26, 103] and BD Rhapsody single-cell analysis platforms [104] offer a compromise between throughput and control. For instance, BD Rhapsody integrates real-time imaging and barcoding with high single-cell purity (~ 80%), and its ability to reuse archived beads enhances experimental flexibility and reproducibility. It incorporates UMIs and supports targeted gene panels, offering flexibility for targeted CTC profiling. However, it shows a bias towards highly expressed housekeeping genes and uses random primer extension, which limits transcriptomic breadth unless complemented by whole-transcriptome amplification. Despite this, its high capture rate and multiplexing make it promising for low-input, targeted studies. It uses 3′-end counting and has been used in immune profiling, but has not yet been widely adopted for CTC analysis [104].

On the other hand, ICELL8 captures up to 5184 single cells and supports image-guided selection to avoid doublets. It accommodates a wide range of cell sizes (3–500 µm) and offers both 3′ and full-length chemistries. However, its singlet capture efficiency is modest (24–39%), and UMI quantification is unreliable because cDNA is amplified in the presence of barcoding primers, potentially inflating UMI counts. The alternative ICELL8 3′ DE-UMI protocol is more robust for UMI counting, since reverse transcription and cDNA amplification are uncoupled by an exonuclease digestion of barcoding primers [26, 103].

#### Microfluidic platform

Fluidigm Polaris system is one of the earliest commercial microfluidic systems designed for single-cell transcriptomics. It offers automated single-cell capture, lysis, reverse transcription, and cDNA amplification in an integrated chip-based workflow, supporting both 3′-end and full-length transcript profiling. It is well-suited for rare cell applications due to its image-based cell selection, which reduces doublet rates and allows users to exclude unwanted cell types before sequencing. However, it suffers from low throughput (typically up to 96 cells per run) and high per-cell cost. These constraints have contributed to its declining use in favour of more scalable platforms. Nonetheless, Fluidigm remains valuable in studies prioritising high data quality over quantity, especially when visual verification and full-length coverage are critical such as in characterising transcriptomic heterogeneity among CTC subpopulations or detecting splice variants [105].

#### Selecting the best approach: Amplification strategy and technical consideration

In terms of amplification, 3′ end methods—employed by 10 × Chromium [26], Drop-seq [100–102], and BD Rhapsody [104]—are cost-effective and scalable, but offer limited transcript coverage, potentially missing biologically relevant isoforms or mutation sites. Full-length approaches, as used in Smart-seq [22–24, 99] and Quartz-Seq [97, 98], deliver more comprehensive transcript data, but demand higher input quality and are more resource-intensive.

Notably, CTC analysis presents unique challenges not directly addressed by most commercial platforms: low RNA content, high contamination risk from leukocytes and the need for precise isolation of ultra-rare cells from large blood volumes. While some platforms achieve sensitivity or throughput, no single technology yet fully satisfies all requirements for CTC scRNA-seq. Therefore, platform selection should be driven by the specific goals of the study—whether that be high-resolution profiling of a few cells or population-scale discovery—and must be carefully balanced against technical limitations in terms of sensitivity, throughput and data quality.

Table 3 outlines the technical features, strengths, and limitations of each scRNA-seq technology, in order to aid researchers in making informed decisions tailored to CTC analysis and other rare-cell applications.

**Table 3:** Comparison of scRNA-seq technologies—features, strengths and limitations

Technology	Single-cell sorting	Number of cells	Capture rate	Cell barcode		UMI	cDNA coverage	Amp. method			Library prep	Strength	Limitation	Cit
Quartz-Seq/Quartz-Seq2 (Laboratory of Bioinformatics Research: open-source)	None (Requires pre-isolated single cells)	Up to 96–384 single cells per plate	~ 100%^a^	No	Quartz-Seq: No; Quartz-Seq2: Yes		Full-length		Template switching-based PCR	No; require another kit (e.g. Nextera XT)		▪ Single-tube reaction suitable for automation ▪ Quartz-Seq2: High UMI conversion efficiency and gene count detection, ▪ High sensitivity and reproducibility ▪ Suitable for large-scale transcriptome analysis and identifying rare cell populations	▪ PCR biases and amplification errors ▪ Preferential amplification of short targets (< 500bp) ▪ Higher noise level ▪ Does not perform single-cell sorting	[97, 98]
Smart-seq/Smart-seq2/Smart-seq3/Smart-seq4 (Takara Bio)	None (Requires pre-isolated single cells)	Up to 96–384 single cells per plate	~ 100%^a^	No	No		Full-length		Template switching-based PCR	No; require another kit (e.g. Nextera XT)		▪ Smart-seq: Higher sensitivity for detecting low-abundance transcripts; low dropout rate, suitable for rare single cells ▪ Smart-seq2: Require only 50 ng as starting material; mRNA sequence does not have to be known; high transcript coverage; high mappability; thermal stability of LNA-DNA base pairs ▪ Smartseq-3: No limit on cell size; no need to keep cells fresh for transport; cheaper than droplet-based methods for small sample sizes	▪ Smart-seq: low throughput, expensive per cell, amplification bias ▪ Smart-seq2: Not strand-specific; no multiplexing; transcript length bias (> 4kb); prefer high-abundance transcripts; strand-invasion bias ▪ Smart-seq3: Limited cell capacity; requires FACS sorting; labour-intensive	[22–24, 99]
Bead-dd-seq	Droplet-based microfluidic system,	100–10,000 cells	< 2%	Yes	Yes		3′ end counting		PCR-based amplification (Poly(A)-primed PCR)	Yes; require modification		▪ Sensitive and simple for single-cell library construction ▪ Unbiased characterisation of gene expression and gene regulation ▪ Unbiased profiling of diverse cell populations ▪ Scalable solution	▪ Require protocol modification to ensure sufficient amplicon yield for downstream sequencing	[69]
Drop-seq (McCarroll Lab: open-source)	Droplet-based microfluidic for single-cell encapsulation	Up to 10,000 cells	< 10%	Yes	Yes		3′ end counting		Template switching-based PCR	No; require another kit (e.g., Nextera XT)		▪ High throughput scRNA-seq ▪ Cost effective: $0.07 per cell ($653 per 10,000 cells) and fast library prep (10,000 cells per day)	▪ Requires custom microfluidics for droplet separation ▪ Lower gene-per-cell sensitivity ▪ ERCC spike-ins increase sequencing costs	[100–102]
Fluidigm Polaris system (Fluidigm)	Microfluidic-based single cell capture and processing	Up to 48 single cells per plate (capacity to scale up to 96 cells based on specific workflow)	65–80%	Yes	Yes		Full-length		Template switching-based PCR	No; require another kit (e.g., Nextera XT)		▪ Integrated workflow for single-cell captures and live-cell manipulation ▪ High precision and reproducibility ▪ Supports routine functional single-cell studies ▪ Imaging data via Visiopharm® analysis software since 2021	▪ Low capture efficiency and cell damage ▪ High cost ▪ Low throughput (only 96 cells per run)	[105]
Chromium system (10 × Genomics)	Droplet-based microfluidic for single-cell encapsulation	500–10,000 cells; > 20,000 cells for new GEM-X technology	~ 30% to ~ 80%	Yes	Yes		3′ end counting		PCR-based amplification (T7-based IVT or Poly(A)-primed PCR)	Included		▪ High throughput due to 10 × barcoding ▪ Cost effective and time saving for large-scale single-cell analysis ▪ Supports single-cell ATAC-seq ▪ Detection of rare cell types by analysing a large number of cells ▪ Capable of encapsulate single cells into droplets	▪ Only 3′ terminal fragments can be used for sequencing ▪ High number of cells requirement (not suitable for pure rare cell suspension) ▪ Low capture efficiency and high dropout rates	[26]
Clontech ICELL8 single-cell system (Takara Bio, formerly Wafergen)	Nanowell-based single cell capture and processing	Up to 5,184 single cells per nanowell chip	24–39%	Yes	Yes^b^		Full-length		Template switching-based PCR	No; require another kit (e.g., Nextera XT)		▪ Image-based cell selection for viability and singlet detection ▪ High throughput with traceable sequencing data ▪ Flexible chemistry (3′ end or full-length, SE or PE mode) ▪ Compatibility with live, fixed, and various cell sizes (3–500 µm) ▪ Removal of abnormal cells and doublets	▪ Cell limit of more than 1000 single cells ▪ Low singlet capture efficiency ▪ UMI count is unreliable as cDNA is amplified without barcode primers (use alternative ICELL8 3′ DE-UMI instead)	[26, 103]
BD Rhapsody single-cell capture and analysis system (BD Biosciences)	Nanowell-based single cell capture and processing	100–40,000 single cells per cartridge	~ 80%	Yes	Yes		3′ end counting		Random primer extension (RPE) PCR	Included		▪ Multitier molecular barcoding and multiplexing ▪ High single-cell purity (0% multiplet for ~ 1000 cells) ▪ Low inter-cell noise ▪ High capture rate ▪ Ability to visualise single-cell capture ▪ Archived beads reusable for future experiments ▪ Compatible with various downstream workflows	▪ Cell limit of more than 100 single cells ▪ Bias for housekeeping genes detection due to high expression but a targeted approach enables amplification of a selected set of genes of interest	[104]

*ATAC*-*seq* assay for transposase-accessible chromatin using sequencing, *DNA* deoxyribonucleic acid, *Drop*-*seq* droplet sequencing, *LNA* locked nucleic acid, *mRNA* messenger ribonucleic acid, *PCR* polymerase chain reaction, *PE* pair-end, *SE* single-end, *TCR* T cell receptor

^a^Since cells are manually pipetted; ^b^ when used with specific kit

### Bioinformatic analysis

After selecting an appropriate CTC enrichment method and sequencing platform, the next critical phase lies in computational analysis. This begins with the pre-processing of raw sequencing data to generate a high-quality count matrix, followed by post-processing steps such as normalisation, dimensionality reduction and clustering. Downstream analyses uncover biologically and clinically relevant insights, supporting the translational potential of CTC scRNA-seq.

To support these analyses, a wide range of computational resources have been developed, typically categorized as tools, frameworks, toolkits or pipelines. A tool refers to a single-purpose algorithm designed for a specific task (e.g. UMAP for dimensionality reduction). A framework is a broader environment that integrates multiple tools and provides built-in workflows or functions across several stages of analysis (e.g. scran). A toolkit is typically modular and customisable, allowing users to flexibly combine multiple functions, often written within the same programming ecosystem (e.g. Seurat in R). A pipeline, on the other hand, is a more rigid, end-to-end workflow (e.g. Cell Ranger) that automates a predefined series of steps from raw data to processed output.

#### Pre-processing

Pre-processing of CTC scRNA-seq data involves several key steps to convert raw sequencing files into usable expression matrices. It begins with demultiplexing (e.g. bcl2fastq), where raw BCL files are split into FASTQ files. Next, quality control and read trimming tools like FastQC [106], Trim Galore [107] or Trimmomatic [108] remove low-quality bases and adapter sequences. Species confirmation and contamination checks (e.g. FastQ Screen [109]) can be used to validate data origin. Barcode and UMI processing (e.g. alevin, zUMIs) assigns reads to individual cells. Reads are then aligned and quantified against a reference genome using tools such as STAR [110], Salmon [111] or kallisto [112]. After assigning reads to genomic features using HTSeq [113] or FeatureCounts [114], the count matrix is generated. This matrix, representing expression levels per gene per cell, forms the basis for all post-processing and downstream analyses. Integrated pipelines such as Cell Ranger can be used to streamline these steps.

#### Post-processing

Post-processing refines a raw count matrix to prepare it for meaningful biological analysis. First, doublet detection tools like Scrublet [115] or DoubletFinder [116] identify and remove droplets containing more than one cell. Normalisation (e.g. SCTransform [117, 118]) corrects for differences in sequencing depth across cells, while batch effect correction (e.g. fastMNN [119], Scanorama [120]) adjusts for technical variability between experiments. Cell cycle correction (e.g. scLVM [121]) removes gene expression noise caused by cell division stages. Imputation tools like scImpute help recover missing values due to dropouts [122]. Dimensionality reduction (e.g. PCA [123], UMAP [124, 125]) simplifies data for visualisation and pattern recognition, followed by clustering (e.g. Louvain [126], scclusteval [127]) to group similar cells. Finally, cell type annotation tools such as Monocle [128], SingleR [129] and RCA [130] assign identities to each cluster based on known markers. Comprehensive scran [131] framework integrate many of these functions.

A comprehensive summary of pre- and post-processing tools, along with their functions, computational environments and sources, is provided in Table 4.

**Table 4:** Commonly used tools for pre- and post-processing analyses of CTC scRNA-seq data

scRNA-seq data analysis tool	Environment	Source
Demultiplexing: Splitting raw sequencing data into individual samples		
bcl2fastq/bcl2fastq2	Linux/Illumina BaseSpace server	https://github.com/brwnj/bcl2fastq; https://anaconda.org/dranew/bcl2fastq; https://support.illumina.com/downloads/bcl2fastq-conversion-software-v2-20.html
Quality control, read filtering, and adapter trimming		
RSeQC [145]	Python	https://github.com/MonashBioinformaticsPlatform/RSeQC; http://rseqc.sourceforge.net/
Trimmomatic [108]	Linux	http://www.usadellab.org/cms/?page=trimmomatic; https://github.com/timflutre/trimmomatic
FastQC [106]	Java/Linux/Python	https://pypi.org/project/sequana-fastqc/; https://www.bioinformatics.babraham.ac.uk/projects/fastqc/; https://github.com/s-andrews/FastQC
Trim Galore^p^ (Cutadapt & FAstQC) [107]	Linux/Python/R	https://www.bioinformatics.babraham.ac.uk/projects/trim_galore/; https://github.com/FelixKrueger/TrimGalore; https://anaconda.org/bioconda/trim-galore
scater^f^ (edgeR, limma & monocle) [146]	R	https://github.com/jimhester/scater
SOAPnuke [147]	Linux	https://github.com/BGI-flexlab/SOAPnuke
Samtools^t^ [148]	Linux	https://github.com/samtools
MutiQC [149]	Python	https://github.com/MultiQC/MultiQC
Confirmation of species origin and contamination detection		
FastQ Screen [109]	Linux	https://www.bioinformatics.babraham.ac.uk/projects/fastq_screen; https://github.com/StevenWingett/FastQ-Screen
Barcode and UMI processing		
alevin^p^ (formerly UMI-tools; prefer droplet-based scRNA-seq) [150]	Python	https://github.com/CGATOxford/UMI-tools/blob/master/doc/Single_cell_tutorial.md
zUMIs^p^ [151]	R	https://github.com/sdparekh/zUMIs
scruff^p^ (CEL-seq/CEL-seq2) [152]	R	https://github.com/campbio/scruff
Alignment and quantification		
TopHat/TopHat2 [153, 154]	Linux	https://github.com/DaehwanKimLab/tophat; http://ccb.jhu.edu/software/tophat
Bowtie/Bowtie2 [155, 156]	Linux/Python/R	https://github.com/BenLangmead/bowtie2; https://biocontainers.pro/tools/bowtie2; https://github.com/BenLangmead/bowtie; https://pypi.org/project/bowtie/
STAR [110]	Linux/R	https://github.com/alexdobin/STAR
kallisto [112]	Linux	https://github.com/pachterlab/kallisto
Salmon [111]	Linux	https://github.com/COMBINE-lab/salmon
HISAT2 (HISAT & Bowtie2) [157]	Linux	https://github.com/DaehwanKimLab/hisat2; https://daehwankimlab.github.io/hisat2/
Assigning sequence reads		
FeatureCounts [114]	Linux	https://rnnh.github.io/bioinfo-notebook/docs/featureCounts.html
HTSeq/HTSeq 2.0 [113]	Python	https://pypi.python.org/pypi/HTSeq; https://github.com/htseq/htseq
Pipeline of pre-processing raw reads into count matrices (including demultiplexing, barcode processing, transcript mapping, feature barcode analysis)		
CellRanger^p^ (10X Genomics Chromium) [26]	Linux	https://www.10xgenomics.com/support/software/cell-ranger; https://github.com/10XGenomics/cellranger
Doublet and multiplet removal		
Scrublet[115]	Python	https://github.com/swolock/scrublet; https://anaconda.org/bioconda/scrublet
DoubletFinder [116]	R	https://github.com/chris-mcginnis-ucsf/DoubletFinder
Normalisation		
SCTransform [117, 118]	R	https://github.com/satijalab/sctransform
Linnorm [158]	R	https://github.com/kenshunyip/Linnorm; https://bioconductor.org/biocLite.R
Batch effect correction		
fastMNN [119]	R	https://bioconductor.org/packages/release/bioc/html/batchelor.html; https://github.com/satijalab/seurat-wrappers/blob/master/docs/fast_mnn.md
Scanorama [120]	Python/R	https://github.com/brianhie/scanorama
Cell cycle/growth variability correction		
scLVM/f-scLVM [121]	Python	https://github.com/PMBio/scLVM
Imputation		
scimpute [122]	R	https://github.com/Vivianstats/scImpute
Dimensionality reduction and visualisation		
PCA [123]	Python	https://github.com/erdogant/pca
t-SNE [159]	Python/R	https://github.com/shivanichander/tSNE
ZINB-WaVE [160]	R	https://github.com/drisso/zinbwave; https://bioconductor.org/packages/zinbwave
UMAP [124, 125]	Python/R	https://github.com/lmcinnes/umap
Cell clustering analysis and visualiasation		
Louvain Community Detection [126]	Python	https://github.com/taynaud/python-louvain
Leiden [161]	Python	https://github.com/vtraag/leidenalg
SC3 [162]	R	http://bioconductor.org/packages/SC3; https://github.com/hemberg-lab/SC3
Souporcell [163]	Python	https://github.com/wheaton5/souporcell
scclusteval^t^[127]	R	https://github.com/crazyhottommy/scclusteval
RISC 1.7^p^ (RPCI) [164]	R	https://github.com/bioinfoDZ/RISC
Cell-type identification		
Monocle [128]	R	https://github.com/cole-trapnell-lab/monocle-release
RCA/RCAv2 [130]	R	https://github.com/prabhakarlab/RCAv2
Harmony [165]	Python/R	https://github.com/immunogenomics/harmony
Garnett (Monocle) [166]	R	https://cole-trapnell-lab.github.io/garnett/
SingleR [129]	R	https://github.com/dviraran/SingleR
sciBET [167]	R	https://github.com/PaulingLiu/scibet
Framework for scRNA-seq analysis (including QC, normalisation, highly variable and bimodal gene identification)		
scran^f^ [131]	R	https://bioconductor.org/packages/release/bioc/html/scran.html

^p^ scRNA-seq pipeline; ^f^ framework, ^t^ toolkit

#### Downstream analysis

Downstream analysis explores biological insights after post-processing. Differential gene expression tools like Limma [132], clusterProfiler [133] and RSEM [134] are used to identify genes that vary across conditions or clusters. For biological interpretation, pathway and enrichment analysis tools (e.g. GSEA, GSVA [135], ReactomePA [136]) highlight functional pathways and gene sets involved. Cell–cell communication platforms like CellPhoneDB infer intercellular signalling [137], while copy number variation tools (inferCNV, CopyKAT) detect genomic alterations. Alternative splicing and polyadenylation are profiled using tools like rMATS [138] and Outrigger [139]. For studying developmental processes, trajectory inference tools such as Monocle3 [140] reconstruct cell lineage dynamics. Additionally, tool like scDIOR [144] and SeuratDisk enable cross-platform data conversion.

Several open-source and freely available full-featured pipelines, including Seurat [141], MAST [142] and Pagoda2 [143], integrate multiple steps of downstream analysis. Others, such as Loupe Cell Browser (10X Genomics), Strand NGS (Strand Life Sciences) and CLC Genomics Workbench (QIAGEN Digital Insights), are proprietary or accessible only through specific commercial platforms.

**Fig. 3:**
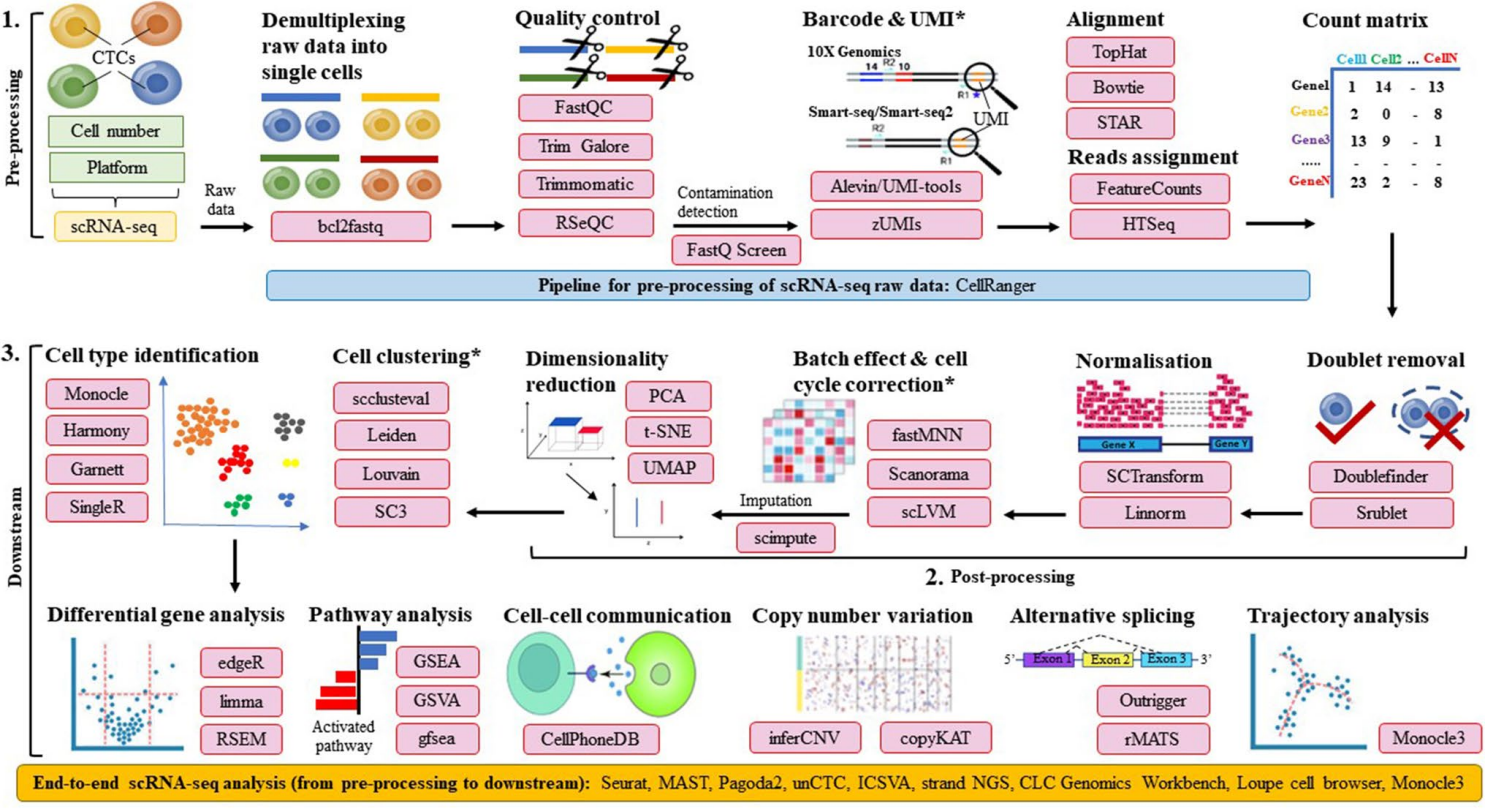
Workflow and commonly used tools in CTC scRNA-seq data analyses. 1. Pre-processing of scRNA-seq data begins with demultiplexing raw data into single CTCs (bcl2fastq), followed by quality control (FastQC, RSeQC), trimming (Trim Galore, Trimmomatic), contamination checks (FastQ Screen) and barcode/UMI processing (alevin, UMI-tools, zUMIs). Reads are aligned (TopHat, bowtie, STAR) and assigned to features (HTSeq, FeatureCounts) to generate count matrices. Cell Ranger is a commonly used pre-processing pipeline. 2. Post-processing includes double removal (doubletfinder, scrublet), normalization (SCTransform, linnorm), batch effect correction (fastMNN, scanorama, scLVM), imputation (scimpute) and dimensionality reduction (PCA, t-SNE, UMAP). 3. Downstream analysis covers clustering (scclusteval, Leiden, Louivain, SC3), cell type identification (Monocle, Harmony, Garnett, Single R), differential gene expression (edgeR, limma, RSEM) and pathway analysis (GSEA, GSVA, gsea). Other biological interpretations include cell–cell communication (CellPhone DB), copy number variation (inferCNV, copyKAT), alternative splicing (Outrigger, rMATS) and trajectory analysis (Monocle3). End-to-end scRNA-seq analysis platforms include Seurat, MAST, Pagoda2, unCTC, ICSVA, Strand NGS, CLC Genomics Workbench, Loupe Cell Browser and Monocle3. CTC-specific refinements, which address the unique characteristics of CTC data, are marked with asterisks (*)

Table 5 presents a comprehensive summary of key tools used for downstream analyses in CTC-specific scRNA-seq. It outlines the primary function of each tool along with their compatible computational environments (e.g., R, Python), and access sources (e.g., GitHub, CRAN, Bioconductor). To contextualise their use, Fig. 3 maps these tools across the entire computational pipeline—from pre-processing to downstream analysis. While general scRNA-seq workflows remain broadly applicable, CTC-specific analyses require additional considerations due to the extreme rarity and heterogeneity of these cells. Key refinements, such as barcode and UMI processing, batch effect and cell cycle correction and more nuanced clustering, are crucial for ensuring accurate interpretation. These CTC-specific refinements are marked with asterisks (*) in Fig. 3.

**Table 5:** Tools commonly used for downstream analyses of CTC scRNA-seq data

scRNA-seq data analysis tool	Environment	Source
Differential gene analysis		
edgeR v4 [168]	R	https://bioconductor.org/packages/release/bioc/html/edgeR.html; https://github.com/OliverVoogd/edgeR
Limma [132]	R	http://bioconductor.org/packages/release/bioc/html/limma.html; https://github.com/gangwug/limma
clusterProfiler [133]	R	https://github.com/YuLab-SMU/clusterProfiler
RSEM [134]	Python/R	https://github.com/deweylab/RSEM; http://deweylab.biostat.wisc.edu/rsem/
Enrichr [169]	Web server/Python	http://amp.pharm.mssm.edu/Enrichr; https://github.com/wdecoster/enrichr_cli
DESeq2 [170]	R	http://www.bioconductor.org/packages/release/bioc/html/DESeq2.html; https://github.com/thelovelab/DESeq2
WGCNA [171]	R	https://alexslemonade.github.io/refinebio-examples/04-advanced-topics/network-analysis_rnaseq_01_wgcna.html
SCDE (includes pagoda) [172, 173]	Python/R	https://github.com/hms-dbmi/scde
Metascape [174]	Web server	https://metascape.org/gp/index.html#/main/step1
GSEA and pathway analysis		
GSEA	Linux/R	https://github.com/GSEA-MSigDB
GSVA [135]	R	https://github.com/rcastelo/GSVA
KEGGProfile	R/web server	https://github.com/slzhao/KEGGprofile; https://cqs.mc.vanderbilt.edu/shiny/KEGGprofile/
Fgsea [175]	R	https://github.com/alserglab/fgsea; http://bioconductor.org/packages/devel/bioc/vignettes/fgsea/inst/doc/fgsea-tutorial.html
ReactomePA [136]	Linux/R	https://github.com/YuLab-SMU/ReactomePA
WebGestalt V1.0 [176]	Web server	https://www.webgestalt.org/
GSCA [177]	R	https://github.com/zji90/GSCA; https://zhiji.shinyapps.io/GSCA
DAVID [178]	Web server	https://david.ncifcrf.gov
Cell–cell communication		
CellPhoneDB [137]	Python	https://github.com/Teichlab/cellphonedb
Copy number variations		
inferCNV	R	https://github.com/broadinstitute/infercnv
CopyKAT	R	https://github.com/navinlabcode/copykat
Alternative splicing (AS) and alternative polyadenylation (APA) profiling		
Outrigger [139]	Python	https://github.com/YeoLab/outrigger
rMATS [138]	Linux	https://github.com/Xinglab/rmats-turbo/releases/tag/v4.3.0; http://rnaseq-mats.sourceforge.net/
Single-cell trajectories		
Monocle3^f^ [140]	R	https://github.com/cole-trapnell-lab/monocle3; http://cole-trapnell-lab.github.io/monocle3/
Pipeline/Framework/toolkit from pre-processing to downstream analysis		
MAST^f^ [142]	R	https://github.com/RGLab/MAST
Pagoda2^f^ [143]	R	https://github.com/kharchenkolab/pagoda2
ICARUS v3^f^ [179]	Web server	https://launch.icarus-scrnaseq.cloud.edu.au/app/ICARUS_v3
Seurat v5^f^ [141]	Linux/Windows/OSX/R	https://satijalab.org/seurat; https://github.com/satijalab/seurat
unCTC^p^ [39]	R	https://github.com/SaritaPoonia/unCTC
ISCVA	Web server	http://iscva.moffitt.org
Strand NGS (formerly Avadis NGS)^t^	Commercial tool	http://www.strand-ngs.com/
CLC Genomics Workbench^t^	Commercial tool	https://digitalinsights.qiagen.com/products-overview/discovery-insights-portfolio/analysis-and-visualization/qiagen-clc-genomics-workbench/
Loupe Cell Browser v5.0.0 (10X Genomics)	Commercial tool/Linux	https://www.10xgenomics.com/support/software/loupe-browser/latest/getting-started/lb-what-is-loupe-browser
Data transformation between different platforms		
SeuratDisk	R	https://mojaveazure.github.io/seurat-disk/articles/convert-anndata.html
scDIOR[144]	Python/R	https://github.com/JiekaiLab/scDIOR

^p^ scRNA-seq pipeline; ^f^ framework, ^t^ toolkit

## Emerging research frontiers

### CTC-related new/novel rare cells

Recent advances in single-cell RNA sequencing have expanded the scope of CTC research, revealing novel subtypes, interactions and functional states that were previously inaccessible or unrecognised. Several new CTC populations were discovered in 2022 and 2025. In 2022, a preclinical model using patient-derived cerebrospinal fluid (CSF)-CTCs in melanoma uncovered IGF1R as a potential therapeutic target, with consistent gene expression profiles across *in vitro* and *in vivo* expansions [180]. TERT^+^, PSMA-high CTCs were identified in prostate cancer, enriched for genes associated with proliferation and metastasis, and showing distinct gene expression patterns between metastatic and local cases [181].

CTC–platelet adhesion complexes were identified in 2025, highlighting CD155 upregulation in immune evasion via TIGIT interaction, regulated through the FAK/JNK/c-Jun pathway [182]. Another study discovered CTCs with increased genomic content (CTC-IGCs), exhibiting polyploidisation and clonal relationships with typical CTCs, suggesting new dimensions of CTC biology [183]. Lastly, two distinct CTC populations, namely platelet-positive and platelet-negative, have been characterized, with differential expression of MYC targets and stemness markers. This has led to new insights into CTC-mediated metastasis [184].

### Rare cells

Other studies have uncovered important rare cell populations via scRNA-seq. For instance, Yang et al*.* developed a tumour self-seeding (TSC) mouse model using CRC (HCT116) and HCC (PLC/PRF/5) cell lines and identified a TM4SF1^+^ TSC population with a metastatic profile in liver and CRC patients from two scRNA-seq datasets [185]. In the same year, a common ‘starter’ subpopulation driving tumour initiation and metastasis was identified through evolutionary trajectory analysis, revealing upregulated cell cycle-related genes crucial for neuroblastoma progression and observing a partial EMT transition along the metastatic route to bone marrow [186].

In NSCLC, patient-derived CTCs were used to create xenograft models, which identified a regenerative alveolar epithelial type II-like cell population shared with non-xenografted NSCLC metastases. Single-cell transcriptomic analysis has uncovered distinct drug responses and cellular heterogeneity within the CTC-derived xenografts tumours validated against patient metastases tissues [187]. Similarly, Zheng and the co-researchers successfully isolated circulating tumour-initiating cells (CTICs) from HCC patients and confirmed molecular heterogeneity among individual CTICs via scRNA-seq profiling. They further characterised these subsets into distinct phenotypes (Zheng et al., 2022). Another scRNA-seq study of pancreatic cancer demonstrated the efficacy of adjuvant HGF/c-Met inhibition, providing the first confirmation of circulating human pancreatic stellate cells and accurately distinguishing human from mouse cells using UMI counts, orthologous gene expression and canonical markers [189].

### Hybrid cells

Concurrently, hybrid tumour cells have been implicated in metastasis, though their molecular landscape and detection remain unclear [190]. Nevertheless, the high sensitivity of scRNA-seq permits the identification and profiling of these hybrid cells. For instance, Anderson and the co-researchers identified neoplastic-immune hybrid cells in uveal melanoma, which exhibited properties such as enhanced cell motility, immune evasion and altered metabolism, with ligand-receptor pathways potentially driving metastasis [191]. In CRC and BC, tumour hybrid cells co-expressed epithelial and macrophage markers (*EpCAM* and *KRT8*), with distinct transcriptomic profiles were reported to be linked to tumour progression [192].

In prostate cancer bone metastasis, scRNA-seq analysis identified a unique cancer cell cluster with myeloid cell markers, suggesting that cell fusion between disseminated tumour cells and bone marrow cells could be a potential source of myeloid-like hybrid cells. Multi-omics analysis revealed that hybrid cells displayed an enhanced EMT phenotype, increased tumorigenicity, and resistance to docetaxel and ferroptosis, although they remained sensitive to radiotherapy [193]. Conversely, hybrid cells from primary melanoma expressing melanoma antigens correlated with poorer responses to immune checkpoint blockade [194].

In another attempt, Menyailo et al*.* applied scRNA-seq to profile CD45-negative and CD45-positive circulating epithelial cells (CECs) in non-mBC patients. These CECs comprise distinct populations of both aneuploid and diploid cells, as demonstrated by their transcriptional profiles. Notably, cancer-associated signalling pathways were abundant in only one aneuploid CD45^–^ CEC population, possibly representing an aggressive subset of CTC. Therefore, CD45^–^ and CD45^+^ CECs are highly heterogeneous and include aneuploid cells, which are most likely circulating tumour and hybrid cells, respectively and diploid cells [195]. In kidney cancer, scRNA-seq of c-Kit^+^ and DBA^+^ cells identified four groups: progenitor cells (PCs), immune cells (ICs), hybrid cells and non-classified cells. Hybrid cells expressed both PC and IC markers or Aqp2 [196]. Therefore, investigating hybrid cells through scRNA-seq shows promise for advancing cancer biology research, and could potentially revolutionise clinical cancer management.

**Fig. 4:**
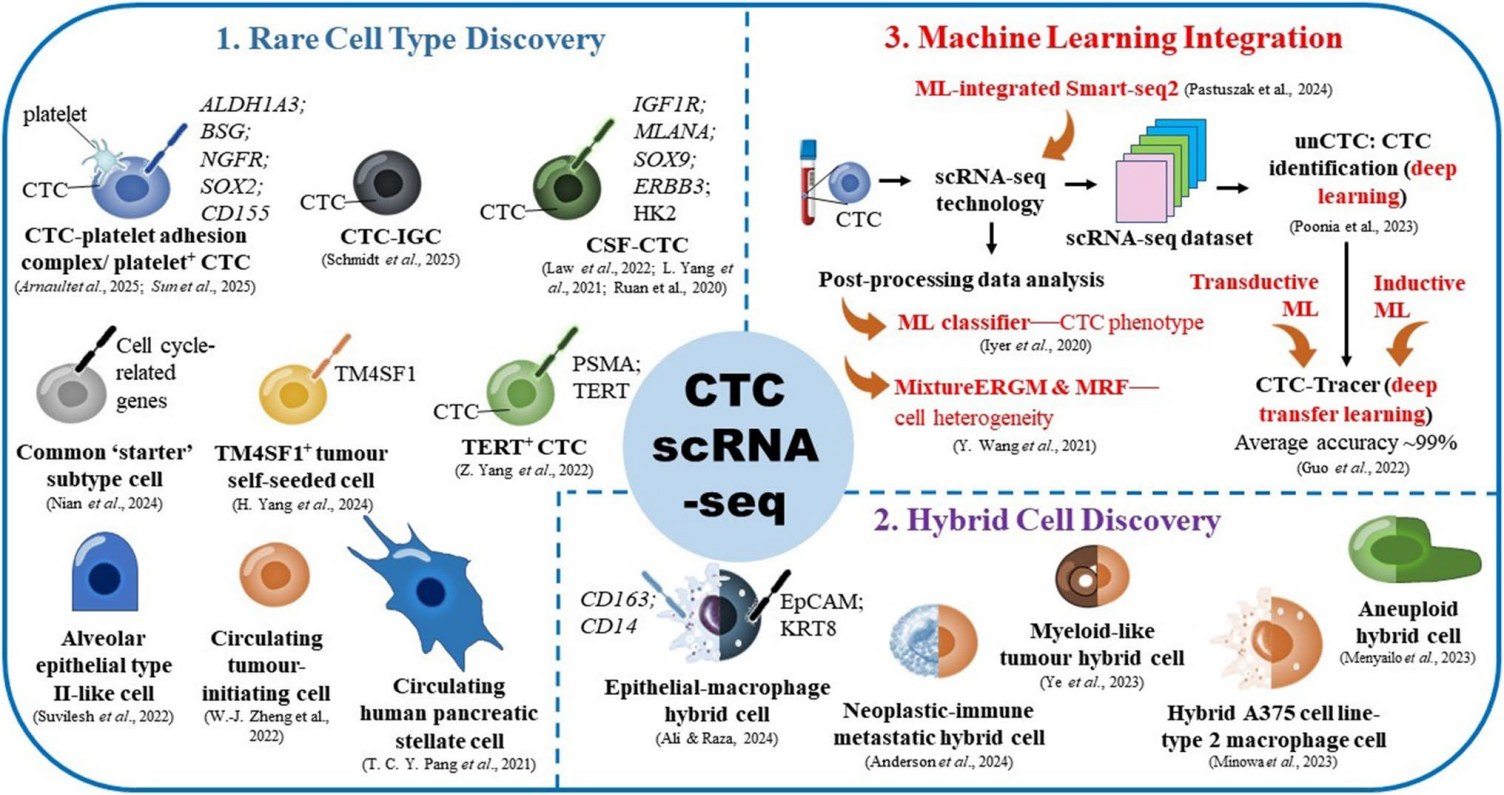
Emerging research frontiers in CTC scRNA-seq: rare and hybrid cells, and machine learning-integrated CTC analyses. 1. Rare cell type discovery identifies rare and CTC-related rare subpopulations with unique transcriptomes that may evade standard enrichment methods. 2. Hybrid cell discovery enables detection of fusion between tumour and immune cells that drive tumour plasticity, immune evasion and metastasis. 3. Integration of machine learning enhances CTC classification, uncover hidden cell states, predict treatment response and improve cell-type annotation accuracy

Table 6 summarises key studies related to rare cells, CTC-related and hybrid subpopulations, highlighting the CTC enrichment methods, scRNA-seq platforms, number of cells analysed, and cancer type. In parallel, Fig. 4 visually maps these rare and hybrid populations, their associated surface markers, and how ML has been integrated into CTC scRNA-seq analyses to enhance cell classification and functional insight.

**Table 6:** Emerging research frontiers in rare CTC subtypes and hybrid cells

Research frontier	Type of cancer	Enrichment method	scRNA-seq technology	Number of cells analysed	Finding	Cit
CTC-related rare cells	Lung cancer	ChimeraX-i120 platform	Smart-seq2 (Takara Bio)	**CTC-platelet adhesion complexes**	▪ Identification of **CTC-platelet adhesion complexes** via scRNA-seq ▪ Detection of *CD155* upregulation, with functional assays confirming its role in immune evasion via TIGIT interaction, suggesting FAK/JNK/c-Jun cascade as a regulatory pathway	[182]
	Prostate cancer	Micro-manipulation of fluorescence-stained cells (CK^+^, VIM^+^, CD45/CD31^−^)	Modified Smart-Seq2 (Takara Bio)	Matched bone marrow and peripheral blood samples from 31 advanced prostate cancer patients (NCT01505868) *(number not stated)*	▪ Identification of CTCs with increased genomic content (**CTC-IGC**), with nuclear diameter at least twice the average CTC size, detected in 9.7% of peripheral blood samples and 80.6% of bone marrow samples ▪ Single cell copy number profiling of **CTC-IGC** revealed clonal origin with typical CTCs, suggesting complete polyploidisation	[183]
	Skin cancer	Ficoll-paque density gradient separation, followed by negative enrichment (EaspSep direct human CTC enrichment kit)	Smart-seq2 (Takara Bio)	scRNA-seq dataset GSE255299: 75 CTCs from seven SKCM patients [83]; scRNA-seq dataset GSE72056: 4645 cells from 19 metastases of SKCM [197]	▪ Comparison of CTC (GSE255299) and solid metastases (GSE72056) revealed upregulation of 11 platelet activation genes (*THBS1, PF4, TIMP3, VWF, ITGB3, F2, COMP, GP1BA, PLEK, RAPGEF3*, and *GNB2*) ▪ *F3* and *SERPINE1* were expressed at lower levels in CTCs, with no significant change in *PLAT* expression ▪ UMAP analysis identified two CTC clusters (60% vs. 40%), corresponding to **Platelet-Positive** and Platelet-Negative **CTCs** ▪ **Platelet-Positive CTCs** showed enrichment in MYC TARGETS v1 and increased expression of stemness markers (*ALDH1A3, BSG, NGFR, SOX2*)	[184]
	Skin cancer	Separation by centrifugation, followed by adapting CellSearch (Janssen Diagnostics) via the CELLTRACKS circulating melanoma cell kit	Chromium system (10 × Genomics)	148 CTCs from patient-9; 149 CTCs from patient-12; non-cultured **CSF-CTCs** from patient-8, 10, and 11 (*number not stated*)	▪ Development of a preclinical model of patient-derived **CSF-CTCs** for experimental therapeutics in melanoma with LMD ▪ scRNA-seq revealed *IGF1R* as a potential therapeutic target in melanoma LMD, along with *MLANA, SOX9*, and *ERBB3* ▪ Despite heterogeneity between patients, 96 to 97.7% of genes were retained after *in vitro* and *in vivo* expansion, confirming that CTCs resembled original patient samples	[180]
	Prostate cancer	TERT-based CTC detection, followed by imaging flow cytometry (CD45^−^, PSMA^+^); GSE67980: Dynabeads MyOne Streptavidin T1 (Invitrogen), followed by microfluidic CTC-iChip (EpCAM^+^, CDH11^+^, CD45^−^)	GSE67980: Modified ABI SOLiD protocol	GSE67980: 77 prostate CTCs [60]	▪ Identification of **telomerase (TERT) positive CTCs** with PSMA high expression associated with prostate cancer metastasis ▪ The mean ‘**TERT**^**+**^** CTCs**’ number was 6.11 ± 9.63 in the metastatic group and 4.09 ± 3.41 in the local group ▪ scRNA-seq of 77 prostate CTCs showed enrichment of proliferation-related terms and high metastasis-related gene expression in PSMA-high CTCs	[181]
Rare cells	Liver and colorectal cancer	GSE132257: tumour dissociation kit protocol (Miltenyi Biotec) [198]; GSE125449: MACS (Miltenyi Biotech) [199]	Chromium system (10 × Genomics)	GSE132257: 18,409 CRC cells; GSE125449: 5,115 liver cancer cells	▪ Development of tumour self-seeding mouse model using CRC (HCT116) and HCC (PLC/PRF/5) cell lines ▪ Identification of **TM4SF1**^**+**^** tumour self-seeded (TSC) population** with a metastatic profile in liver and CRC patients from two scRNAseq datasets	[185]
	Neuroblastoma	Magnetic-activated cell sorting *via* tumour cell isolation kit (Miltenyi Biotec) (CD45^−^, DAPI^−^)	Chromium system (10 × Genomics)	15,447 neuroblastoma cells from eight NB samples, including paired samples of primary tumours and bone marrow metastases	▪ Identification of a **common ‘starter’ subpopulation** driving tumour initiation and metastasis through evolutionary trajectory analysis ▪ Discovery of upregulated cell cycle-related genes in the ‘starter’ subpopulation, crucial for neuroblastoma progression ▪ Observation of partial epithelial-to-mesenchymal transition along the metastatic route to bone marrow	[186]
	Lung cancer	GSE123904: Tissue dissociation *via* Gentle MACS Octo Dissociator and filtering [200]; snRNA-seq: Immunostaining (pan-CK^+^, CD45^–^), followed by single nuclei isolation	Chromium system (10 × Genomics)	GSE123904: 40,505 single cells from 17 freshly resected human tissue samples, including normal lung (*n* = 4), primary LUAD (7 untreated, 1 post-chemotherapy), and LUAD metastases (brain *n* = 3, bone *n* = 1, adrenal *n* = 1)	▪ Development of NSCLC CDX mouse models with ptPDX‑derived CTCs ▪ Identification of an additional regenerative **alveolar epithelial type II (AT2)-like cell** population in CDX tumours that was also identified in non-xenografted NSCLC patients’ metastases tissues ▪ Identification of distinct drug responses and cell heterogeneities in CDX tumours that can be validated in NSCLC metastases tissues	[187]
	Liver cancer	Integrated four channel immunomagnetic-microfluidic platform (iMAC) (EpCAM^+^, Cd133^+^, CD90^+^, CD24^+^)	NA	**CTICs** from blood samples of 33 HCC patients (*number not stated*)	▪ Identification of distinct phenotypes in four **CTICs** subsets ▪ Distinguishing primary HCC, recurrent HCC, and TACE-resistant HCC *via* distinct stem-related markers’ expression of **CTICs** ▪ Development of a novel and informative method for accurate **CTICs** detection and characterisation	[188]
	Pancreas cancer	Negative immunomagnetic AutoMACS Pro cell separator and Mouse Cell Depletion Microbead cocktail kit (Miltenyi Biotec)	Chromium system (10 × Genomics)	Primary and metastatic tumour cells from mice; CTCs from blood; cultured pancreatic cancer-associated and AsPC-1 cells (*number not stated*)	▪ Demonstration of the efficacy of adjuvant HGF/c-Met inhibition for pancreatic cancer and first confirmation of the existence of **circulating human pancreatic stellate cells** ▪ Accurate identification and classification of **circulating human pancreatic stellate cells** and pancreas cancer cells, distinguishing them from mouse cells using UMI counts, orthologous gene expression and canonical markers	[189]
	Lung cancer	FACS to obtain single cell suspensions	Smart-seq2 (Takara Bio)	1776 candidate CTCs from five LUAD-LM patients	▪ Definition of **CSF-CTCs** *via* epithelial markers (*EPCAM, CDH1, KRT7, KRT8, KRT18, MUC1*), proliferation markers (*CCND1, TOP2A*) and genes with lung origin (*SFTPA1, SFTPA2, SFTPB, NAPSA*) ▪ Identification of metastatic-CTC signature genes crucial for the survival and metastasis (*CEACAM6*) ▪ Quantification of the degree of heterogeneity ▪ Identification of biomarkers during the progression of a liver metastases patient with cancer of unknown primary site	[46]
Hybrid cells	Eye cancer	GSE139829: Slightly modified Miltenyi tumour dissociation kit protocol [201, 202]	GSE139829: Chromium system (10 × Genomics)	GSE139829: 59,915 single cells from eight primary and three metastatic tumours	▪ Identification of **neoplastic-immune hybrid cells with metastatic properties** in cyclic immunofluorescence images and uveal melanoma scRNA-seq dataset ▪ Identification of **hybrid cells** properties: enhanced cell motility and cytoskeleton rearrangement, immune evasion, and altered metabolism ▪ Identification of potential drivers of metastasis in **hybrid cells** including ligand-receptor pathways related to IGF1-IGFR1, GAS6-AXL, LGALS9-P4HB, APP-CD74 and CXCL12-CXCR4	[191]
	Colorectal and breast cancer	GSE178341: tissue processing, followed by CD45 enrichment; BC dataset: human tumour dissociation kit (Miltenyi Biotec)	GSE178341: Chromium system (10 × Genomics) BC dataset: Chromium system (10 × Genomics)	GSE178341: 257,251 tumour cells and 112,861 adjacent normal cells from 64 tumours of 62 patients and normal tissue from 36 CRC patients[203]; BC dataset: 100,064 cells (*GSE number not stated*) [204]	▪ Identification of **tumour hybrid cells co-expressing epithelial** (*EpCAM* and *KRT8*) and **macrophage markers** (*CD163* and *CD14*) and expressing a distinct transcriptome in CRC and BC *via* single-cell and spatial transcriptomics ▪ Establishment of a framework for **hybrid cell** identification in large datasets ▪ Rare hybrid cells were present in normal healthy tissue but had a distinct gene expression profile from **tumour hybrid cells**, which showed features linked to tumour progression	[192]
	Prostate cancer	Flow cytometry	Chromium system (10 × Genomics)	GSE143791: solid metastatic tissue, liquid bone marrow at the site of the metastasis and liquid bone marrow from a vertebral body distant from the tumour site (Distal) (*number not stated*) [205]; 17,602 **tumour hybrid cells** from the bone metastasis model	▪ scRNAseq GSE143791 dataset identified a unique cancer cell cluster in PCa bone metastases with myeloid cell marker, linked to immune regulation and tumour progression pathway ▪ Development of a bone metastasis model through caudal artery injection of tumour cells and sorted the **tumour hybrid cells** by flow cytometry ▪ Detection of cell fusion between disseminated tumour cells and bone marrow cells as a potential source of **myeloid-like hybrid cells** ▪ Multi-omics analysis revealed cell adhesion and proliferation pathways in **hybrid cells** ▪ scRNAseq and CyTOF showed tumour-associated neutrophils/monocytes/macrophages enriched in **hybrid cells**-induced immunosuppressive microenvironment ▪ **Hybrid** cells exhibited an enhanced EMT phenotype, higher tumorigenicity, resistance to docetaxel and ferroptosis, but sensitive to radiotherapy	[193]
	Skin cancer	*Not stated*	*Not stated*	*Not stated*	▪ Establishment of **hybrid clones of A375 cells and type 2 macrophages** in co-culture ▪ scRNA-seq dataset identified **hybrid cells** in primary melanoma, with macrophages expressing melanoma antigens (melan A, tyrosinase, premelanosome protein), and their presence correlated with poorer response to immune checkpoint blockade	[194]
	Breast cancer	RosettSep negative selection	Chromium system (10 × Genomics)	13,741 CD45-negative and CD45-positive CECs from 20 BC patients and one healthy donor	▪ Identification of 16 cell clusters ▪ Identification and profiling of CD45-negative and CD45-positive CECs ▪ Identification of **aneuploid** CTCs and **hybrid cells** *via* analysing DNA ploidy	[195]
	Kidney cancer	Centrifugation, followed by FACS (DBA^+^ and c-Kit^+^) and C1™ Single-Cell Auto Prep System (Fluidigm)	Smart-seq (Takara Bio)	Four runs: 66 cells derived from a 1:1 mixture of c-Kit^+^ and DBA^+^ populations; 74 cells from the same mixture; 43 c-Kit^+^ cells; and 58 c-Kit^+^ cells	▪ Identification of four groups individually: o Group 1: Genes associated with PCs o Group 2: Genes associated with ICs o Group 3: Genes associated with both PCs and ICs (**hybrid cells**) o Group 4: Do not contain markers for either cell type ▪ Combination of four scRNAseq datasets: o Group 1: 74 PCs o Group 2: 87 A-ICs o Group 3: 23 B-ICs ▪ Identification of **hybrid IC/PCs** expressing either the IC-marker transcripts or Aqp2	[196]

*A*-*ICs* type A intercalated cells, *AsPC*-*1* luciferase-tagged human pancreatic cancer cells, *B*-*ICs* type B intercalated cells, *CD45* lymphocyte common antigen, *CDX* CTC-derived xenografts, *CECs* circulating epithelial cells, *CR* conditional reprogramming, *CSF* cerebrospinal fluid, *CTCs* circulating tumour cells, *CTICs* circulating tumour-initiating cells, *EpCAM* epithelial cell adhesion molecule, *F3* tissue factor, *HCC* hepatocellular carcinoma, *IC* intercalated cells, *ISCVA* interactive single cell visual analytics, *KRT* keratin, *MEL* melanoma, *NM* normal mammary, *PBMC* peripheral blood mononuclear cell, *PCa* prostate cancer, *PC* principal cell, *ptPDX* primary tumour patient-derived xenograft, *RNA* ribonucleic acid, *scRNA*-*seq* single-cell RNA-sequencing, *Smart*-*seq* switch mechanism at the 5′ end of RNA template sequencing, *snRNA*-*seq* single nuclear RNA sequencing, *TIGIT* T cell immunoreceptor with immunoglobulin and ITIM domain, *UMI* unique molecular identifiers, *VIM* vimentin

### Artificial intelligence integration

With the increasing application of scRNA-seq in larger-scale studies, the challenge of clustering and analysing the vast amounts of data generated has become a major bottleneck. To address this, ML techniques have been leveraged to streamline data analysis and improve the identification of rare cell populations including CTCs and hybrid cells. For example, Iyer et al*.* employed ML techniques on publicly available single-cell expression profiles, enabling the detection of various CTCs across different cancer types and opening avenues for discovering consistent pan-cancer CTC surface proteins beyond the widely used epithelial cell adhesion molecule (EpCAM) [20]. Building on this concept, Pastuszak et al*.* developed ML-based classifiers using four tree-based models trained and tested on Smart-Seq2 data from primary tumour sections of BC patients and PBMCs, along with a public dataset featuring annotated CTC expression profiles from 34 mBC patients, including those with triple-negative BC. Their top-performing models achieved approximately 95% balanced accuracy on the CTC test set per cell, accurately identifying 133 out of 138 CTCs and CTC-PBMC clusters [21].

Beyond CTC identification, ML has been applied to tackle the complexity of cellular heterogeneity. Wang et al. introduced a mixture exponential graph and Markov random field model for cell heterogeneity analysis. This method helps overcome the high-dimensional challenges of scRNA-seq data by identifying key marker genes related to cancer and the immune system, aiding in the discovery of potential therapeutic targets [206]. In the context of hybrid cells, Thong et al*.* combined scRNA-seq with ML-based dataset alignment to explore phenotypic similarities between mammary stem cells and BC cells. They revealed that normal mammary stem cells exhibit epithelial, mesenchymal, and hybrid epithelial/mesenchymal states—linking hybrid phenotypes with stemness and tumour progression [207].

Deep learning approaches have further advanced rare cell analysis. For example, unCTC, an R package developed by Poonia et al., provides an unbiased framework for identifying and characterizing CTCs using deep dictionary learning, clustering and expression-based copy number variation inference [39]. In parallel, Guo et al. introduced CTC-Tracer, a deep transfer learning-based algorithm that corrects for distributional shifts between primary cancer cells and CTCs, allowing accurate lesion label transfer across diverse RNA-seq datasets [208].

Altogether, the integration of AI and scRNA-seq is opening new frontiers in rare cell analysis, offering unprecedented opportunities for characterizing CTCs and tumour hybrid cells. Future research directions should focus on developing AI models for real-time, automated interpretation of molecular and phenotypic features. Such advancements hold the potential to transform clinical oncology by delivering rapid, actionable insights for personalized treatment decisions, ultimately improving patient outcomes and accelerating cancer research [209].

Table 7 outlines emerging research frontiers in scRNA-seq, with a focus on AI-driven approaches for CTC identification and hybrid cell characterisation.

**Table 7:** Emerging research frontiers in machine learning integration in CTC scRNA-seq

Research frontier	Type of cancer	Number of cells analysed	Finding	Cit
Hybrid cells and machine learning	Breast cancer	200 random cells per sample from three normal mammary NM-CR cell pairs	▪ Quantified cell state distributions and identification of **hybrid** epithelial/mesenchymal states (*KRT14*, *KRT18*, *VIM*, and *EPCAM)* ▪ Analysis of human/mouse scRNA-seq data from mammary glands, incorporating bulk TCGA tumour data, other studies (NM, CR, Bach, Nguyen) and referencing the Giraddi Mouse Mammary Transcriptome Atlas (all *via* **ML**)	[207]
Machine learning	Breast cancer	GSE109761: 262 CTCs, 14 CTC-PBMC clusters and 82 PBMCs from 34 metastatic breast cancer patients [53]; GSE118389: 1534 TNBC cells; Single Cell Expression Atlas: 27,620 PBMCs from 6 healthy donors [210]	▪ Detection of CTCs *via* **ML** using Smart‑Seq2 sequencing ▪ Analysis of two feature selection methods and **four ML algorithms**: Extreme Gradient Boosting, Light Gradient Boosting Machine, Random Forest and Balanced Random Forest ▪ Validation on real CTC data achieved nearly 96% balanced accuracy, outperforming EpCAM-based classification for identifying CTCs in metastatic breast cancer patients	[21]
	Pancreatic and breast cancer	GSE51372: 75 CTC-enriched blood cells, 12 fibroblast cells, 16 pancreatic cancer cells, 12 white blood cells, 18 GFP-traced CTCs, 20 GFP-traced primary tumour cells, and 34 RNA dilutions from pancreatic tumours [25]; GSE118389: 1534 cells from six fresh TNBC tumours [210]	▪ Introduction of a **mixture exponential graph** and **Markov random field model** for cell heterogeneity analysis and overcoming high-dimensional challenges ▪ Identification of hub nodes in cell–cell networks and incorporation of Rank Product for robust differential gene expression analysis ▪ Identification of seven key marker genes in GSE118389, which play important roles in the immune system and are closely linked to generic cancer genes	[206]
	Breast, prostate, skin, lung, and pancreas cancer	558 single CTCs from 10 different studies (6 breast studies and 1 each for other cancer types) [211]	▪ Design and training of an **ML classifier** to identify CTC phenotypes based on their gene expression profiles ▪ Validation of the **ML classifier** *via* breast CTCs captured using a newly developed microfluidic system for label-free enrichment of CTCs	[20]
Machine learning and deep transfer learning	Breast cancer	72 CTCs from 6 patients of three major subtypes: ER^−^/PR^−^/HER2^−^, ER^+^/PR^+^/HER2^−^ and ER^−^/PR^−^/HER2^+^	▪ Development of unCTC, an R package for unbiased identification and characterisation of CTCs from scRNA-seq ▪ unCTC includes a novel method of scRNA-seq clustering, named **deep dictionary learning** using k-means clustering cost, expression-based copy number variation inference, and combinatorial, marker-based verification of the malignant phenotypes	[39]
	Skin, liver, breast, and prostate cancer	Primary dataset: 372 CTCs four cancer CTC scRNA-seq datasets (CNP0000095, GSE109761 [53], GSE67980 [60], GSE157745 [36]) with two blood datasets (400 PBMCs and 800 blood cells from 32 immunophenotypic cell types); validation dataset: 451 CTCs from MEL and BRCA cancers (GSE75367, PRJNA471754, GSE51827 [74], GSE38495 [22]	▪ Design of CTC-Tracer, a **deep transfer learning**-based algorithm with an average accuracy of ~ 99% (on test samples) ▪ Integration of two **ML** modes: **transductive and inductive learning** ▪ CTC-tracer improves CTC analysis by: o Correcting for data differences between primary tumours and CTCs o Transferring tumour origin information from large cancer cell atlases to CTCs	[208]

*CTCs* circulating tumour cells, *EpCAM* epithelial cell adhesion molecule, *ER* estrogen receptor, *GFP* green fluorescent protein, *HER2* human epidermal growth factor receptor 2, *KRT* keratin, *ML* machine learning, *PBMC* peripheral blood mononuclear cell, *PR* progesterone receptor, *scRNA*-*seq* single-cell RNA-sequencing, *TCGA* The Cancer Genome Atlas, *TNBC* triple-negative breast cancer, *VIM* vimentin

## Conclusion

ScRNA-seq is a powerful tool for profiling rare cell populations like CTCs and hybrid cells, revealing transcriptomic diversity, novel subtypes, and therapeutic targets. It facilitates patient re-stratification and advances metastasis research. Therefore, we propose a comprehensive workflow for CTC scRNA-seq, covering enrichment, single-cell sorting, sequencing, and data pre-processing. By integrating ML, data analysis and interpretability are enhanced, while focusing on hybrid cells could provide new insights into cancer immunobiology. Researchers should follow this proposed workflow to standardise approaches and enhance the utility of scRNA-seq in precision oncology.

## References

[1] Qi, W., Tian, L., Xu, J., Li, Z., & Wang, T. (2025). Multimodal framework in lung cancer management: Integrating liquid biopsy with traditional diagnostic techniques. *Cancer Management And Research,**17*, 461–481. 10.2147/CMAR.S506630 40060704 10.2147/CMAR.S506630 PMC11889406

[2] Wang, P., Yang, R. K., Jelloul, F. Z., Luthra, R., Routbort, M. J., Chen, H., Loghavi, S., Ok, C. Y., Kanagal-Shamanna, R., Roy-Chowdhuri, S., Medeiros, L. J., & Patel, K. P. (2025). Routine clinical liquid biopsy testing for solid tumors delivers the promise of minimally invasive detection of genomic variants with a faster turnaround time. *JCO Precision Oncology*. 10.1200/PO.24.00299 40138600 10.1200/PO.24.00299

[3] Liang, X., Tang, Q., Chen, J., Wei, Y. (2025). Liquid biopsy: A breakthrough technology in early cancer screening. Cancer Screening and Prevention 0 0–0. 10.14218/CSP.2024.00031.

[4] Shegekar, T., Vodithala, S., & Juganavar, A. (2023). The emerging role of liquid biopsies in revolutionising cancer diagnosis and therapy. *Cureus,**15*, Article e43650. 10.7759/cureus.43650 37719630 10.7759/cureus.43650 PMC10505053

[5] Pandey, S., & Yadav, P. (2025). Liquid biopsy in cancer management: Integrating diagnostics and clinical applications. *Practical Laboratory Medicine,**43*, Article e00446. 10.1016/j.plabm.2024.e00446 39839814 10.1016/j.plabm.2024.e00446 PMC11743551

[6] Song, J., Ye, X., & Xiao, H. (2025). Liquid biopsy entering clinical practice: Past discoveries, current insights, and future innovations. *Critical Reviews in Oncology/Hematology,**207*, Article 104613. 10.1016/j.critrevonc.2025.104613 39756526 10.1016/j.critrevonc.2025.104613

[7] Castro-Giner, F., & Aceto, N. (2020). Tracking cancer progression: From circulating tumor cells to metastasis. *Genome Medicine,**12*, Article 31. 10.1186/s13073-020-00728-3 32192534 10.1186/s13073-020-00728-3 PMC7082968

[8] Krog, B. L., & Henry, M. D. (2018). Biomechanics of the circulating tumor cell microenvironment. *Advances in Experimental Medicine and Biology,**1092*, 209–233. 10.1007/978-3-319-95294-9_11 30368755 10.1007/978-3-319-95294-9_11 PMC7304329

[9] Lowes, L. E., & Allan, A. L. (2018). Circulating tumor cells and implications of the epithelial-to-mesenchymal transition. *Advances in Clinical Chemistry,**83*, 121–181. 10.1016/bs.acc.2017.10.004 29304900 10.1016/bs.acc.2017.10.004

[10] Rossi, T., Gallerani, G., Martinelli, G., Maltoni, R., & Fabbri, F. (2021). Circulating tumor cells as a tool to untangle the breast cancer heterogeneity issue. *Biomedicines,**9*, Article 1242. 10.3390/biomedicines9091242 34572427 10.3390/biomedicines9091242 PMC8466266

[11] Yu, T., Wang, C., Xie, M., Zhu, C., Shu, Y., Tang, J., & Guan, X. (2021). Heterogeneity of CTC contributes to the organotropism of breast cancer. *Biomedicine & Pharmacotherapy,**137*, Article 111314. 10.1016/j.biopha.2021.111314 33581649 10.1016/j.biopha.2021.111314

[12] Whalen, R. M., Anderson, A. N., Jones, J. A., Sims, Z., Chang, Y. H., Nederlof, M. A., Wong, M. H., & Gibbs, S. L. (2024). Ultra high content analyses of circulating and tumor associated hybrid cells reveal phenotypic heterogeneity. *Scientific Reports,**14*, Article 7350. 10.1038/s41598-024-57381-8 38538742 10.1038/s41598-024-57381-8 PMC10973471

[13] Gauck, D., Keil, S., Niggemann, B., Zänker, K. S., & Dittmar, T. (2017). Hybrid clone cells derived from human breast epithelial cells and human breast cancer cells exhibit properties of cancer stem/initiating cells. *BMC Cancer,**17*, Article 515. 10.1186/s12885-017-3509-9 28768501 10.1186/s12885-017-3509-9 PMC5541689

[14] Hass, R., von der Ohe, J., & Dittmar, T. (2021). Hybrid formation and fusion of cancer cells in vitro and in vivo. *Cancers,**13*(17), Article 4496. 10.3390/cancers13174496 34503305 10.3390/cancers13174496 PMC8431460

[15] Orrapin, S., Thongkumkoon, P., Udomruk, S., Moonmuang, S., Sutthitthasakul, S., Yongpitakwattana, P., Pruksakorn, D., & Chaiyawat, P. (2023). Deciphering the biology of circulating tumor cells through single-cell RNA sequencing: Implications for precision medicine in cancer. *International Journal of Molecular Sciences,**24*, Article 12337. 10.3390/ijms241512337 37569711 10.3390/ijms241512337 PMC10418766

[16] Tieng, F. Y. F., Baharudin, R., Abu, N., Mohd Yunos, R.-I., Lee, L.-H., & Ab Mutalib, N.-S. (2020). Single cell transcriptome in colorectal cancer—current updates on its application in metastasis, chemoresistance and the roles of circulating tumor cells. *Frontiers in Pharmacology,**11*, Article 135. 10.3389/fphar.2020.00135 32174835 10.3389/fphar.2020.00135 PMC7056698

[17] Armingol, E., Officer, A., Harismendy, O., & Lewis, N. E. (2021). Deciphering cell–cell interactions and communication from gene expression. *Nature Reviews Genetics,**22*, 71–88. 10.1038/s41576-020-00292-x 33168968 10.1038/s41576-020-00292-x PMC7649713

[18] Longo, S. K., Guo, M. G., Ji, A. L., & Khavari, P. A. (2021). Integrating single-cell and spatial transcriptomics to elucidate intercellular tissue dynamics. *Nature Reviews Genetics,**22*, 627–644. 10.1038/s41576-021-00370-8 34145435 10.1038/s41576-021-00370-8 PMC9888017

[19] Meng, G., Tang, W., Huang, E., Li, Z., & Feng, H. (2023). A comprehensive assessment of cell type-specific differential expression methods in bulk data. *Briefings in Bioinformatics,**24*, Article bbac516. 10.1093/bib/bbac516 36472568 10.1093/bib/bbac516 PMC9851321

[20] Iyer, A., Gupta, K., Sharma, S., Hari, K., Lee, Y. F., Ramalingam, N., Yap, Y. S., West, J., Bhagat, A. A., Subramani, B. V., Sabuwala, B., Tan, T. Z., Thiery, J. P., Jolly, M. K., Ramalingam, N., & Sengupta, D. (2020). Integrative analysis and machine learning based characterization of single circulating tumor cells. *Journal of Clinical Medicine,**9*, Article 1206. 10.3390/jcm9041206 32331451 10.3390/jcm9041206 PMC7230872

[21] Pastuszak, K., Sieczczyński, M., Dzięgielewska, M., Wolniak, R., Drewnowska, A., Korpal, M., Zembrzuska, L., Supernat, A., & Żaczek, A. J. (2024). Detection of circulating tumor cells by means of machine learning using Smart-Seq2 sequencing. *Scientific Reports,**14*, Article 11057. 10.1038/s41598-024-61378-8 38744942 10.1038/s41598-024-61378-8 PMC11094170

[22] Ramsköld, D., Luo, S., Wang, Y.-C., Li, R., Deng, Q., Faridani, O. R., Daniels, G. A., Khrebtukova, I., Loring, J. F., Laurent, L. C., Schroth, G. P., & Sandberg, R. (2012). Full-length mRNA-seq from single cell levels of RNA and individual circulating tumor cells. *Nature Biotechnology,**30*, 777–782. 10.1038/nbt.2282 22820318 10.1038/nbt.2282 PMC3467340

[23] Picelli, S., Björklund, Å. K., Faridani, O. R., Sagasser, S., Winberg, G., & Sandberg, R. (2013). Smart-seq2 for sensitive full-length transcriptome profiling in single cells. *Nature Methods,**10*, 1096–1098. 10.1038/nmeth.2639 24056875 10.1038/nmeth.2639

[24] Picelli, S., Faridani, O. R., Björklund, Å. K., Winberg, G., Sagasser, S., & Sandberg, R. (2014). Full-length RNA-seq from single cells using Smart-seq2. *Nature Protocols,**9*, 171–181. 10.1038/nprot.2014.006 24385147 10.1038/nprot.2014.006

[25] Ting, D. T., Wittner, B. S., Ligorio, M., Jordan, N. V., Shah, A. M., Miyamoto, D. T., Aceto, N., Bersani, F., Brannigan, B. W., Xega, K., Ciciliano, J. C., Zhu, H., MacKenzie, O. C., Trautwein, J., Arora, K. S., Shahid, M., Ellis, H. L., Qu, N., Bardeesy, N., … Haber, D. A. (2014). Single-cell RNA sequencing identifies extracellular matrix gene expression by pancreatic circulating tumor cells. *Cell Reports,**8*, 1905–1918. 10.1016/j.celrep.2014.08.029 25242334 10.1016/j.celrep.2014.08.029 PMC4230325

[26] Zheng, G. X. Y., Terry, J. M., Belgrader, P., Ryvkin, P., Bent, Z. W., Wilson, R., Ziraldo, S. B., Wheeler, T. D., McDermott, G. P., Zhu, J., Gregory, M. T., Shuga, J., Montesclaros, L., Underwood, J. G., Masquelier, D. A., Nishimura, S. Y., Schnall-Levin, M., Wyatt, P. W., Hindson, C. M., … Bielas, J. H. (2017). Massively parallel digital transcriptional profiling of single cells. *Nature Communications,**8*, Article 14049. 10.1038/ncomms14049 28091601 10.1038/ncomms14049 PMC5241818

[27] D’Avola, D., Villacorta-Martin, C., Martins-Filho, S. N., Craig, A., Labgaa, I., von Felden, J., Kimaada, A., Bonaccorso, A., Tabrizian, P., Hartmann, B. M., Sebra, R., Schwartz, M., & Villanueva, A. (2018). High-density single cell mRNA sequencing to characterize circulating tumor cells in hepatocellular carcinoma. *Scientific Reports,**8*, Article 11570. 10.1038/s41598-018-30047-y 30068984 10.1038/s41598-018-30047-y PMC6070499

[28] Cheng, Y.-H., Chen, Y.-C., Lin, E., Brien, R., Jung, S., Chen, Y.-T., Lee, W., Hao, Z., Sahoo, S., Kang, H. M., Cong, J., Burness, M., Nagrath, S., Wicha, M. S., & Yoon, E. (2019). Hydro-seq enables contamination-free high-throughput single-cell RNA-sequencing for circulating tumor cells. *Nature Communications,**10*, Article 2163. 10.1038/s41467-019-10122-2 31092822 10.1038/s41467-019-10122-2 PMC6520360

[29] Kojima, M., Harada, T., Fukazawa, T., Kurihara, S., Saeki, I., Takahashi, S., & Hiyama, E. (2021). Single-cell DNA and RNA sequencing of circulating tumor cells. *Scientific Reports,**11*, Article 22864. 10.1038/s41598-021-02165-7 34819539 10.1038/s41598-021-02165-7 PMC8613180

[30] Shi, F., Jia, F., Wei, Z., Ma, Y., Fang, Z., Zhang, W., & Hu, Z. (2021). A microfluidic chip for efficient circulating tumor cells enrichment, screening, and single-cell RNA sequencing. *Proteomics,**21*, Article e2000060. 10.1002/pmic.202000060 33219587 10.1002/pmic.202000060

[31] Dong, Z., Wang, Y., Xu, G., Liu, B., Wang, Y., Reboud, J., Jajesniak, P., Yan, S., Ma, P., Liu, F., Zhou, Y., Jin, Z., Yang, K., Huang, Z., Zhuo, M., Jia, B., Fang, J., Zhang, P., Wu, N., & Chang, L. (2024). Genetic and phenotypic profiling of single living circulating tumor cells from patients with microfluidics. *Proceedings of the National Academy of Sciences of the United States of America,**121*, Article e2315168121. 10.1073/pnas.2315168121 38683997 10.1073/pnas.2315168121 PMC11087790

[32] Vasantharajan, S.S. (2024). Characterisation of circulating tumour cells isolated by a size-based method and identification of circulating tumour cell-specific transcriptomic signatures to understand colorectal cancer metastasis, University of Otago. https://ourarchive.otago.ac.nz/esploro/outputs/doctoral/Characterisation-of-circulating-tumour-cells-isolated/9926522380201891 (Accessed November 29, 2024).

[33] Stucky, A., Viet, C. T., Aouizerat, B. E., Ye, Y., Doan, C., Mundluru, T., Sedhiazadeh, P., Sinha, U. K., Chen, X., Zhang, X., Li, S. C., Cai, J., & Zhong, J. F. (2024). Single-cell molecular profiling of head and neck squamous cell carcinoma reveals five dysregulated signaling pathways associated with circulating tumor cells. *Cancer Control,**31*, 10732748241251572. 10.1177/10732748241251571 38869038 10.1177/10732748241251571 PMC11179551

[34] Moeyersoms, A. H. M., Knechtel, K. W., Rong, A. J., Gallo, R. A., Zhang, M., Marsh, H. M., Sargi, Z. B., Leibowitz, J. M., Civantos, F. J., Weed, D. T., Dubovy, S. R., Tse, D. T., & Pelaez, D. (2024). Circulating adenoid cystic carcinoma associated MYB transcripts enable rapid and sensitive detection of metastatic disease in blood liquid biopsies. *The Journal of Liquid Biopsy*. 10.1016/j.jlb.2024.100276 39801676 10.1016/j.jlb.2024.100276 PMC11725320

[35] Wu, L., Wang, Z., Zia, A., Kelley, S. O., & de Perrot, M. (2025). Mesothelioma cell heterogeneity identified by single cell RNA sequencing. *Scientific Reports,**15*, 8725. 10.1038/s41598-025-92542-3 40082554 10.1038/s41598-025-92542-3 PMC11906801

[36] Hong, X., Roh, W., Sullivan, R. J., Wong, K. H. K., Wittner, B. S., Guo, H., Dubash, T. D., Sade-Feldman, M., Wesley, B., Horwitz, E., Boland, G. M., Marvin, D. L., Bonesteel, T., Lu, C., Aguet, F., Burr, R., Freeman, S. S., Parida, L., Calhoun, K., & Haber, D. A. (2021). The lipogenic regulator SREBF2 induces Transferrin in circulating melanoma cells and suppresses ferroptosis. *Cancer Discovery,**11*, 678–695. 10.1158/2159-8290.CD-19-1500 33203734 10.1158/2159-8290.CD-19-1500 PMC7933049

[37] Stewart, C. A., Gay, C. M., Xi, Y., Sivajothi, S., Sivakamasundari, V., Fujimoto, J., Bolisetty, M., Hartsfield, P. M., Balasubramaniyan, V., Chalishazar, M. D., Moran, C., Kalhor, N., Stewart, J., Tran, H., Swisher, S. G., Roth, J. A., Zhang, J., de Groot, J., Glisson, B., … Byers, L. A. (2020). Single-cell analyses reveal increased intratumoral heterogeneity after the onset of therapy resistance in small-cell lung cancer. *Nature Cancer,**1*, 423–436. 10.1038/s43018-019-0020-z 33521652 10.1038/s43018-019-0020-z PMC7842382

[38] Grigoryeva, E., Tashireva, L., Alifanov, V., Savelieva, O., Zavyalova, M., Menyailo, M., Khozyainova, A., Denisov, E. V., Bragina, O., Popova, N., Cherdyntseva, N. V., & Perelmuter, V. (2024). Integrin-associated transcriptional characteristics of circulating tumor cells in breast cancer patients. *PeerJ,**12*, Article e16678. 10.7717/peerj.16678 38250718 10.7717/peerj.16678 PMC10800097

[39] Poonia, S., Goel, A., Chawla, S., Bhattacharya, N., Rai, P., Lee, Y. F., Yap, Y. S., West, J., Bhagat, A. A., Tayal, J., Mehta, A., Ahuja, G., Majumdar, A., Ramalingam, N., & Sengupta, D. (2023). Marker-free characterization of full-length transcriptomes of single live circulating tumor cells. *Genome Research,**33*, 80–95. 10.1101/gr.276600.122 36414416 10.1101/gr.276600.122 PMC9977151

[40] Rieckmann, L.-M., Spohn, M., Ruff, L., Agorku, D., Becker, L., Borchers, A., Krause, J., O’Reilly, R., Hille, J., Velthaus-Rusik, J.-L., Beumer, N., Günther, A., Willnow, L., Imbusch, C. D., Iglauer, P., Simon, R., Franzenburg, S., Winter, H., Thomas, M., … Janning, M. (2024). Diagnostic leukapheresis reveals distinct phenotypes of NSCLC circulating tumor cells. *Molecular Cancer,**23*, Article 93. 10.1186/s12943-024-01984-2 38720314 10.1186/s12943-024-01984-2 PMC11077784

[41] Kojima, M., Harada, T., Fukazawa, T., Kurihara, S., Touge, R., Saeki, I., Takahashi, S., & Hiyama, E. (2023). Single-cell next-generation sequencing of circulating tumor cells in patients with neuroblastoma. *Cancer Science,**114*, 1616–1624. 10.1111/cas.15707 36571449 10.1111/cas.15707 PMC10067419

[42] Kozuka, M., Battaglin, F., Jayachandran, P., Wang, J., Arai, H., Soni, S., Zhang, W., Hirai, M., Matsusaka, S., & Lenz, H.-J. (2021). Clinical significance of circulating tumor cell induced epithelial-mesenchymal transition in patients with metastatic colorectal cancer by single-cell RNA-sequencing. *Cancers,**13*, Article 4862. 10.3390/cancers13194862 34638346 10.3390/cancers13194862 PMC8507666

[43] Zolotovskaia, M. A., Sorokin, M. I., Petrov, I. V., Poddubskaya, E. V., Moiseev, A. A., Sekacheva, M. I., Borisov, N. M., Tkachev, V. S., Garazha, A. V., Kaprin, A. D., Shegay, P. V., Giese, A., Kim, E., Roumiantsev, S. A., & Buzdin, A. A. (2020). Disparity between inter-patient molecular heterogeneity and repertoires of target drugs used for different types of cancer in clinical oncology. *International Journal of Molecular Sciences,**21*, 1580. 10.3390/ijms21051580 32111026 10.3390/ijms21051580 PMC7084891

[44] Hamelin, B., Obradović, M. M. S., Sethi, A., Kloc, M., Münst, S., Beisel, C., Eschbach, K., Kohler, H., Soysal, S., Vetter, M., Weber, W. P., Stadler, M. B., & Bentires-Alj, M. (2023). Single-cell analysis reveals inter- and intratumour heterogeneity in metastatic breast cancer. *Journal of Mammary Gland Biology and Neoplasia,**28*, 26. 10.1007/s10911-023-09551-z 38066300 10.1007/s10911-023-09551-z PMC10709262

[45] Goldman, S. L., MacKay, M., Afshinnekoo, E., Melnick, A. M., Wu, S., Mason, C.E. (2019). The impact of heterogeneity on single-cell sequencing, Frontiers in Genetics 10. https://www.frontiersin.org/articles/10.3389/fgene.2019.00008 (Accessed January 9, 2024). 10.3389/fgene.2019.00008 PMC6405636 30881372

[46] Ruan, H., Zhou, Y., Shen, J., Zhai, Y., Xu, Y., Pi, L., Huang, R., Chen, K., Li, X., Ma, W., Wu, Z., Deng, X., Wang, X., Zhang, C., & Guan, M. (2020). Circulating tumor cell characterization of lung cancer brain metastases in the cerebrospinal fluid through single-cell transcriptome analysis. *Clinical and Translational Medicine,**10*, Article e246. 10.1002/ctm2.246 33377642 10.1002/ctm2.246 PMC7737787

[47] Brechbuhl, H. M., Vinod-Paul, K., Gillen, A. E., Kopin, E. G., Gibney, K., Elias, A. D., Hayashi, M., Sartorius, C. A., & Kabos, P. (2020). Analysis of circulating breast cancer cell heterogeneity and interactions with peripheral blood mononuclear cells. *Molecular Carcinogenesis,**59*, 1129–1139. 10.1002/mc.23242 32822091 10.1002/mc.23242 PMC7895311

[48] Pauken, C. M., Kenney, S. R., Brayer, K. J., Guo, Y., Brown-Glaberman, U. A., & Marchetti, D. (2021). Heterogeneity of circulating tumor cell neoplastic subpopulations outlined by single-cell transcriptomics. *Cancers,**13*, Article 4885. 10.3390/cancers13194885 34638368 10.3390/cancers13194885 PMC8508335

[49] Arnoletti, J. P., Reza, J., Rosales, A., Monreal, A., Fanaian, N., Whisner, S., Srivastava, M., Rivera-Otero, J., Yu, G., Phanstiel, O., IV., Altomare, D. A., Tran, Q., & Litherland, S. A. (2022). Pancreatic ductal adenocarcinoma (PDAC) circulating tumor cells influence myeloid cell differentiation to support their survival and immunoresistance in portal vein circulation. *PLoS One,**17*, e0265725. 10.1371/journal.pone.0265725 35316296 10.1371/journal.pone.0265725 PMC8939813

[50] Mishra, A., Huang, S.-B., Dubash, T., Burr, R., Edd, J. F., Wittner, B. S., Cunneely, Q. E., Putaturo, V. R., Deshpande, A., Antmen, E., Gopinathan, K. A., Otani, K., Miyazawa, Y., Kwak, J. E., Guay, S. Y., Kelly, J., Walsh, J., Nieman, L. T., Galler, I., … Toner, M. (2025). Tumor cell-based liquid biopsy using high-throughput microfluidic enrichment of entire leukapheresis product. *Nature Communications,**16*, Article 32. 10.1038/s41467-024-55140-x 39746954 10.1038/s41467-024-55140-x PMC11696112

[51] Arneth, B. (2019). Tumor microenvironment. *Medicina (Kaunas),**56*, Article 15. 10.3390/medicina56010015 31906017 10.3390/medicina56010015 PMC7023392

[52] Tieng, F. Y. F., Lee, L.-H., Ab Mutalib, N.-S. (2022). Deciphering colorectal cancer immune microenvironment transcriptional landscape on single cell resolution—A role for immunotherapy, Frontiers in Immunology 13. https://www.frontiersin.org/articles/10.3389/fimmu.2022.959705 (Accessed September 5, 2022). 10.3389/fimmu.2022.959705 PMC9399368 36032085

[53] Szczerba, B. M., Castro-Giner, F., Vetter, M., Krol, I., Gkountela, S., Landin, J., Scheidmann, M. C., Donato, C., Scherrer, R., Singer, J., Beisel, C., Kurzeder, C., Heinzelmann-Schwarz, V., Rochlitz, C., Weber, W. P., Beerenwinkel, N., & Aceto, N. (2019). Neutrophils escort circulating tumour cells to enable cell cycle progression. *Nature,**566*, 553–557. 10.1038/s41586-019-0915-y 30728496 10.1038/s41586-019-0915-y

[54] Sun, Y.-F., Wu, L., Liu, S.-P., Jiang, M.-M., Hu, B., Zhou, K.-Q., Guo, W., Xu, Y., Zhong, Y., Zhou, X.-R., Zhang, Z.-F., Liu, G., Liu, S., Shi, Y.-H., Ji, Y., Du, M., Li, N.-N., Li, G.-B., Zhao, Z.-K., … Yang, X.-R. (2021). Dissecting spatial heterogeneity and the immune-evasion mechanism of CTCs by single-cell RNA-seq in hepatocellular carcinoma. *Nature Communications,**12*, Article 4091. 10.1038/s41467-021-24386-0 34215748 10.1038/s41467-021-24386-0 PMC8253833

[55] Liu, X., Song, J., Zhang, H., Liu, X., Zuo, F., Zhao, Y., Zhao, Y., Yin, X., Guo, X., Wu, X., Zhang, H., Xu, J., Hu, J., Jing, J., Ma, X., & Shi, H. (2023). Immune checkpoint HLA-E: CD94-NKG2A mediates evasion of circulating tumor cells from NK cell surveillance. *Cancer Cell,**41*, 272-287.e9. 10.1016/j.ccell.2023.01.001 36706761 10.1016/j.ccell.2023.01.001

[56] Tang, H.-D., Wang, Y., Xie, P., Tan, S.-Y., Li, H.-F., Shen, H., Zhang, Z., Lei, Z.-Q., & Zhou, J.-H. (2022). The crosstalk between immune infiltration, circulating tumor cells, and metastasis in pancreatic cancer: Identification of HMGB3 from a multiple omics analysis. *Frontiers in Genetics,**13*, Article 892177. 10.3389/fgene.2022.892177 35754798 10.3389/fgene.2022.892177 PMC9213737

[57] Celià-Terrassa, T., & Kang, Y. (2024). How important is EMT for cancer metastasis? *PLoS Biology,**22*, Article e3002487. 10.1371/journal.pbio.3002487 38324529 10.1371/journal.pbio.3002487 PMC10849258

[58] Negishi, R., Yamakawa, H., Kobayashi, T., Horikawa, M., Shimoyama, T., Koizumi, F., Sawada, T., Oboki, K., Omuro, Y., Funasaka, C., Kageyama, A., Kanemasa, Y., Tanaka, T., Matsunaga, T., & Yoshino, T. (2022). Transcriptomic profiling of single circulating tumor cells provides insight into human metastatic gastric cancer. *Communications Biology,**5*, 20. 10.1038/s42003-021-02937-x 35017627 10.1038/s42003-021-02937-x PMC8752828

[59] Pang, S., Xu, S., Wang, L., Wu, H., Chu, Y., Ma, X., Li, Y., Zou, B., Wang, S., & Zhou, G. (2023). Molecular profiles of single circulating tumor cells from early breast cancer patients with different lymph node statuses. *Thoracic Cancer,**14*, 156–167. 10.1111/1759-7714.14728 36408679 10.1111/1759-7714.14728 PMC9834698

[60] Miyamoto, D. T., Zheng, Y., Wittner, B. S., Lee, R. J., Zhu, H., Broderick, K. T., Desai, R., Fox, D. B., Brannigan, B. W., Trautwein, J., Arora, K. S., Desai, N., Dahl, D. M., Sequist, L. V., Smith, M. R., Kapur, R., Wu, C.-L., Shioda, T., Ramaswamy, S., … Haber, D. A. (2015). RNA-seq of single prostate CTCs implicates noncanonical Wnt signaling in antiandrogen resistance. *Science,**349*, 1351–1356. 10.1126/science.aab0917 26383955 10.1126/science.aab0917 PMC4872391

[61] Wu, Q., Gu, Z., Shang, B., Wan, D., Zhang, Q., Zhang, X., Xie, P., Cheng, S., Zhang, W., & Zhang, K. (2024). Circulating tumor cell clustering modulates RNA splicing and polyadenylation to facilitate metastasis. *Cancer Letters,**588*, Article 216757. 10.1016/j.canlet.2024.216757 38417668 10.1016/j.canlet.2024.216757

[62] Sundaresan, T. K., Dubash, T. D., Zheng, Z., Bardia, A., Wittner, B. S., Aceto, N., Silva, E. J., Fox, D. B., Liebers, M., Kapur, R., Iafrate, J., Toner, M., Maheswaran, S., & Haber, D. A. (2021). Evaluation of endocrine resistance using Esr1 genotyping of circulating tumor cells and plasma Dna. *Breast Cancer Research and Treatment,**188*, 43–52. 10.1007/s10549-021-06270-z 34101078 10.1007/s10549-021-06270-z PMC8667563

[63] Capuozzo, M., Ferrara, F., Santorsola, M., Zovi, A., & Ottaiano, A. (2023). Circulating tumor cells as predictive and prognostic biomarkers in solid tumors. *Cells,**12*, 2590. 10.3390/cells12222590 37998325 10.3390/cells12222590 PMC10670669

[64] Li, X., Sun, H., Liu, Q., Liu, Y., Hou, Y., & Jin, W. (2021). Conjoint analysis of circulating tumor cells and solid tumors for exploring potential prognostic markers and constructing a robust novel predictive signature for breast cancer. *Cancer Cell International,**21*, 708. 10.1186/s12935-021-02415-8 34953500 10.1186/s12935-021-02415-8 PMC8710246

[65] Yang, L., Yan, X., Chen, J., Zhan, Q., Hua, Y., Xu, S., Li, Z., Wang, Z., Dong, Y., Zuo, D., Xue, M., Tang, Y., Herschman, H. R., Lu, S., Shi, Q., & Wei, W. (2021). Hexokinase 2 discerns a novel circulating tumor cell population associated with poor prognosis in lung cancer patients. *Proceedings of the National Academy of Sciences of the United States of America,**118*, Article e2012228118. 10.1073/pnas.2012228118 33836566 10.1073/pnas.2012228118 PMC7980452

[66] Chen, V. L., Huang, Q., Harouaka, R., Du, Y., Lok, A. S., Parikh, N. D., Garmire, L. X., & Wicha, M. S. (2022). A dual-filtration system for single-cell sequencing of circulating tumor cells and clusters in HCC. *Hepatology Communications,**6*, 1482–1491. 10.1002/hep4.1900 35068084 10.1002/hep4.1900 PMC9134808

[67] Abramova, A., Rivandi, M., Yang, L., Stamm, N., Cieslik, J.-P., Honisch, E., Niederacher, D., Fehm, T., Neubauer, H., & Franken, A. (2023). A workflow for the enrichment, the identification, and the isolation of non-apoptotic single circulating tumor cells for RNA sequencing analysis. *Cytometry Part A,**105*, 242–251. 10.1002/cyto.a.24816 10.1002/cyto.a.24816 38054742

[68] Ebright, R. Y., Lee, S., Wittner, B. S., Niederhoffer, K. L., Nicholson, B. T., Bardia, A., Truesdell, S., Wiley, D. F., Wesley, B., Li, S., Mai, A., Aceto, N., Vincent-Jordan, N., Szabolcs, A., Chirn, B., Kreuzer, J., Comaills, V., Kalinich, M., Haas, W., … Micalizzi, D. S. (2020). Deregulation of ribosomal protein expression and translation promotes breast cancer metastasis. *Science,**367*, 1468–1473. 10.1126/science.aay0939 32029688 10.1126/science.aay0939 PMC7307008

[69] Matsunaga, H., Goto, M., Arikawa, K., Shirai, M., Tsunoda, H., Huang, H., & Kambara, H. (2015). A highly sensitive and accurate gene expression analysis by sequencing (“bead-seq”) for a single cell. *Analytical Biochemistry,**471*, 9–16. 10.1016/j.ab.2014.10.011 25449304 10.1016/j.ab.2014.10.011

[70] Huh, H. D., Sub, Y., Oh, J., Kim, Y. E., Lee, J. Y., Kim, H.-R., Lee, S., Lee, H., Pak, S., Amos, S. E., Vahala, D., Park, J. H., Shin, J. E., Park, S. Y., Kim, H. S., Roh, Y. H., Lee, H.-W., Guan, K.-L., Choi, Y. S., … Park, H. W. (2023). Reprogramming anchorage dependency by adherent-to-suspension transition promotes metastatic dissemination. *Molecular Cancer,**22*, Article 63. 10.1186/s12943-023-01753-7 36991428 10.1186/s12943-023-01753-7 PMC10061822

[71] Huh, H. D., Sub, Y., Oh, J., Kim, Y. E., Lee, J. Y., Kim, H., Lee, S., Lee, H., Pak, S., Amos, S. E., Vahala, D., Park, J. H., Shin, J. E., Park, S. Y., Kim, H. S., Roh, Y. H., Lee, H., Guan, K., Choi, Y. S., … Park, H. W. (2023). Reprogramming anchorage dependency by adherent-to-suspension transition promotes metastatic dissemination. *Molecular Cancer,**22*, 135. 10.1186/s12943-023-01838-3 36991428 10.1186/s12943-023-01753-7 PMC10061822

[72] Gkountela, S., Castro-Giner, F., Szczerba, B. M., Vetter, M., Landin, J., Scherrer, R., Krol, I., Scheidmann, M. C., Beisel, C., Stirnimann, C. U., Kurzeder, C., Heinzelmann-Schwarz, V., Rochlitz, C., Weber, W. P., & Aceto, N. (2019). Circulating tumor cell clustering shapes DNA methylation to enable metastasis seeding. *Cell,**176*, 98-112.e14. 10.1016/j.cell.2018.11.046 30633912 10.1016/j.cell.2018.11.046 PMC6363966

[73] Aceto, N., Bardia, A., Wittner, B. S., Donaldson, M. C., O’Keefe, R., Engstrom, A., Bersani, F., Zheng, Y., Comaills, V., Niederhoffer, K., Zhu, H., Mackenzie, O., Shioda, T., Sgroi, D., Kapur, R., Ting, D. T., Moy, B., Ramaswamy, S., Toner, M., … Maheswaran, S. (2018). AR expression in breast cancer CTCs associates with bone metastases. *Molecular Cancer Research,**16*, 720–727. 10.1158/1541-7786.MCR-17-0480 29453314 10.1158/1541-7786.MCR-17-0480 PMC5882540

[74] Aceto, N., Bardia, A., Miyamoto, D. T., Donaldson, M. C., Wittner, B. S., Spencer, J. A., Yu, M., Pely, A., Engstrom, A., Zhu, H., Brannigan, B. W., Kapur, R., Stott, S. L., Shioda, T., Ramaswamy, S., Ting, D. T., Lin, C. P., Toner, M., Haber, D. A., & Maheswaran, S. (2014). Circulating tumor cell clusters are oligoclonal precursors of breast cancer metastasis. *Cell,**158*, 1110–1122. 10.1016/j.cell.2014.07.013 25171411 10.1016/j.cell.2014.07.013 PMC4149753

[75] Liu, X., Song, J., Liu, X., Zhang, H., Wang, X., Li, Y., Yang, Z., Jing, J., Ma, X., & Shi, H. (2023). Protocol for identifying immune checkpoint on circulating tumor cells of human pancreatic ductal adenocarcinoma by single-cell RNA sequencing. *Star Protocols,**4*, Article 102539. 10.1016/j.xpro.2023.102539 37659082 10.1016/j.xpro.2023.102539 PMC10491853

[76] Dimitrov-Markov, S., Perales-Patón, J., Bockorny, B., Dopazo, A., Muñoz, M., Baños, N., Bonilla, V., Menendez, C., Duran, Y., Huang, L., Perea, S., Muthuswamy, S. K., Al-Shahrour, F., Lopez-Casas, P. P., & Hidalgo, M. (2020). Discovery of new targets to control metastasis in pancreatic cancer by single-cell transcriptomics analysis of circulating tumor cells. *Molecular Cancer Therapeutics,**19*, 1751–1760. 10.1158/1535-7163.MCT-19-1166 32499301 10.1158/1535-7163.MCT-19-1166

[77] Franses, J. W., Philipp, J., Missios, P., Bhan, I., Liu, A., Yashaswini, C., Tai, E., Zhu, H., Ligorio, M., Nicholson, B., Tassoni, E. M., Desai, N., Kulkarni, A. S., Szabolcs, A., Hong, T. S., Liss, A. S., Fernandez-del Castillo, C., Ryan, D. P., Maheswaran, S., … Ting, D. T. (2020). Pancreatic circulating tumor cell profiling identifies LIN28B as a metastasis driver and drug target. *Nature communications,**11*, 3303. 10.1038/s41467-020-17150-3 32620742 10.1038/s41467-020-17150-3 PMC7335061

[78] Tang, F., Barbacioru, C., Nordman, E., Xu, N., Bashkirov, V. I., Lao, K., & Surani, M. A. (2010). RNA-seq analysis to capture the transcriptome landscape of a single cell. *Nature Protocols,**5*, Article 10.1038/nprot.2009.236. 10.1038/nprot.2009.236 10.1038/nprot.2009.236 PMC3847604 20203668

[79] Zhu, L., Kan, K.-J., Grün, J. L., Hissa, B., Yang, C., Győrffy, B., Loges, S., Reißfelder, C., & Schölch, S. (2020). GAS2L1 is a potential biomarker of circulating tumor cells in pancreatic cancer. *Cancers,**12*, Article E3774. 10.3390/cancers12123774 10.3390/cancers12123774 PMC7765300 33333841

[80] Chen, M., Li, H., Xu, X., Bao, X., Xue, L., Ai, X., Xu, J., Xu, M., Shi, Y., Zhen, T., Li, J., Yang, Y., Ji, Y., Fu, Z., Xing, K., Qing, T., Wang, Q., Zhong, P., & Zhu, S. (2023). Identification of RAC1 in promoting brain metastasis of lung adenocarcinoma using single-cell transcriptome sequencing. *Cell Death & Disease,**14*, Article 330. 10.1038/s41419-023-05823-y 37202394 10.1038/s41419-023-05823-y PMC10195834

[81] Vasantharajan, S. S., Barnett, E., Gray, E. S., McCall, J. L., Rodger, E. J., Eccles, M. R., Munro, F., Pattison, S., & Chatterjee, A. (2022). Assessment of a size-based method for enriching circulating tumour cells in colorectal cancer. *Cancers,**14*, Article 3446. 10.3390/cancers14143446 35884509 10.3390/cancers14143446 PMC9319975

[82] Zheng, X., Song, J., Yu, C., Zhou, Z., Liu, X., Yu, J., Xu, G., Yang, J., He, X., Bai, X., Luo, Y., Bao, Y., Li, H., Yang, L., Xu, M., Song, N., Su, X., Xu, J., Ma, X., & Shi, H. (2022). Single-cell transcriptomic profiling unravels the adenoma-initiation role of protein tyrosine kinases during colorectal tumorigenesis. *Signal Transduction and Targeted Therapy,**7*, 1–14. 10.1038/s41392-022-00881-8 35221332 10.1038/s41392-022-00881-8 PMC8882672

[83] Fankhauser, R., Chang, M., Garrison, Z., Berryman, R., Lucero, O. M., Fuiten, A., DePatie, N., Seifert, H., & Kulkarni, R. P. (2022). Single-cell identification of melanoma biomarkers in circulating tumor cells. *Cancers,**14*, Article 4921. 10.3390/cancers14194921 36230844 10.3390/cancers14194921 PMC9564060

[84] Sade-Feldman, M., Yizhak, K., Bjorgaard, S. L., Ray, J. P., de Boer, C. G., Jenkins, R. W., Lieb, D. J., Chen, J. H., Frederick, D. T., Barzily-Rokni, M., Freeman, S. S., Reuben, A., Hoover, P. J., Villani, A.-C., Ivanova, E., Portell, A., Lizotte, P. H., Aref, A. R., Eliane, J.-P., … Hacohen, N. (2018). Defining T cell states associated with response to checkpoint immunotherapy in melanoma. *Cell,**175*, 998-1013.e20. 10.1016/j.cell.2018.10.038 30388456 10.1016/j.cell.2018.10.038 PMC6641984

[85] Wang, Z., Ahmed, S., Labib, M., Wang, H., Wu, L., Bavaghar-Zaeimi, F., Shokri, N., Blanco, S., Karim, S., Czarnecka-Kujawa, K., Sargent, E. H., McGray, A. J. R., de Perrot, M., & Kelley, S. O. (2023). Isolation of tumour-reactive lymphocytes from peripheral blood via microfluidic immunomagnetic cell sorting. *Nature Biomedical Engineering,**7*, 1188–1203. 10.1038/s41551-023-01023-3 37037966 10.1038/s41551-023-01023-3

[86] Green, D., Eyre, H., Singh, A., Taylor, J. T., Chu, J., Jeys, L., Sumathi, V., Coonar, A., Rassl, D., Babur, M., Forster, D., Alzabin, S., Ponthan, F., McMahon, A., Bigger, B., Reekie, T., Kassiou, M., Williams, K., Dalmay, T., … Finegan, K. G. (2020). Targeting the MAPK7/MMP9 axis for metastasis in primary bone cancer. *Oncogene,**39*, 5553–5569. 10.1038/s41388-020-1379-0 32655131 10.1038/s41388-020-1379-0 PMC7426263

[87] Hayes, D. F., Smerage, J. B. (2010). Circulating tumor cells, in: Progress in molecular biology and translational science, Elsevier pp. 95–112. 10.1016/B978-0-12-385071-3.00005-8. 10.1016/B978-0-12-385071-3.00005-8 21075330

[88] Krebs, M. G., Hou, J.-M., Ward, T. H., Blackhall, F. H., & Dive, C. (2010). Circulating tumour cells: Their utility in cancer management and predicting outcomes. *Therapeutic Advances in Medical Oncology,**2*, 351–365. 10.1177/1758834010378414 21789147 10.1177/1758834010378414 PMC3126032

[89] Miller, M. C., Doyle, G. V., & Terstappen, L. W. M. M. (2010). Significance of circulating tumor cells detected by the CellSearch system in patients with metastatic breast colorectal and prostate cancer. *Journal Of Oncology*. 10.1155/2010/617421 20016752 10.1155/2010/617421 PMC2793426

[90] Andree, K. C., van Dalum, G., & Terstappen, L. W. M. M. (2016). Challenges in circulating tumor cell detection by the Cell Search System. *Molecular Oncology,**10*, 395–407. 10.1016/j.molonc.2015.12.002 26795350 10.1016/j.molonc.2015.12.002 PMC5528971

[91] van der Toom, E. E., Verdone, J. E., Gorin, M. A., & Pienta, K. J. (2016). Technical challenges in the isolation and analysis of circulating tumor cells. *Oncotarget,**7*, Article 62754–62766. 10.18632/oncotarget.11191 27517159 10.18632/oncotarget.11191 PMC5308763

[92] Zou, D., & Cui, D. (2018). Advances in isolation and detection of circulating tumor cells based on microfluidics. *Cancer Biology & Medicine,**15*, 335–353. 10.20892/j.issn.2095-3941.2018.0256 30766747 10.20892/j.issn.2095-3941.2018.0256 PMC6372907

[93] Ashworth, T. R. (1869). A case of cancer in which cells similar to those in the tumors were seen in the blood after death. *Australasian Medical Journal,**14*, 146–149.

[94] Wong, K. H. K., Tessier, S. N., Miyamoto, D. T., Miller, K. L., Bookstaver, L. D., Carey, T. R., Stannard, C. J., Thapar, V., Tai, E. C., Vo, K. D., Emmons, E. S., Pleskow, H. M., Sandlin, R. D., Sequist, L. V., Ting, D. T., Haber, D. A., Maheswaran, S., Stott, S. L., & Toner, M. (2017). Whole blood stabilization for the microfluidic isolation and molecular characterization of circulating tumor cells. *Nature Communications,**8*, 1733. 10.1038/s41467-017-01705-y 29170510 10.1038/s41467-017-01705-y PMC5700979

[95] Miller, M., Robinson, P., Wagner, C., O’Shannessy, D. (2018). The Parsortix^TM^ cell separation system—A versatile liquid biopsy platform, Cytometry Part A 93. 10.1002/cyto.a.23571. 10.1002/cyto.a.23571 PMC6586069 30107082

[96] Tieng, F. Y. F., Abu, N., Nasir, S. N., Lee, L.-H., & Ab Mutalib, N.-S. (2021). Liquid biopsy-based colorectal cancer screening via surface markers of circulating tumor cells. *Diagnostics,**11*, Article 2136. 10.3390/diagnostics11112136 34829483 10.3390/diagnostics11112136 PMC8618170

[97] Sasagawa, Y., Nikaido, I., Hayashi, T., Danno, H., Uno, K. D., Imai, T., & Ueda, H. R. (2013). Quartz-Seq: A highly reproducible and sensitive single-cell RNA sequencing method, reveals non-genetic gene-expression heterogeneity. *Genome Biology,**14*, R31. 10.1186/gb-2013-14-4-r31 23594475 10.1186/gb-2013-14-4-r31 PMC4054835

[98] Sasagawa, Y., Danno, H., Takada, H., Ebisawa, M., Tanaka, K., Hayashi, T., Kurisaki, A., & Nikaido, I. (2018). Quartz-seq2: A high-throughput single-cell RNA-sequencing method that effectively uses limited sequence reads. *Genome Biology,**19*, 29. 10.1186/s13059-018-1407-3 29523163 10.1186/s13059-018-1407-3 PMC5845169

[99] Hagemann-Jensen, M., Ziegenhain, C., Chen, P., Ramsköld, D., Hendriks, G.-J., Larsson, A. J. M., Faridani, O. R., & Sandberg, R. (2020). Single-cell RNA counting at allele and isoform resolution using Smart-seq3. *Nature Biotechnology,**38*, 708–714. 10.1038/s41587-020-0497-0 32518404 10.1038/s41587-020-0497-0

[100] Bageritz, J., & Raddi, G. (2019). Single-cell RNA sequencing with Drop-Seq. In V. Proserpio (Ed.), 10.1007/978-1-4939-9240-9_6 31028633

[101] Macosko, E. Z., Basu, A., Satija, R., Nemesh, J., Shekhar, K., Goldman, M., Tirosh, I., Bialas, A. R., Kamitaki, N., Martersteck, E. M., Trombetta, J. J., Weitz, D. A., Sanes, J. R., Shalek, A. K., Regev, A., & McCarroll, S. A. (2015). Highly parallel genome-wide expression profiling of individual cells using nanoliter droplets. *Cell,**161*, 1202–1214. 10.1016/j.cell.2015.05.002 26000488 10.1016/j.cell.2015.05.002 PMC4481139

[102] Ziegenhain, C., Vieth, B., Parekh, S., Reinius, B., Guillaumet-Adkins, A., Smets, M., Leonhardt, H., Heyn, H., Hellmann, I., & Enard, W. (2017). Comparative analysis of single-cell RNA sequencing methods. *Molecular Cell,**65*, 631-643.e4. 10.1016/j.molcel.2017.01.023 28212749 10.1016/j.molcel.2017.01.023

[103] Shomroni, O., Sitte, M., Schmidt, J., Parbin, S., Ludewig, F., Yigit, G., Zelarayan, L. C., Streckfuss-Bömeke, K., Wollnik, B., & Salinas, G. (2022). A novel single-cell RNA-sequencing approach and its applicability connecting genotype to phenotype in ageing disease. *Scientific Reports,**12*, Article 4091. 10.1038/s41598-022-07874-1 35260714 10.1038/s41598-022-07874-1 PMC8904555

[104] Shum, E. Y., Walczak, E. M., Chang, C., & Christina Fan, H. (2019). Quantitation of mRNA transcripts and proteins using the BD Rhapsody^TM^ single-cell analysis system. *Advances in Experimental Medicine and Biology,**1129*, 63–79. 10.1007/978-981-13-6037-4_5 30968361 10.1007/978-981-13-6037-4_5

[105] Ramalingam, N., Fowler, B., Szpankowski, L., Leyrat, A. A., Hukari, K., Maung, M. T., Yorza, W., Norris, M., Cesar, C., Shuga, J., Gonzales, M. L., Sanada, C. D., Wang, X., Yeung, R., Hwang, W., Axsom, J., Devaraju, N. S. G. K., Angeles, N. D., Greene, C., … West, J. A. A. (2016). Fluidic logic used in a systems approach to enable integrated single-cell functional analysis. *Front Bioeng Biotechnol,**4*, 70. 10.3389/fbioe.2016.00070 27709111 10.3389/fbioe.2016.00070 PMC5030342

[106] LaMar, D. (2015). FastQC, https://qubeshub.org/resources/fastqc.

[107] Krueger, F. (2015). Trim Galore!: A wrapper around Cutadapt and FastQC to consistently apply adapter and quality trimming to FastQ files, with extra functionality for RRBS data, Babraham Institute. https://cir.nii.ac.jp/crid/1370294643762929691 (Accessed March 30, 2025).

[108] Bolger, A. M., Lohse, M., & Usadel, B. (2014). Trimmomatic: A flexible trimmer for Illumina sequence data. *Bioinformatics,**30*, 2114–2120. 10.1093/bioinformatics/btu170 24695404 10.1093/bioinformatics/btu170 PMC4103590

[109] Wingett, S. W., & Andrews, S. (2018). FastQ screen: A tool for multi-genome mapping and quality control. *F1000Research,**7*, Article 1338. 10.12688/f1000research.15931.2 30254741 10.12688/f1000research.15931.1 PMC6124377

[110] Dobin, A., Davis, C. A., Schlesinger, F., Drenkow, J., Zaleski, C., Jha, S., Batut, P., Chaisson, M., & Gingeras, T. R. (2013). STAR: Ultrafast universal RNA-seq aligner. *Bioinformatics,**29*, 15–21. 10.1093/bioinformatics/bts635 23104886 10.1093/bioinformatics/bts635 PMC3530905

[111] Patro, R., Duggal, G., Love, M. I., Irizarry, R. A., & Kingsford, C. (2017). Salmon provides fast and bias-aware quantification of transcript expression. *Nature Methods,**14*, 417–419. 10.1038/nmeth.4197 28263959 10.1038/nmeth.4197 PMC5600148

[112] Bray, N. L., Pimentel, H., Melsted, P., & Pachter, L. (2016). Near-optimal probabilistic RNA-seq quantification. *Nature Biotechnology,**34*(3), 525–527. 10.1038/nbt.3519 27043002 10.1038/nbt.3519

[113] Putri, G. H., Anders, S., Pyl, P. T., Pimanda, J. E., & Zanini, F. (2022). Analysing high-throughput sequencing data in Python with HTSeq 2.0. *Bioinformatics,**38*, 2943–2945. 10.1093/bioinformatics/btac166 35561197 10.1093/bioinformatics/btac166 PMC9113351

[114] Liao, Y., Smyth, G. K., & Shi, W. (2014). FeatureCounts: An efficient general purpose program for assigning sequence reads to genomic features. *Bioinformatics,**30*, 923–930. 10.1093/bioinformatics/btt656 24227677 10.1093/bioinformatics/btt656

[115] Wolock, S. L., Lopez, R., & Klein, A. M. (2019). Scrublet: Computational identification of cell doublets in single-cell transcriptomic data. *Cell Systems,**8*, 281-291.e9. 10.1016/j.cels.2018.11.005 30954476 10.1016/j.cels.2018.11.005 PMC6625319

[116] McGinnis, C. S., Murrow, L. M., & Gartner, Z. J. (2019). DoubletFinder: Doublet detection in single-cell RNA sequencing data using artificial nearest neighbors. *Cell Systems,**8*, 329-337.e4. 10.1016/j.cels.2019.03.003 30954475 10.1016/j.cels.2019.03.003 PMC6853612

[117] Choudhary, S., & Satija, R. (2022). Comparison and evaluation of statistical error models for scRNA-seq. *Genome Biology,**23*, Article 27. 10.1186/s13059-021-02584-9 35042561 10.1186/s13059-021-02584-9 PMC8764781

[118] Hafemeister, C., & Satija, R. (2019). Normalization and variance stabilization of single-cell RNA-seq data using regularized negative binomial regression. *Genome Biology,**20*, 296. 10.1186/s13059-019-1874-1 31870423 10.1186/s13059-019-1874-1 PMC6927181

[119] Haghverdi, L., Lun, A. T. L., Morgan, M. D., & Marioni, J. C. (2018). Batch effects in single-cell RNA-sequencing data are corrected by matching mutual nearest neighbors. *Nature Biotechnology,**36*, 421–427. 10.1038/nbt.4091 29608177 10.1038/nbt.4091 PMC6152897

[120] Hie, B., Bryson, B., & Berger, B. (2019). Efficient integration of heterogeneous single-cell transcriptomes using Scanorama. *Nature Biotechnology,**37*, 685–691. 10.1038/s41587-019-0113-3 31061482 10.1038/s41587-019-0113-3 PMC6551256

[121] Buettner, F., Natarajan, K. N., Casale, F. P., Proserpio, V., Scialdone, A., Theis, F. J., Teichmann, S. A., Marioni, J. C., & Stegle, O. (2015). Computational analysis of cell-to-cell heterogeneity in single-cell RNA-sequencing data reveals hidden subpopulations of cells. *Nature Biotechnology,**33*, 155–160. 10.1038/nbt.3102 25599176 10.1038/nbt.3102

[122] Li, W. V., & Li, J. J. (2018). An accurate and robust imputation method scImpute for single-cell RNA-seq data. *Nature Communications,**9*, 997. 10.1038/s41467-018-03405-7 29520097 10.1038/s41467-018-03405-7 PMC5843666

[123] Maćkiewicz, A., & Ratajczak, W. (1993). Principal components analysis (PCA). *Computers & Geosciences,**19*, 303–342. 10.1016/0098-3004(93)90090-R

[124] Healy, J., & McInnes, L. (2024). Uniform manifold approximation and projection. *Nature Reviews Methods Primers,**4*, 1–15. 10.1038/s43586-024-00363-x

[125] McInnes, L., Healy, J., Melville, J. (2020). UMAP: Uniform manifold approximation and projection for dimension reduction. 10.48550/arXiv.1802.03426.

[126] Blondel, V. D., Guillaume, J.-L., Lambiotte, R., & Lefebvre, E. (2008). Fast unfolding of communities in large networks. *Journal of Statistical Mechanics: Theory and Experiment,**2008*, Article P10008. 10.1088/1742-5468/2008/10/P10008

[127] Tang, M., Kaymaz, Y., Logeman, B. L., Eichhorn, S., Liang, Z. S., Dulac, C., & Sackton, T. B. (2021). Evaluating single-cell cluster stability using the Jaccard similarity index. *Bioinformatics,**37*, 2212–2214. 10.1093/bioinformatics/btaa956 33165513 10.1093/bioinformatics/btaa956 PMC8352506

[128] Trapnell, C., Cacchiarelli, D., Grimsby, J., Pokharel, P., Li, S., Morse, M., Lennon, N. J., Livak, K. J., Mikkelsen, T. S., & Rinn, J. L. (2014). The dynamics and regulators of cell fate decisions are revealed by pseudotemporal ordering of single cells. *Nature Biotechnology,**32*, 381–386. 10.1038/nbt.2859 24658644 10.1038/nbt.2859 PMC4122333

[129] Aran, D., Looney, A. P., Liu, L., Wu, E., Fong, V., Hsu, A., Chak, S., Naikawadi, R. P., Wolters, P. J., Abate, A. R., Butte, A. J., & Bhattacharya, M. (2019). Reference-based analysis of lung single-cell sequencing reveals a transitional profibrotic macrophage. *Nature Immunology,**20*, 163–172. 10.1038/s41590-018-0276-y 30643263 10.1038/s41590-018-0276-y PMC6340744

[130] Li, H., Courtois, E. T., Sengupta, D., Tan, Y., Chen, K. H., Goh, J. J. L., Kong, S. L., Chua, C., Hon, L. K., Tan, W. S., Wong, M., Choi, P. J., Wee, L. J. K., Hillmer, A. M., Tan, I. B., Robson, P., & Prabhakar, S. (2017). Reference component analysis of single-cell transcriptomes elucidates cellular heterogeneity in human colorectal tumors. *Nature Genetics,**49*, 708–718. 10.1038/ng.3818 28319088 10.1038/ng.3818

[131] Lun, A. T. L., McCarthy, D. J., & Marioni, J. C. (2016). A step-by-step workflow for low-level analysis of single-cell RNA-seq data with bioconductor. *F1000research,**5*, Article 2122. 10.12688/f1000research.9501.2 27909575 10.12688/f1000research.9501.1 PMC5112579

[132] Ritchie, M. E., Phipson, B., Wu, D., Hu, Y., Law, C. W., Shi, W., & Smyth, G. K. (2015). Limma powers differential expression analyses for RNA-sequencing and microarray studies. *Nucleic Acids Research,**43*, Article e47. 10.1093/nar/gkv007 25605792 10.1093/nar/gkv007 PMC4402510

[133] Yu, G., Wang, L.-G., Han, Y., & He, Q.-Y. (2012). ClusterProfiler: An R package for comparing biological themes among gene clusters. *Omics : A Journal of Integrative Biology,**16*, 284–287. 10.1089/omi.2011.0118 22455463 10.1089/omi.2011.0118 PMC3339379

[134] Li, B., & Dewey, C. N. (2011). Rsem: Accurate transcript quantification from rna-seq data with or without a reference genome. *Bmc Bioinformatics,**12*, 323. 10.1186/1471-2105-12-323 21816040 10.1186/1471-2105-12-323 PMC3163565

[135] Hänzelmann, S., Castelo, R., & Guinney, J. (2013). GSVA: Gene set variation analysis for microarray and RNA-Seq data. *Bmc Bioinformatics,**14*, 7. 10.1186/1471-2105-14-7 23323831 10.1186/1471-2105-14-7 PMC3618321

[136] Yu, G., & He, Q.-Y. (2016). ReactomePA: An R/Bioconductor package for reactome pathway analysis and visualization. *Molecular Biosystems,**12*, 477–479. 10.1039/c5mb00663e 26661513 10.1039/c5mb00663e

[137] Efremova, M., Vento-Tormo, M., Teichmann, S. A., & Vento-Tormo, R. (2020). Cell PhoneDB: Inferring cell–cell communication from combined expression of multi-subunit ligand–receptor complexes. *Nature Protocols,**15*, 1484–1506. 10.1038/s41596-020-0292-x 32103204 10.1038/s41596-020-0292-x

[138] Shen, S., Park, J. W., Lu, Z., Lin, L., Henry, M. D., Wu, Y. N., Zhou, Q., & Xing, Y. (2014). rMATS: Robust and flexible detection of differential alternative splicing from replicate RNA-Seq data. *Proceedings of the National Academy of Sciences,**111*, E5593–E5601. 10.1073/pnas.1419161111 10.1073/pnas.1419161111 PMC4280593 25480548

[139] Song, Y., Botvinnik, O. B., Lovci, M. T., Kakaradov, B., Liu, P., Xu, J. L., & Yeo, G. W. (2017). Single-cell alternative splicing analysis with expedition reveals splicing dynamics during neuron differentiation. *Molecular Cell,**67*, 148-161.e5. 10.1016/j.molcel.2017.06.003 28673540 10.1016/j.molcel.2017.06.003 PMC5540791

[140] Cao, J., Spielmann, M., Qiu, X., Huang, X., Ibrahim, D. M., Hill, A. J., Zhang, F., Mundlos, S., Christiansen, L., Steemers, F. J., Trapnell, C., & Shendure, J. (2019). The single-cell transcriptional landscape of mammalian organogenesis. *Nature,**566*, 496–502. 10.1038/s41586-019-0969-x 30787437 10.1038/s41586-019-0969-x PMC6434952

[141] Satija, R., Farrell, J. A., Gennert, D., Schier, A. F., & Regev, A. (2015). Spatial reconstruction of single-cell gene expression data. *Nature Biotechnology,**33*, 495–502. 10.1038/nbt.3192 25867923 10.1038/nbt.3192 PMC4430369

[142] Finak, G., McDavid, A., Yajima, M., Deng, J., Gersuk, V., Shalek, A. K., Slichter, C. K., Miller, H. W., McElrath, M. J., Prlic, M., Linsley, P. S., & Gottardo, R. (2015). MAST: A flexible statistical framework for assessing transcriptional changes and characterizing heterogeneity in single-cell RNA sequencing data. *Genome Biology,**16*, 278. 10.1186/s13059-015-0844-5 26653891 10.1186/s13059-015-0844-5 PMC4676162

[143] Zhang, Y., Ma, Y., Huang, Y., Zhang, Y., Jiang, Q., Zhou, M., & Su, J. (2020). Benchmarking algorithms for pathway activity transformation of single-cell RNA-seq data. *Computational and Structural Biotechnology Journal,**18*, 2953–2961. 10.1016/j.csbj.2020.10.007 33209207 10.1016/j.csbj.2020.10.007 PMC7642725

[144] Feng, H., Lin, L., & Chen, J. (2022). ScDIOR: Single cell RNA-seq data IO software. *BMC Bioinformatics,**23*, 16. 10.1186/s12859-021-04528-3 34991457 10.1186/s12859-021-04528-3 PMC8734364

[145] Wang, L., Wang, S., & Li, W. (2012). RSeQC: Quality control of RNA-seq experiments. *Bioinformatics,**28*, 2184–2185. 10.1093/bioinformatics/bts356 22743226 10.1093/bioinformatics/bts356

[146] McCarthy, D. J., Campbell, K. R., Lun, A. T. L., & Wills, Q. F. (2017). Scater: Pre-processing, quality control, normalization and visualization of single-cell RNA-seq data in R. *Bioinformatics,**33*, 1179–1186. 10.1093/bioinformatics/btw777 28088763 10.1093/bioinformatics/btw777 PMC5408845

[147] Chen, Y., Chen, Y., Shi, C., Huang, Z., Zhang, Y., Li, S., Li, Y., Ye, J., Yu, C., Li, Z., Zhang, X., Wang, J., Yang, H., Fang, L., & Chen, Q. (2018). SOAPnuke: A MapReduce acceleration-supported software for integrated quality control and preprocessing of high-throughput sequencing data. *Gigascience,**7*, Article gix120. 10.1093/gigascience/gix120 29220494 10.1093/gigascience/gix120 PMC5788068

[148] Danecek, P., Bonfield, J. K., Liddle, J., Marshall, J., Ohan, V., Pollard, M. O., Whitwham, A., Keane, T., McCarthy, S. A., Davies, R. M., & Li, H. (2021). Twelve years of SAMtools and BCFtools. *Gigascience,**10*, Article giab008. 10.1093/gigascience/giab008 33590861 10.1093/gigascience/giab008 PMC7931819

[149] Ewels, P., Magnusson, M., Lundin, S., & Käller, M. (2016). MultiQC: Summarize analysis results for multiple tools and samples in a single report. *Bioinformatics,**32*, 3047–3048. 10.1093/bioinformatics/btw354 27312411 10.1093/bioinformatics/btw354 PMC5039924

[150] Smith, T., Heger, A., & Sudbery, I. (2017). UMI-tools: Modeling sequencing errors in Unique Molecular Identifiers to improve quantification accuracy. *Genome Research,**27*, 491–499. 10.1101/gr.209601.116 28100584 10.1101/gr.209601.116 PMC5340976

[151] Parekh, S., Ziegenhain, C., Vieth, B., Enard, W., & Hellmann, I. (2018). zUMIs - A fast and flexible pipeline to process RNA sequencing data with UMIs. *Gigascience,**7*, Article giy059. 10.1093/gigascience/giy059 29846586 10.1093/gigascience/giy059 PMC6007394

[152] Wang, Z., Hu, J., Johnson, W. E., & Campbell, J. D. (2019). Scruff: An R/Bioconductor package for preprocessing single-cell RNA-sequencing data. *Bmc Bioinformatics,**20*, 222. 10.1186/s12859-019-2797-2 31046658 10.1186/s12859-019-2797-2 PMC6498700

[153] Kim, D., Pertea, G., Trapnell, C., Pimentel, H., Kelley, R., & Salzberg, S. L. (2013). Tophat2: Accurate alignment of transcriptomes in the presence of insertions, deletions and gene fusions. *Genome Biology,**14*, R36. 10.1186/gb-2013-14-4-r36 23618408 10.1186/gb-2013-14-4-r36 PMC4053844

[154] Trapnell, C., Pachter, L., & Salzberg, S. L. (2009). TopHat. *Bioinformatics,**25*, 1105–1111. 10.1093/bioinformatics/btp120 19289445 10.1093/bioinformatics/btp120 PMC2672628

[155] Langmead, B., Trapnell, C., Pop, M., & Salzberg, S. L. (2009). Ultrafast and memory-efficient alignment of short DNA sequences to the human genome. *Genome Biology,**10*, R25. 10.1186/gb-2009-10-3-r25 19261174 10.1186/gb-2009-10-3-r25 PMC2690996

[156] Langmead, B., & Salzberg, S. L. (2012). Fast gapped-read alignment with Bowtie 2. *Nature Methods,**9*, 357–359. 10.1038/nmeth.1923 22388286 10.1038/nmeth.1923 PMC3322381

[157] Kim, D., Paggi, J. M., Park, C., Bennett, C., & Salzberg, S. L. (2019). Graph-based genome alignment and genotyping with HISAT2 and HISAT-genotype. *Nature Biotechnology,**37*, 907–915. 10.1038/s41587-019-0201-4 31375807 10.1038/s41587-019-0201-4 PMC7605509

[158] Yip, S. H., Wang, P., Kocher, J.-P.A., Sham, P. C., & Wang, J. (2017). Linnorm: Improved statistical analysis for single cell RNA-seq expression data. *Nucleic Acids Research,**45*, Article e179. 10.1093/nar/gkx828 28981748 10.1093/nar/gkx828 PMC5727406

[159] van der Maaten, L., & Hinton, G. (2008). Viualizing data using t-SNE. *Journal of Machine Learning Research,**9*, 2579–2605.

[160] Risso, D., Perraudeau, F., Gribkova, S., Dudoit, S., & Vert, J.-P. (2018). A general and flexible method for signal extraction from single-cell RNA-seq data. *Nature Communications,**9*, 284. 10.1038/s41467-017-02554-5 29348443 10.1038/s41467-017-02554-5 PMC5773593

[161] Traag, V. A., Waltman, L., & van Eck, N. J. (2019). From Louvain to Leiden: Guaranteeing well-connected communities. *Scientific Reports,**9*, 5233. 10.1038/s41598-019-41695-z 30914743 10.1038/s41598-019-41695-z PMC6435756

[162] Kiselev, V. Y., Kirschner, K., Schaub, M. T., Andrews, T., Yiu, A., Chandra, T., Natarajan, K. N., Reik, W., Barahona, M., Green, A. R., & Hemberg, M. (2017). SC3: Consensus clustering of single-cell RNA-seq data. *Nature Methods,**14*, 483–486. 10.1038/nmeth.4236 28346451 10.1038/nmeth.4236 PMC5410170

[163] Heaton, H., Talman, A. M., Knights, A., Imaz, M., Gaffney, D. J., Durbin, R., Hemberg, M., & Lawniczak, M. K. N. (2020). Souporcell: Robust clustering of single-cell RNA-seq data by genotype without reference genotypes. *Nature Methods,**17*, 615–620. 10.1038/s41592-020-0820-1 32366989 10.1038/s41592-020-0820-1 PMC7617080

[164] Liu, Y., Wang, T., Zhou, B., & Zheng, D. (2021). Robust integration of multiple single-cell RNA sequencing datasets using a single reference space. *Nature Biotechnology,**39*, 877–884. 10.1038/s41587-021-00859-x 33767393 10.1038/s41587-021-00859-x PMC8456427

[165] Korsunsky, I., Millard, N., Fan, J., Slowikowski, K., Zhang, F., Wei, K., Baglaenko, Y., Brenner, M., Loh, P.-R., & Raychaudhuri, S. (2019). Fast, sensitive and accurate integration of single-cell data with harmony. *Nature Methods,**16*, 1289–1296. 10.1038/s41592-019-0619-0 31740819 10.1038/s41592-019-0619-0 PMC6884693

[166] Pliner, H. A., Shendure, J., & Trapnell, C. (2019). Supervised classification enables rapid annotation of cell atlases. *Nature Methods,**16*, 983–986. 10.1038/s41592-019-0535-3 31501545 10.1038/s41592-019-0535-3 PMC6791524

[167] Li, C., Liu, B., Kang, B., Liu, Z., Liu, Y., Chen, C., Ren, X., & Zhang, Z. (2020). SciBet as a portable and fast single cell type identifier. *Nature Communications,**11*, 1818. 10.1038/s41467-020-15523-2 32286268 10.1038/s41467-020-15523-2 PMC7156687

[168] Chen, Y., Chen, L., Lun, A. T. L., Baldoni, P. L., & Smyth, G. K. (2025). edgeR v4: Powerful differential analysis of sequencing data with expanded functionality and improved support for small counts and larger datasets. *Nucleic Acids Research,**53*, gkaf018. 10.1093/nar/gkaf018 39844453 10.1093/nar/gkaf018 PMC11754124

[169] Kuleshov, M. V., Jones, M. R., Rouillard, A. D., Fernandez, N. F., Duan, Q., Wang, Z., Koplev, S., Jenkins, S. L., Jagodnik, K. M., Lachmann, A., McDermott, M. G., Monteiro, C. D., Gundersen, G. W., & Ma’ayan, A. (2016). Enrichr: A comprehensive gene set enrichment analysis web server 2016 update. *Nucleic Acids Research,**44*, 90–97. 10.1093/nar/gkw377 10.1093/nar/gkw377 PMC4987924 27141961

[170] Love, M. I., Huber, W., & Anders, S. (2014). Moderated estimation of fold change and dispersion for RNA-seq data with DESeq2. *Genome Biology*. 10.1186/s13059-014-0550-8 25516281 10.1186/s13059-014-0550-8 PMC4302049

[171] Langfelder, P., & Horvath, S. (2008). WGCNA: An R package for weighted correlation network analysis. *BMC Bioinformatics,**9*, 559. 10.1186/1471-2105-9-559 19114008 10.1186/1471-2105-9-559 PMC2631488

[172] Fan, J., Salathia, N., Liu, R., Kaeser, G. E., Yung, Y. C., Herman, J. L., Kaper, F., Fan, J.-B., Zhang, K., Chun, J., & Kharchenko, P. V. (2016). Characterizing transcriptional heterogeneity through pathway and gene set overdispersion analysis. *Nature Methods,**13*, 241–244. 10.1038/nmeth.3734 26780092 10.1038/nmeth.3734 PMC4772672

[173] Kharchenko, P. V., Silberstein, L., & Scadden, D. T. (2014). Bayesian approach to single-cell differential expression analysis. *Nature Methods,**11*, 740–742. 10.1038/nmeth.2967 24836921 10.1038/nmeth.2967 PMC4112276

[174] Zhou, Y., Zhou, B., Pache, L., Chang, M., Khodabakhshi, A. H., Tanaseichuk, O., Benner, C., & Chanda, S. K. (2019). Metascape provides a biologist-oriented resource for the analysis of systems-level datasets. *Nature Communications,**10*, 1523. 10.1038/s41467-019-09234-6 30944313 10.1038/s41467-019-09234-6 PMC6447622

[175] Korotkevich, G., Sukhov, V., Budin, N., Shpak, B., Artyomov, M. (2016) A. Sergushichev, Fast gene set enrichment analysi. 10.1101/060012.

[176] Zhang, B., Kirov, S., & Snoddy, J. (2005). WebGestalt: An integrated system for exploring gene sets in various biological contexts. *Nucleic Acids Research,**33*, W741-748. 10.1093/nar/gki475 15980575 10.1093/nar/gki475 PMC1160236

[177] Ji, Z., Vokes, S. A., Dang, C. V., & Ji, H. (2016). Turning publicly available gene expression data into discoveries using gene set context analysis. *Nucleic Acids Research,**44*, Article e8. 10.1093/nar/gkv873 26350211 10.1093/nar/gkv873 PMC4705686

[178] Sherman, B. T., Hao, M., Qiu, J., Jiao, X., Baseler, M. W., Lane, H. C., Imamichi, T., & Chang, W. (2022). DAVID: A web server for functional enrichment analysis and functional annotation of gene lists (2021 update). *Nucleic Acids Research,**50*(W1), W216–W221. 10.1093/nar/gkac194 35325185 10.1093/nar/gkac194 PMC9252805

[179] Jiang, A., Snell, R. G., & Lehnert, K. (2024). ICARUS v3, a massively scalable web server for single-cell RNA-seq analysis of millions of cells. *Bioinformatics,**40*, Article btae167. 10.1093/bioinformatics/btae167 38539041 10.1093/bioinformatics/btae167 PMC11007236

[180] Law, V., Chen, Z., Vena, F., Smalley, I., Macaulay, R., Evernden, B. R., Tran, N., Pina, Y., Puskas, J., Caceres, G., Bayle, S., Johnson, J., Liu, J. K. C., Etame, A., Vogelbaum, M., Rodriguez, P., Duckett, D., Czerniecki, B., Chen, A., … Forsyth, P. A. (2022). A preclinical model of patient-derived cerebrospinal fluid circulating tumor cells for experimental therapeutics in leptomeningeal disease from melanoma. *Neuro-Oncology*. 10.1093/neuonc/noac054 35213727 10.1093/neuonc/noac054 PMC9527526

[181] Yang, Z., Bai, H., Wu, Q., Hu, L., Li, G., Kong, D., Zhang, Q., Wan, D., Gu, Z., Zhao, C., Zhang, K., Zhang, W., & Shou, J. (2022). Telomerase positive CTCs with PSMA high expression associated with prostate cancer metastasis. *Translational Andrology and Urology,**11*, 803–813. 10.21037/tau-21-1140 35812202 10.21037/tau-21-1140 PMC9262742

[182] Sun, Y., Li, T., Ding, L., Wang, J., Chen, C., Liu, T., Liu, Y., Li, Q., Wang, C., Huo, R., Wang, H., Tian, T., Zhang, C., Pan, B., Zhou, J., Fan, J., Yang, X., Yang, W., Wang, B., & Guo, W. (2025). Platelet-mediated circulating tumor cell evasion from natural killer cell killing through immune checkpoint CD155-TIGIT. *Hepatology,**81*, 791–807. 10.1097/HEP.0000000000000934 38779918 10.1097/HEP.0000000000000934

[183] Schmidt, M. J., Naghdloo, A., Prabakar, R. K., Kamal, M., Cadaneanu, R., Garraway, I. P., Lewis, M., Aparicio, A., Zurita-Saavedra, A., Corn, P., Kuhn, P., Pienta, K. J., Amend, S. R., & Hicks, J. (2025). Polyploid cancer cells reveal signatures of chemotherapy resistance. *Oncogene,**44*, 439–449. 10.1038/s41388-024-03212-z 39578659 10.1038/s41388-024-03212-z PMC11810791

[184] Arnault, J.-P., Chemmama, K., Ferroudj, K., Demagny, J., Panicot-Dubois, L., Galmiche, A., & Saidak, Z. (2025). The dynamic landscape of the coagulome of metastatic malignant melanoma. *International Journal of Molecular Sciences,**26*, Article 1435. 10.3390/ijms26041435 40003901 10.3390/ijms26041435 PMC11855523

[185] Yang, H., Wang, H., He, Y., Yang, Y., Thompson, E. W., Xia, D., Burke, L. J., Cao, L., Hooper, J. D., Roberts, M. S., Crawford, D. H. G., & Liang, X. (2024). Identification and characterization of TM4SF1+ tumor self-seeded cells. *Cell Reports*. 10.1016/j.celrep.2024.114512 39003738 10.1016/j.celrep.2024.114512

[186] Nian, Z., Wang, D., Wang, H., Liu, W., Ma, Z., Yan, J., Cao, Y., Li, J., Zhao, Q., & Liu, Z. (2024). Single-cell RNA-seq reveals the transcriptional program underlying tumor progression and metastasis in neuroblastoma. *Frontiers in Medicine,**18*, 690–707. 10.1007/s11684-024-1081-7 10.1007/s11684-024-1081-7 39014137

[187] Suvilesh, K. N., Nussbaum, Y. I., Radhakrishnan, V., Manjunath, Y., Avella, D. M., Staveley-O’Carroll, K. F., Kimchi, E. T., Chaudhuri, A. A., Shyu, C.-R., Li, G., Pantel, K., Warren, W. C., Mitchem, J. B., & Kaifi, J. T. (2022). Tumorigenic circulating tumor cells from xenograft mouse models of non-metastatic NSCLC patients reveal distinct single cell heterogeneity and drug responses. *Molecular Cancer,**21*, Article 73. 10.1186/s12943-022-01553-5 35279152 10.1186/s12943-022-01553-5 PMC8917773

[188] Zheng, W.-J., Wang, P.-X., Sun, Y.-F., Cheng, J.-W., Zhong, Y.-C., Xu, Y., Guo, W., Hu, B., Zhou, J., Fan, J., Chen, X., & Yang, X.-R. (2022). Uncovering the heterogeneity and clinical relevance of circulating tumor-initiating cells in hepatocellular carcinoma using an integrated immunomagnetic-microfluidic platform. *ACS Applied Materials & Interfaces,**14*, 36425–36437. 10.1021/acsami.2c09085 35917454 10.1021/acsami.2c09085

[189] Pang, T. C. Y., Xu, Z., Mekapogu, A. R., Pothula, S., Becker, T., Corley, S., Wilkins, M. R., Goldstein, D., Pirola, R., Wilson, J., & Apte, M. (2021). HGF/c-Met inhibition as adjuvant therapy improves outcomes in an orthotopic mouse model of pancreatic cancer. *Cancers,**13*, Article 2763. 10.3390/cancers13112763 34199452 10.3390/cancers13112763 PMC8199621

[190] Sutton, T. L., Patel, R. K., Anderson, A. N., Bowden, S. G., Whalen, R., Giske, N. R., & Wong, M. H. (2022). Circulating cells with macrophage-like characteristics in cancer: The importance of circulating neoplastic-immune hybrid cells in cancer. *Cancers,**14*, Article 3871. 10.3390/cancers14163871 36010865 10.3390/cancers14163871 PMC9405966

[191] Anderson, A. N., Conley, P., Klocke, C. D., Sengupta, S. K., Pang, A., Farley, H. C., Gillingham, A. R., Dawson, A. D., Fan, Y., Jones, J. A., Gibbs, S. L., Skalet, A. H., Wu, G., & Wong, M. H. (2024). Detection of neoplastic-immune hybrid cells with metastatic properties in uveal melanoma. *Biomarker Research,**12*, 67. 10.1186/s40364-024-00609-6 39030653 10.1186/s40364-024-00609-6 PMC11264923

[192] Ali, A. M., & Raza, A. (2024). Scrnaseq and high-throughput spatial analysis of tumor and normal microenvironment in solid tumors reveal a possible origin of circulating tumor hybrid cells. *Cancers,**16*, Article 1444. 10.3390/cancers16071444 38611120 10.3390/cancers16071444 PMC11010995

[193] Ye, X., Huang, X., Fu, X., Zhang, X., Lin, R., Zhang, W., Zhang, J., & Lu, Y. (2023). Myeloid-like tumor hybrid cells in bone marrow promote progression of prostate cancer bone metastasis. *Journal of Hematology & Oncology,**16*, 46. 10.1186/s13045-023-01442-4 37138326 10.1186/s13045-023-01442-4 PMC10155318

[194] Minowa, T., Hirohashi, Y., Murata, K., Sasaki, K., Handa, T., Nakatsugawa, M., Mizue, Y., Murai, A., Kubo, T., Kanaseki, T., Tsukahara, T., Iwabuchi, S., Hashimoto, S., Ishida-Yamamoto, A., Uhara, H., & Torigoe, T. (2023). Fusion with type 2 macrophages induces melanoma cell heterogeneity that potentiates immunological escape from cytotoxic T lymphocytes. *The Journal of Pathology,**260*, 304–316. 10.1002/path.6083 37138382 10.1002/path.6083

[195] Menyailo, M. E., Zainullina, V. R., Khozyainova, A. A., Tashireva, L. A., Zolotareva, SYu. ., Gerashchenko, T. S., Alifanov, V. V., Savelieva, O. E., Grigoryeva, E. S., Tarabanovskaya, N. A., Popova, N. O., Choinzonov, E. L., Cherdyntseva, N. V., Perelmuter, V. M., & Denisov, E. V. (2023). Heterogeneity of circulating epithelial cells in breast cancer at single-cell resolution: Identifying tumor and hybrid cells. *Advanced Biology,**7*, Article 2200206. 10.1002/adbi.202200206 10.1002/adbi.202200206 36449636

[196] Chen, L., Lee, J. W., Chou, C.-L., Nair, A. V., Battistone, M. A., Păunescu, T. G., Merkulova, M., Breton, S., Verlander, J. W., Wall, S. M., Brown, D., Burg, M. B., & Knepper, M. A. (2017). Transcriptomes of major renal collecting duct cell types in mouse identified by single-cell RNA-seq. *Proceedings of the National Academy of Sciences of the United States of America,**114*, E9989–E9998. 10.1073/pnas.1710964114 29089413 10.1073/pnas.1710964114 PMC5699061

[197] Tirosh, I., Izar, B., Prakadan, S. M., Wadsworth, M. H., Treacy, D., Trombetta, J. J., Rotem, A., Rodman, C., Lian, C., Murphy, G., Fallahi-Sichani, M., Dutton-Regester, K., Lin, J.-R., Cohen, O., Shah, P., Lu, D., Genshaft, A. S., Hughes, T. K., Ziegler, C. G. K., … Garraway, L. A. (2016). Dissecting the multicellular ecosystem of metastatic melanoma by single-cell RNA-Seq. *Science,**352*, 189–196. 10.1126/science.aad0501 27124452 10.1126/science.aad0501 PMC4944528

[198] Lee, H.-O., Hong, Y., Etlioglu, H. E., Cho, Y. B., Pomella, V., Van den Bosch, B., Vanhecke, J., Verbandt, S., Hong, H., Min, J.-W., Kim, N., Eum, H. H., Qian, J., Boeckx, B., Lambrechts, D., Tsantoulis, P., De Hertogh, G., Chung, W., Lee, T., … Park, W.-Y. (2020). Lineage-dependent gene expression programs influence the immune landscape of colorectal cancer. *Nature Genetics,**52*, 594–603. 10.1038/s41588-020-0636-z 32451460 10.1038/s41588-020-0636-z

[199] Ma, L., Hernandez, M. O., Zhao, Y., Mehta, M., Tran, B., Kelly, M., Rae, Z., Hernandez, J. M., Davis, J. L., Martin, S. P., Kleiner, D. E., Hewitt, S. M., Ylaya, K., Wood, B. J., Greten, T. F., & Wang, X. W. (2019). Tumor cell biodiversity drives microenvironmental reprogramming in liver cancer. *Cancer Cell,**36*, 418-430.e6. 10.1016/j.ccell.2019.08.007 31588021 10.1016/j.ccell.2019.08.007 PMC6801104

[200] Laughney, A. M., Hu, J., Campbell, N. R., Bakhoum, S. F., Setty, M., Lavallée, V.-P., Xie, Y., Masilionis, I., Carr, A. J., Kottapalli, S., Allaj, V., Mattar, M., Rekhtman, N., Xavier, J. B., Mazutis, L., Poirier, J. T., Rudin, C. M., Pe’er, D., & Massagué, J. (2020). Regenerative lineages and immune-mediated pruning in lung cancer metastasis. *Nature Medicine,**26*(2), 259–269. 10.1038/s41591-019-0750-6 32042191 10.1038/s41591-019-0750-6 PMC7021003

[201] Durante, M. A., Rodriguez, D. A., Kurtenbach, S., Kuznetsov, J. N., Sanchez, M. I., Decatur, C. L., Snyder, H., Feun, L. G., Livingstone, A. S., & Harbour, J. W. (2020). Single-cell analysis reveals new evolutionary complexity in uveal melanoma. *Nature Communications,**11*, Article 496. 10.1038/s41467-019-14256-1 31980621 10.1038/s41467-019-14256-1 PMC6981133

[202] Hu, D. N., McCormick, S. A., Ritch, R., & Pelton-Henrion, K. (1993). Studies of human uveal melanocytes in vitro: Isolation, purification and cultivation of human uveal melanocytes. *Investigative Ophthalmology & Visual Science,**34*, 2210–2219. 8505203

[203] Pelka, K., Hofree, M., Chen, J. H., Sarkizova, S., Pirl, J. D., Jorgji, V., Bejnood, A., Dionne, D., Ge, W. H., Xu, K. H., Chao, S. X., Zollinger, D. R., Lieb, D. J., Reeves, J. W., Fuhrman, C. A., Hoang, M. L., Delorey, T., Nguyen, L. T., Waldman, J., … Hacohen, N. (2021). Spatially organized multicellular immune hubs in human colorectal cancer. *Cell,**184*, 4734-4752.e20. 10.1016/j.cell.2021.08.003 34450029 10.1016/j.cell.2021.08.003 PMC8772395

[204] Wu, S. Z., Al-Eryani, G., Roden, D., Junankar, S., Harvey, K., Andersson, A., Thennavan, A., Wang, C., Torpy, J., Bartonicek, N., Wang, T., Larsson, L., Kaczorowski, D., Weisenfeld, N. I., Uytingco, C. R., Chew, J. G., Bent, Z. W., Chan, C.-L., Gnanasambandapillai, V., … Swarbrick, A. (2021). A single-cell and spatially resolved atlas of human breast cancers. *Nature Genetics,**53*, 1334–1347. 10.1038/s41588-021-00911-1 34493872 10.1038/s41588-021-00911-1 PMC9044823

[205] Kfoury, Y., Baryawno, N., Severe, N., Mei, S., Gustafsson, K., Hirz, T., Brouse, T., Scadden, E. W., Igolkina, A. A., Kokkaliaris, K., Choi, B. D., Barkas, N., Randolph, M. A., Shin, J. H., Saylor, P. J., Scadden, D. T., Sykes, D. B., & Kharchenko, P. V. (2021). Human prostate cancer bone metastases have an actionable immunosuppressive microenvironment. *Cancer Cell,**39*, 1464-1478.e8. 10.1016/j.ccell.2021.09.005 34719426 10.1016/j.ccell.2021.09.005 PMC8578470

[206] Wang, Y., Tian, X., & Ai, D. (2021). Cell heterogeneity analysis in single-cell RNA-seq data using mixture exponential graph and Markov random field model. *BioMed Research International,**2021*, Article 9919080. 10.1155/2021/9919080 34095314 10.1155/2021/9919080 PMC8164540

[207] Thong, T., Wang, Y., Brooks, M. D., Lee, C. T., Scott, C., Balzano, L., Wicha, M. S., & Colacino, J. A. (2020). Hybrid stem cell states: Insights into the relationship between mammary development and breast cancer using single-cell transcriptomics. *Frontiers in Cell and Developmental Biology,**8*, 288. 10.3389/fcell.2020.00288 32457901 10.3389/fcell.2020.00288 PMC7227401

[208] Guo, X., Lin, F., Yi, C., Song, J., Sun, D., Lin, L., Zhong, Z., Wu, Z., Wang, X., Zhang, Y., Li, J., Zhang, H., Liu, F., Yang, C., & Song, J. (2022). Deep transfer learning enables lesion tracing of circulating tumor cells. *Nature Communications,**13*, 7687. 10.1038/s41467-022-35296-0 36509761 10.1038/s41467-022-35296-0 PMC9744915

[209] Del Giudice, M., Peirone, S., Perrone, S., Priante, F., Varese, F., Tirtei, E., Fagioli, F., & Cereda, M. (2021). Artificial intelligence in bulk and single-cell RNA-sequencing data to foster precision oncology. *International Journal of Molecular Sciences,**22*(9), Article 4563. 10.3390/ijms22094563 33925407 10.3390/ijms22094563 PMC8123853

[210] Karaayvaz, M., Cristea, S., Gillespie, S. M., Patel, A. P., Mylvaganam, R., Luo, C. C., Specht, M. C., Bernstein, B. E., Michor, F., & Ellisen, L. W. (2018). Unravelling subclonal heterogeneity and aggressive disease states in TNBC through single-cell RNA-seq. *Nature Communications,**9*, Article 3588. 10.1038/s41467-018-06052-0 30181541 10.1038/s41467-018-06052-0 PMC6123496

[211] Lee, Y., Guan, G., & Bhagat, A. A. (2018). ClearCell® FX, a label-free microfluidics technology for enrichment of viable circulating tumor cells. *Cytometry Part A,**93*, 1251–1254. 10.1002/cyto.a.23507 10.1002/cyto.a.23507 30080307

